# Targeting FGFR signaling overcomes therapeutic resistance and immune evasion in oncogenic PIK3CA-driven serous-like endometrial cancer

**DOI:** 10.1038/s41467-026-72544-z

**Published:** 2026-05-04

**Authors:** Xin Cheng, Yadong Zhang, Changli Qian, Erica Holdridge, Guruprasad Ananda, Tao Jiang, Jing Ni, Shaozhen Xie, Hao Gu, Renlei Ji, Elena V. Ivanova, Marisa R. Nucci, Zhe Wang, Kaifu Chen, Bose Kochupurakkal, Gordon J. Freeman, Geoffrey I. Shapiro, Joyce Liu, Panagiotis A. Konstantinopoulos, Ursula Matulonis, Jean J. Zhao

**Affiliations:** 1 Department of Cancer Biology, Dana-Farber Cancer Institute, Boston, MA USA; 2 Department of Biological Chemistry and Molecular Pharmacology, Harvard Medical School, Boston, MA USA; 3 Basic and Translational Research Division, Department of Cardiology, Boston Children’s Hospital, Boston, MA USA; 4 Department of Data Science, Dana-Farber Cancer Institute, Boston, MA USA; 5 Belfer Center for Applied Cancer Science, Dana-Farber Cancer Institute, Boston, MA USA; 6 Department of Medicine, Brigham and Women’s Hospital, Harvard Medical School, Boston, MA USA; 7 Department of Pathology, Brigham and Women’s Hospital, Harvard Medical School, Boston, MA USA; 8 Division of Gynecologic Oncology, Department of Medical Oncology, Dana-Farber Cancer Institute, Boston, MA USA; 9 Broad Institute of Harvard and MIT, Cambridge, MA USA; 10 Laboratory of Systems Pharmacology, Harvard Medical School, Boston, MA USA

**Keywords:** Cancer models, Cancer therapeutic resistance, Cancer immunotherapy

## Abstract

Serous endometrial cancer (SEC) is an aggressive subtype of endometrial cancer (EC) with poor prognosis and limited treatment options. Here, we develop a clinically relevant, immunocompetent serous-like mouse model incorporating oncogenic *PIK3CA* mutation, *Trp53* loss, and *MYC* overexpression. Using this model together with human EC cell lines, patient-derived organoids (PDOs), xenografts, and patient datasets, we investigate mechanisms underlying resistance to PI3Kα-targeted therapy. Single-cell profiling reveals that FGFR1/2 upregulation associates with intrinsic resistance, whereas FGFR3 characterizes acquired resistance. Dual FGFR and PI3Kα inhibition produces superior tumor control compared with either agent alone. Mechanistically, FGFR signaling promotes immune evasion by downregulating MHC-I/HLA-mediated antigen presentation and enriching M2-type tumor-associated macrophages. FGFR inhibition reverses these changes and synergizes with anti-PD-1 therapy to enhance antitumor immune responses and establish durable immune memory. Collectively, these findings identify FGFR signaling as a key driver of therapeutic resistance and immune escape in SEC and support FGFR-targeted combination strategies.

## Abstract (web summary)

PI3K has emerged as a potential therapeutic target for serious endometrial cancer but efficacy and duration of response is limited. Here, the authors develop a serous endometrial cancer murine model driven by PIK3CA mutation, TP53 loss and MYC overexpression, identifying FGFR pathway activation as a driver of PI3K-targeted therapeutic resistance and immune suppression.

## Introduction

Endometrial cancer (EC) is the most common gynecologic malignancy in the United States, with incidence and mortality rates continuing to rise^1^. SEC, a particularly aggressive subtype, accounts for nearly 40% of EC-related deaths^2^. SEC is characterized by frequent TP53 mutations ( ~ 90%) and a “serous-like” molecular profile with extensive copy-number alterations, and is associated with markedly reduced overall survival^3^. SEC also exhibits frequent alterations in the PI3K pathway, including mutations in PIK3CA (48%) and PIK3R1 (13.3%), as well as additional aberrations such as FGFR overexpression or amplification and MYC amplification (44%)^4,5^.

Current treatment options for SEC remain limited. Standard chemotherapy yields response rates of only 10-15% and a median overall survival of 8-12 months in advanced disease^6,7^. Immune checkpoint inhibitor (ICI) monotherapy shows similarly poor response rates (2.8-6.3%)^8–10^. Lenvatinib, a multiple tyrosine kinase inhibitor targeting FGFR1-4, VEGFR1-3, RET, and KIT, has demonstrated clinical activity in phase II/III trials^11,12^. Although the FDA-approved pembrolizumab-lenvatinib combination improves response rates to ~38%, treatment is often limited by significant toxicity, and approximately 40% of patients eventually experience disease progression. The absence of predictive biomarkers further complicates treatment selection^13–15^.

The phosphoinositide-3 kinase (PI3K) pathway is a crucial therapeutic target in SEC, with mutations present in 93% of EC cases^4,16^. Among these, PIK3CA mutations, predominantly in the helical region (E545K, E542K) and the kinase domain (H1047R), lead to the constitutive activation of PI3Kα, promoting tumorigenesis and disease progression^17–19^. Although several PI3Kα-selective inhibitors, including alpelisib (BYL719), copanlisib, inavolisib (GDC-0077), and STX-478, have shown clinical efficacy in advanced solid tumors and lymphoma^20–23^, PI3K inhibitors in endometrial cancer including serous endometrial cancers have demonstrated limited activity^24–26^. Several mechanisms of resistance to PIK3CA-targeted therapies have been reported, including feedback activation of AXL/EGFR/PKC signaling, compensatory activation of PI3Kβ in PTEN-deficient tumors, and reactivation of parallel MAPK and mTORC1 pathways^27–30^. Additional studies revealed metabolic and kinase rewiring involving PIM1 upregulation and insulin/IGF feedback loops, that restore AKT-mTOR signaling and promote tumor survival despite PI3K inhibition^31,32^. However, the specific mechanisms of resistance in EC remain poorly understood.

In this study, we develop a clinically relevant immunocompetent transgenic mouse model of serous-like endometrial cancer driven by PIK3CA mutation, TP53 loss, and MYC overexpression. This model enables systematic investigation of PI3Kα-targeted therapy resistance within an immunocompetent SEC context. Using this system, we identify FGFR signaling as a key driver of resistance to PI3Kα-targeted therapy and demonstrate that combined FGFR and PI3Kα inhibition, particularly when integrated with immune checkpoint blockade, elicits enhanced antitumor efficacy. Together, these findings establish a mechanistic and therapeutic framework for targeting FGFR-mediated resistance and immune evasion in SEC.

## Results

### Generation of a syngeneic orthotopic serous-like endometrial cancer model driven by the PIK3CA-H1047R mutation

**Fig. 1:**
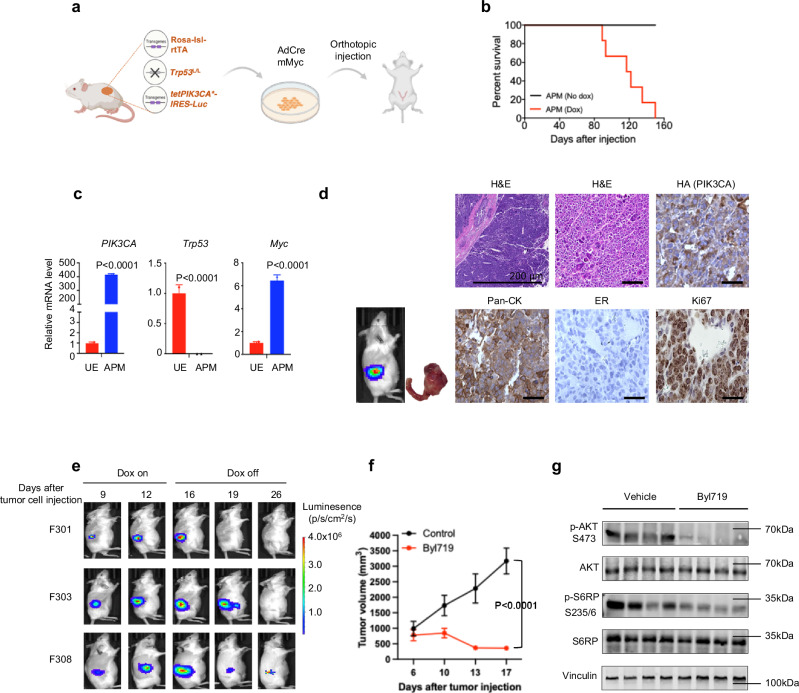
Characterization of the PIK3CA^H1047R^; Trp53^−/−^; Myc (APM) mouse model. **a** Schematic illustrating the strategy used to generate the APM transgenic model. Created in BioRender. Gu, H. (2026) https://BioRender.com/1czjz4t. **b** Kaplan-Meier survival curves comparing APM mice with doxycycline-naïve transgenic controls (*n* = 6 mice per group). Survival differences were analyzed using the two-sided log-rank (Mantel-Cox) test. **c** RT-qPCR analysis of PIK3CA^H1047R^ transgene, Trp53, and Myc expression in uterine epithelium (UE) and APM tumors. Data represent mean ± s.e.m.; *n* = 3 biologically independent tumors per group. Statistical significance was determined by two-tailed unpaired Student’s t-test. **d** Representative histology of primary APM tumors showing serous-like morphology (scale bar, 50 μm). Similar histological features were observed in *n* = 3 biologically independent tumors. **e** Representative longitudinal in vivo bioluminescence imaging of primary tumors during doxycycline treatment (Dox on) and following doxycycline withdrawal (Dox off) at the indicated time points after tumor cell injection (days 9, 12, 16, 19, and 26). Three representative mice (F301, F303, and F308) are shown. Luminescence is presented as photons per second per square centimeter (p/s/cm²). **f** Tumor volume changes in APM allografts treated with BYL719 (20 mg/kg) or vehicle. Data are presented as mean ± s.e.m.; *n* = 8 biologically independent tumors per group. Statistical significance between groups was determined by two-way ANOVA with multiple-comparison correction. **g** Immunoblot analysis of tumor lysates from mice treated with BYL719 or vehicle. Representative blots are shown; similar results were obtained in four biologically independent tumors per group. Source data are provided as a Source Data file.

To investigate the role of PI3K pathway activation in SEC development and resistance to PI3Kα inhibition, we generated a syngeneic orthotopic genetically engineered mouse model (GEMM) of SEC that combines oncogenic PIK3CA mutation, TP53 loss and MYC overexpression (termed APM), incorporating common genetic alterations observed in human SEC^3–5^. This APM model was initially established by isolating uterine epithelial cells from FVB mice carrying three genetic elements: Rosa-lsl-rtTA, floxed-Trp53, and a tetracycline (tet)-inducible PIK3CA^H1047R^-IRES-luciferase transgene^33^. These cells were then modified with adenoviral Cre and lentiviral mouse Myc before being orthotopically transplanted into the uterine wall of FVB female recipients (Fig. 1a). Following doxycycline administration, transplanted mice developed de novo primary APM uterine tumors within 3-5 months with full penetrance. In contrast, mice not treated with doxycycline remained tumor-free, demonstrating that PIK3CA-H1047R expression is required for tumor development (Fig. 1b). RT-qPCR and western blot analysis confirmed that doxycycline treatment significantly induced PIK3CA^H1047R^ expression and activated downstream PI3K signaling. While endogenous murine Pik3ca expression remained unaffected (Fig. 1c and Supplementary Fig. 1a, b). The tet-inducible PIK3CA-H1047R transgene was coupled to a luciferase reporter for in vivo monitoring of transgene expression (Supplementary Fig. 1c).

These primary tumors exhibited histological characteristics of SEC, including endometrial atrophy, papillary/micropapillary architecture with slit-like spaces, marked nuclear atypia, necrosis, and loss of TP53 expression. Tumor cells overexpressed PIK3CA and MYC, were estrogen receptor (ER)-negative, and co-expressed epithelial markers (Fig. 1d). We next benchmarked the APM model against a well-established PTEN-loss-driven EC GEMM, which has been extensively characterized across multiple studies and classified as a Type I-like, endometrioid EC^34–36^. RNA-seq-based gene set enrichment analysis (GSEA) revealed that APM tumors exhibited strong enrichment of G2M checkpoint activation, DNA replication stress-associated programs, E2F targets, and MYC target pathways relative to the PTEN-loss model^36^, consistent with molecular features reported for human SEC^4^ (Supplementary Fig. 1d). Copy-number variation (CNV) analysis further demonstrated extensive genomic instability in APM tumors, with a fraction of genome altered (FGA) of 0.33, comparable to human SEC tumors (median FGA = 0.41) and markedly higher than that observed in non-serous TCGA endometrial cancer (median FGA = 0.07) (Supplementary Fig. 1e).

To create a more time-efficient and experimentally tractable system for mechanistic and therapeutic studies, we then derived primary tumor cells from these de novo APM tumors and orthotopically injected them into the uterine wall of FVB female recipients. Upon doxycycline administration, tumors developed in all mice within one month, providing a reliable experimental model with predictable growth kinetics (Supplementary Fig. 1f). We assessed tumor dependency by withdrawing doxycycline from tumor-bearing mice, which resulted in rapid reduction of PIK3CA^H1047R^ expression and downstream signaling pathway activation (Fig. 1e and Supplementary Fig. 1g). Tumor cells underwent apoptosis within 24-48 hours, followed by tumor regression within one week (Supplementary Fig. 1h), indicating their dependence on PIK3CA-H1047R.

Finally, to assess pharmacologic inhibition, syngeneic transplants were treated with the PI3Kα-selective inhibitors BYL719 (alpelisib) and GDC-0077 (inavolisib). Consistent with genetic inactivation, both agents markedly suppressed tumor growth (Fig. 1f and Supplementary Fig. 1i, j) and reduced phosphorylation of AKT and S6RP (Fig. 1g), confirming that APM tumor growth is dependent on sustained PIK3CA-H1047R signaling.

### Tumors recurring after PIK3CA withdrawal exhibit FGFR-dependent proliferation

**Fig. 2:**
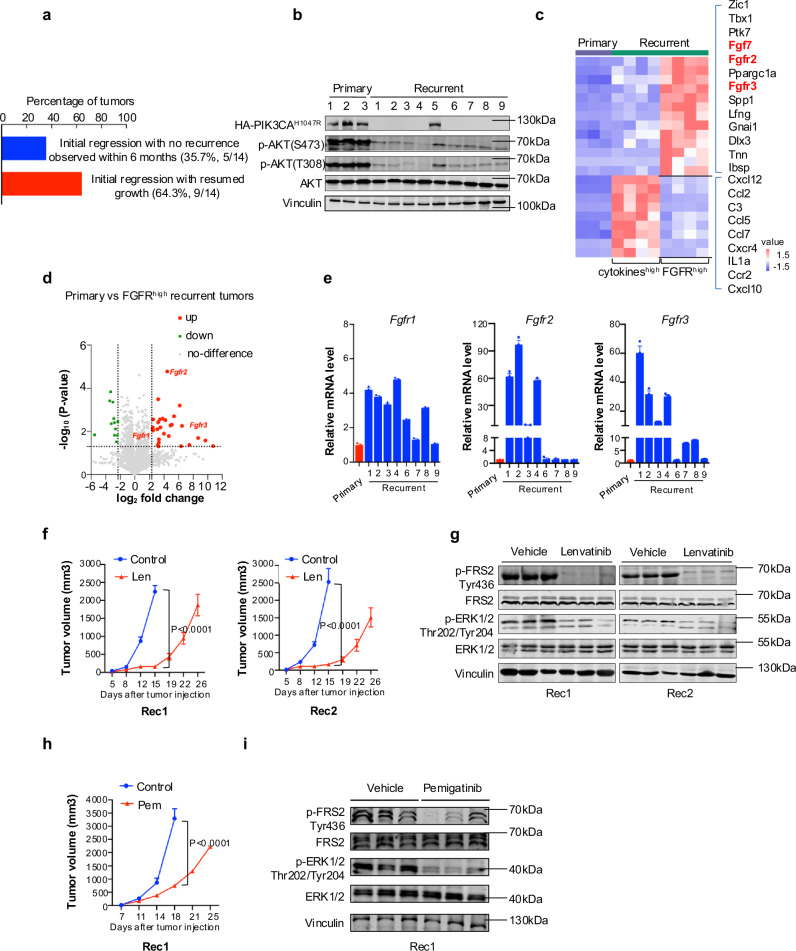
PIK3CA^H1047R^; Trp53^−/−^; Myc tumors recur after PIK3CA^H1047R^ withdrawal and exhibit FGFR-dependent proliferation. **a** Summary of primary tumor responses following doxycycline withdrawal: 5 out of 14 tumors (35.7%) regressed completely without regrowth (blue bar), whereas 9 out of 14 tumors (64.3%) initially regressed but subsequently regrew (red bar). **b** Immunoblot analysis showing expression levels of PIK3CA and associated downstream signaling pathways in primary and recurrent tumors. Representative blots are shown; similar results were obtained in three biologically independent primary tumors and nine biologically independent recurrent tumors. **c** Heat map illustrating significantly altered gene expression in recurrent tumors relative to primary tumors (Primary, *n* = 3; Recurrent, *n* = 8). Differential expression analysis was performed using bulk RNA-seq, and genes with adjusted *P* < 0.05 were considered significant. **d** Volcano plot illustrating significantly altered gene expression in FGFR^high^ recurrent tumors (*n* = 4 biologically independent tumors) relative to primary tumors (*n* = 3 biologically independent tumors). Differential expression analysis was performed on raw count data using a negative binomial generalized linear model. *P* values were calculated using a two-sided Wald test and adjusted for multiple comparisons using the Benjamini-Hochberg false discovery rate (FDR) method. Genes with FDR-adjusted *P* < 0.05 and |log₂ fold change | ≥ 2.32 were considered statistically significant. The horizontal dashed line indicates FDR-adjusted *P* = 0.05, and the vertical dashed lines indicate log₂ fold change thresholds of ±2.32. **e** FGFR transcript levels in primary and recurrent tumor-derived cell lines (*n* = 3 biologically independent primary tumor-derived cell lines and *n* = 8 biologically independent recurrent tumor-derived cell lines). qPCR measurements were performed in technical triplicate for each cell line, and the mean value per cell line was used for statistical analysis. Dots represent biologically independent cell lines. Data are presented as mean ± s.e.m. **f**, **h** Tumor growth curves of recurrent tumors Rec1 and Rec2 treated with lenvatinib or pemigatinib as indicated. Data are presented as mean ± s.e.m.; In **f**, *n* = 10 biologically independent tumors per group for Rec1 control, Rec1 lenvatinib, and Rec2 control; *n* = 8 for Rec2 lenvatinib. In **h**, *n* = 8 biologically independent tumors per group. Statistical significance was determined by two-way ANOVA with multiple-comparison correction. **g**, **i** Immunoblot analysis showing reduced phosphorylation of FRS2 and ERK1/2 after 12 days of treatment in Rec1 and Rec2 tumors. Experiments were independently performed in *n* = 3 biologically independent tumors per group with similar results. Source data are provided as a Source Data file.

To model tumor recurrence, which commonly arises from therapeutic resistance in a small population of tumor cells and can manifest as both local and distant relapse^37^, we withdrew doxycycline from a cohort of 14 tumor-bearing mice and monitored them for several months. About one-third (5/14) of tumors underwent complete regression within one month and did not recur (Fig. 2a), indicating that these tumors remained dependent on PIK3CA^H1047R^ signaling for their survival. In contrast, approximately two-thirds of tumors (9/14) initially regressed but later resumed growth despite the continued absence of doxycycline (Fig. 2a and Supplementary Fig. 2a). Since control mice without doxycycline induction never developed tumors, these recurrent tumors must have originated from resistant clones within the original tumors.

Recurrent tumors preserved serous-like histopathological features (Supplementary Fig. 2b and Supplementary Fig. 1e). Functionally, recurrent tumors exhibited markedly reduced PI3K pathway activity and resistance to BYL719, with the except of Rec5, which retained PIK3CA-H1047R expression and remained sensitive to BYL719 (Fig. 2b and Supplementary Fig. 2c, d). These findings demonstrate that although PIK3CA-H1047R is required for tumor formation, most tumors eventually acquire PIK3CA-H1047R- independent mechanisms to sustain growth.

Transcriptomic profiling further revealed distinct gene expression patterns between primary and recurrent tumors (Fig. 2c). Recurrent tumors clustered into two major subgroups: one characterized by high expression of FGF/FGFR family members (FGF7, FGFR1, FGFR2, FGFR3), and another defined by elevated expression of multiple cytokines (Fig. 2c–e and Supplementary Fig. 2e–g). Quantitative PCR analysis confirmed elevated expressions of FGFR1, FGFR2, and FGFR3 in FGFR-high recurrent tumors (Fig. 2e). A similar pattern was observed in tumors that acquired resistance to BYL719, which showed increased FGFR expression at the time of resistance (Supplementary Fig. 2h).

FGFR signaling has been implicated in therapy resistance and cancer progression across multiple tumor types^38,39^. To explore the functional relevance of elevated FGFR expression in recurrent tumors, we transplanted FGFR-high recurrent tumors into wild-type FVB mice and treated them with either lenvatinib or pemigatinib, a more FGFR-selective inhibitor approved for FGFR-driven cancers^40,41^. Both agents effectively suppressed tumor growth and were accompanied by reduced phosphorylation of fibroblast growth factor receptor substrate 2 (FRS2) and extracellular signal-regulated kinase (ERK) (Fig. 2f–i). Collectively, these results indicate that a subset of tumors recurring after PIK3CA^H1047R^ withdrawal becomes dependent on FGFR signaling for sustained proliferation.

### FGFR-driven resistance and clonal dynamics in recurrent tumors after PIK3CA-H1047R inhibition

**Fig. 3:**
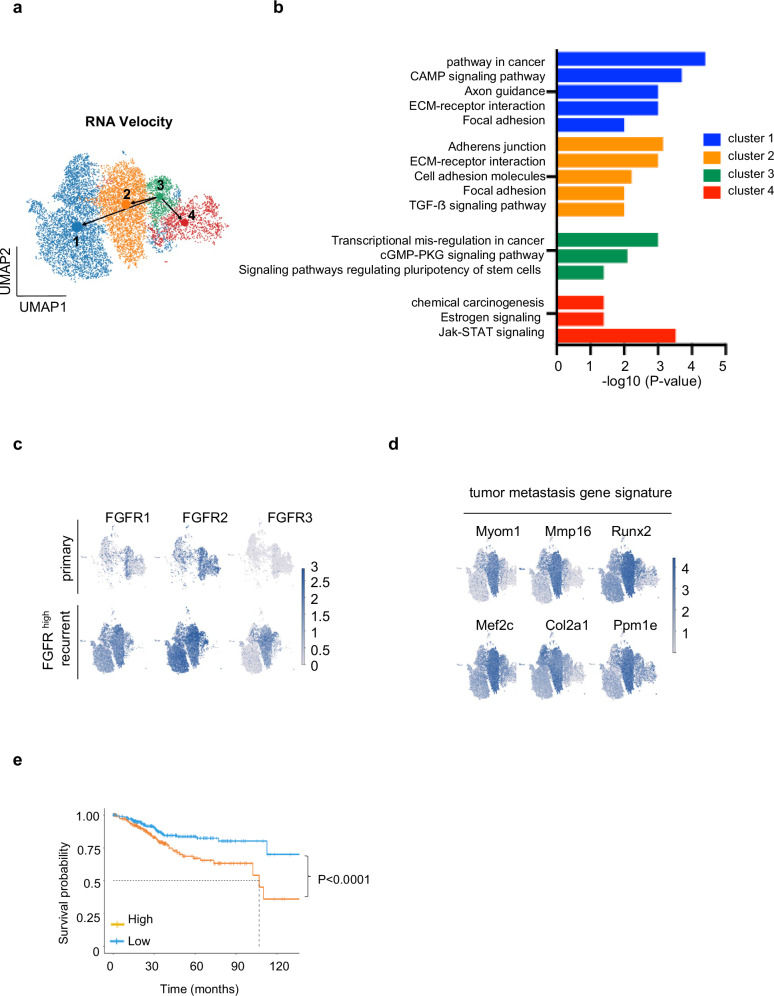
snRNA-seq reveals the evolutionary trajectory of FGFR^+^ tumor cells driving recurrence. **a** RNA velocity analysis overlaid on the UMAP projection of epithelial cells from primary and recurrent tumors (*n* = 3 biologically independent tumors per condition, pooled prior to sequencing). Velocity vectors suggest a directional transition from epithelial cluster 3 in primary tumors toward FGFR-high recurrent tumors. **b** KEGG pathway enrichment analysis of epithelial-cell DEGs identified from the snRNA-seq dataset. Differential expression was performed in Seurat (two-sided Wilcoxon rank-sum test) with Benjamini-Hochberg correction; genes with adjusted *P* < 0.05 were considered significant. KEGG over-representation analysis was conducted using clusterProfiler (one-sided hypergeometric test), and enrichment *P* values were adjusted for multiple comparisons using the Benjamini–Hochberg method. **c** UMAP feature plots showing that FGFR1- and FGFR2-high tumor cells are already present in primary tumors, whereas FGFR3 expression emerges predominantly in recurrent tumors, indicating intrinsic versus acquired resistance programs. **d**, **e** Genes enriched in epithelial cluster 2 (characterized by high FGFR1-3 expression) were associated with tumor metastasis signatures and reduced overall survival in TCGA UCEC patients (*n* = 546). Patients were dichotomized into high- and low-expression groups based on the median expression value. Overall survival was evaluated using Kaplan-Meier analysis, and statistical significance was assessed by a two-sided log-rank (Mantel-Cox) test. Source data are provided as a Source Data file.

To decipher the molecular programs and clonal dynamics driving residual and recurrent disease after PIK3CA-H1047R inhibition, we performed single-nucleus RNA sequencing (snRNA-seq) on primary tumors and FGFR-high recurrent tumors (analyzing 23,116 cells with a median of ~2,300 genes detected per cell) (Supplementary Fig. 3a). This analysis identified five major cell populations: epithelial cells, fibroblasts, endothelia cells, myeloid cells, and T cells (Supplementary Fig. 3b, c). RNA velocity analysis of epithelial cells further resolved four distinct epithelial clusters characterized by activation of unique signaling programs (Fig. 3a, b).

Detailed examination of FGFR1-3 expression patterns provided important insights into resistance mechanisms. Tumor cells with high FGFR1 and FGFR2 expression were already present in the primary tumors (Fig. 3c and Supplementary Fig. 2e, f), consistent with an intrinsic or de novo resistance mechanism. In contrast, FGFR3 expression emerged predominantly in the recurrent tumors, indicative of an acquired resistance mechanism (Fig. 3c and Supplementary Fig. 2e, f). Notably, epithelial cluster 2 in recurrent tumors showed coordinated enrichment of FGFR1-3 expression together with TGF-β signaling and cell adhesion pathways commonly associated with metastasis progression (Fig. 3a–d).

To assess the clinical relevance of these transcriptional programs, we derived a gene signature enriched in epithelial cluster 2. High expression of this FGFR-enriched signature correlated with increased expression of metastasis-associated gene programs and significantly reduced overall survival in patient datasets (Fig. 3e). These findings suggest that FGFR-high tumor cell populations represent both a prognostic marker and a potential therapeutic vulnerability.

To evaluate microenvironmental remodeling during tumor recurrence, we next compared immune compartments between primary and recurrent tumors. FGFR-high recurrent tumors (Rec1) exhibited an marked increase in M2-type TAMs, accompanied by a reduction in M1-TAMs and total T-cell infiltration relative to primary tumors (APM) (Supplementary Fig. 3d, e). Within the T-cell compartment, we observe a pronounced depletion of cytotoxic CD8⁺ T cells and a relative enrichment of exhausted T cells and regulatory T cells (Tregs), populations associated with immune suppression (Supplementary Fig. 3f). Together, these data define a coordinated immune remodeling program in FGFR-high recurrent tumors, characterized by M2-dominant TAMs, loss of cytotoxic CD8⁺ T cells, and expansion of Tregs, which collectively promote immune escape and tumor recurrence.

### FGFR signaling mediates resistance to PIK3CA-targeted therapy

**Fig. 4:**
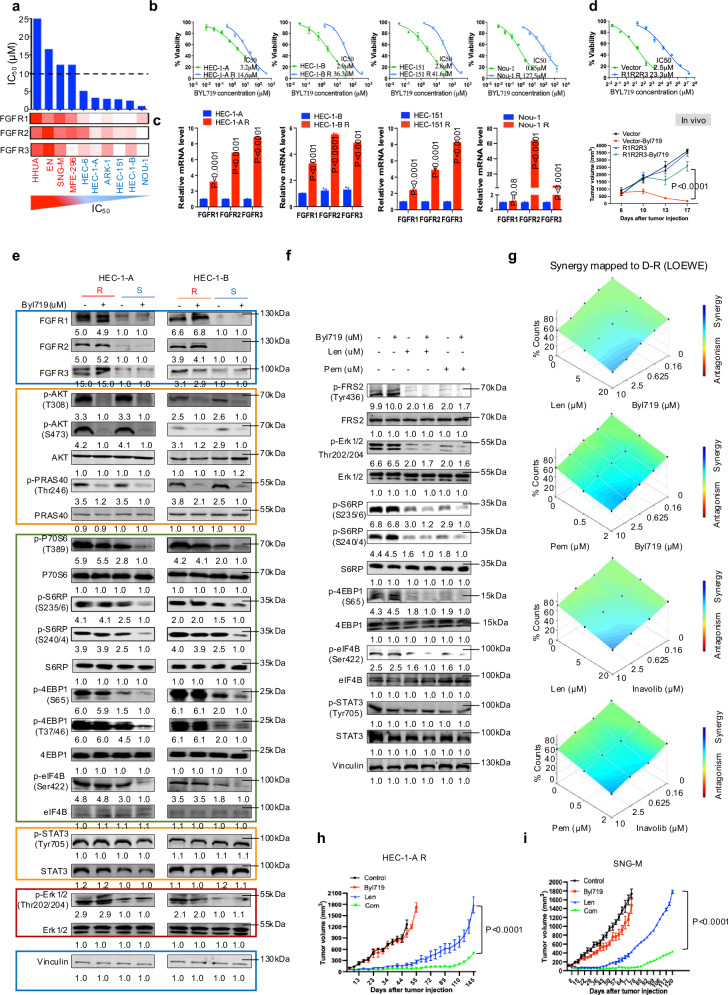
FGFR-driven MAPK-mTOR activation contributes to resistance to PI3Kα inhibition. **a** BYL719 IC₅₀ values across ten human endometrial cancer cell lines harboring PIK3CA mutations (top), together with relative FGFR1-3 expression levels (bottom). Cell lines are ordered according to increasing IC₅₀ values. IC₅₀ values were calculated by nonlinear regression from *n* = 3 independent biological experiments per cell line. **b** Dose-response analyses comparison proliferation of four BYL719-sensitive cell lines and their matched resistant counterparts following treatment with increasing doses of BYL719. Data are presented as mean ± s.e.m. from *n* = 3 independent biological experiments per cell line, each performed in technical triplicate. IC50 values were calculated using nonlinear regression. **c** Relative FGFR1-3 expression levels in BYL719-sensitive cell lines compared with their resistant derivatives. qPCR measurements were performed in triplicate for each cell line in *n* = 3 independent biological experiments. Data are presented as mean ± s.e.m. Statistical significance was determined by two-tailed unpaired Student’s t-test. **d** BYL719 sensitivity (IC_50_, top) and in vivo tumor growth (bottom) of primary tumor cells stably overexpressing FGFR1-3 or carrying an empty vector following BYL719 treatment. Top: Data are presented as mean ± s.e.m. from *n* = 3 independent biological experiments, each performed in technical triplicate. IC50 values were calculated using nonlinear regression. Bottom: Tumor growth curves are presented as mean ± s.e.m.; *n* = 10 tumors for Vector groups and *n* = 8 tumors for Vector+BYL719, R1R2R3 and R1R2R3 + BYL719 groups. Mice were randomly assigned to treatment groups. Statistical significance was determined by two-way ANOVA with multiple-comparison correction. **e** Immunoblot analysis of PI3K/AKT, MAPK, and STAT3 signaling in BYL719-sensitive (S) and -resistant (R) cells treated for 24 hours with 1 μM BYL719. Experiments were independently repeated three times with similar results. **f** Immunoblot analysis of BYL719-resistant cells treated with BYL719 (1 μM), lenvatinib (20 μM), pemigatinib (2 μM), or the indicated combinations, showing suppression of MAPK-mTOR signaling upon FGFR inhibition. Experiments were independently repeated three times with similar results. **g** Isobologram plots showing additive or synergistic effects of BYL719 or inavolisib combined with Lenvatinib or pemigatinib on HEC-1-A R cell viability, analyzed using the Combenefit synergy-antagonism model (LOEWE reference model). Experiments were independently repeated three times with similar results. Tumor growth curves of xenografts derived from BYL719-resistant HEC-1-A R (**h**) and intrinsically resistant SNG-M tumors (**i**) treated with the indicated agents. Tumor volumes are shown as mean ± s.e.m.; For HEC-1-A tumors, *n* = 4 per group for the control and BYL719 groups, and *n* = 6 per group for the lenvatinib and BYL719 + lenvatinib groups. For SNG-M tumors, *n* = 10 per group. Mice were randomly assigned to treatment groups. Statistical significance was determined by two-way ANOVA with multiple-comparison correction. Source data are provided as a Source Data file.

To further determine the role of FGFR signaling in resistance to PIK3CA-directed therapy, we treated a panel of ten PIK3CA-mutant human EC cell lines with the PI3Kα inhibitors BYL719 and GDC-0077^42–49^. Higher FGFR expression correlated with reduced sensitivity to both inhibitors, as reflected by increased IC_50_ values (0.9-27 μM for BYL719 and 0.6-20 μM for GDC-0077), with the exception of ARK1, which harbors HER2 amplification^23^ (Fig. 4a, Supplementary Fig. 4a and Supplementary Table 1).

To directly test whether FGFR contributes to acquired resistance, we selected four EC cell lines (HEC-1-A, HEC-1-B, HEC-151, and Nou-1) with low baseline FGFR expression and initial sensitivity to BYL719. Chronic exposure to increasing concentrations of BYL719 generated resistant derivatives, using 10 μM as the resistance threshold based on clinically achievable plasma levels^30^. All resistant lines displayed IC_50_ values greater than 10 μM and exhibited marked upregulation of FGFR1-3 compared with their parental counterparts (Fig. 4b, c). Similar results were obtained with GDC-0077 in HEC-1-A iR cells, an inavolisib-resistant derivative generated by stepwise dose escalation (Supplementary Fig. 4b). In addition, enforced expression of FGFR1-3 in primary APM tumor cells was sufficient to confer resistance to BYL719 (Fig. 4d).

Detailed signaling analyses revealed that although BYL719 effectively suppressed phosphorylation of AKT and its downstream target PRAS40 in both sensitive and resistant cells, phosphorylation of downstream mTOR effectors, including S6, eIF4B, and 4EBP1, persisted in BYL719-resistant HEC-1-A R and HEC-1-B R cells (Fig. 4e). Co-treatment with a PI3Kα inhibitor (BYL719 or GDC-0077) together with an FGFR inhibitor (lenvatinib or pemigatinib) effectively abolished phosphorylation of S6, eIF4B, and 4EBP1, leading to a marked reduction in proliferation in BYL719-resistant HEC-1-A R and SNG-M cells (Fig. 4f, g and Supplementary Fig. 4c). Similar effects were observed upon FGFR knockdown, which resensitized SNG-M and HEC-1-A R to BYL719 (Supplementary Fig. 4d).

Consistent with these findings, FGFR-high recurrent Rec1 tumors exhibited marked upregulation of FGFR1-3, accompanied by increased ERK1/2 phosphorylation and activation of mTOR downstream effectors (p-S6RP and p-4EBP1), relative to primary APM tumors, whereas STAT3 phosphorylation remained largely unchanged (Supplementary Fig. 4e). shRNA-mediated knockdown of FGFR1-3 in Rec1 tumors substantially reduced p-ERK1/2 and p-S6RP/p-4EBP1 levels, indicating that FGFR upregulation in the recurrent setting predominantly sustains MAPK-mTOR signaling (Supplementary Fig. 4e). Notably, AKT phosphorylation was robust in primary APM tumors but was not maintained in Rec1 tumors, consistent with loss of PIK3CA^H1047R^ activity. Thus, AKT is not the dominant downstream effector of FGFR-driven signaling in this model, reflecting an uncoupling of mTOR output from PI3K-AKT signaling in the resistant state. Accordingly, FGFR blockade in this context primarily suppresses the FRS2-MAPK-mTOR axis, with minimal effects on STAT3 and PI3K-AKT signaling (Supplementary Fig. 4f).

We next validated these findings in vivo. BYL719-resistant HEC-1-A R xenografts exhibited a markedly enhanced response to combined BYL719 and lenvatinib treatment compared with lenvatinib monotherapy (Fig. 4h and Supplementary Fig. 4g). This combination also demonstrated strong efficacy in intrinsically BYL719-resistant SNG-M xenografts (Fig. 4i and Supplementary Fig. 4h, i), with similar effects observed using the FGFR-selective inhibitor pemigatinib in combination with BYL719 (Supplementary Fig. 4j), indicating its potential to overcome both acquired and intrinsic resistance to PI3Kα inhibition. Similarly, in the APM model rendered resistant after prolonged BYL719 exposure (termed APM-R), FGFR inhibition restored sensitivity to PI3Kα blockade (Supplementary Fig. 4k). In contrast, in BYL719-sensitive HEC-1-A tumors, the combination produced a synergistic but less pronounced effect (Supplementary Fig. 4l, m). Collectively, these data indicate that PI3Kα and FGFR co-inhibition is at least additive and frequently synergistic across both PI3Kα-sensitive and -resistant settings, with synergy most evident in the resistant, FGFR-driven context.

### FGFR overexpression promotes tumor immune evasion in EC

**Fig. 5:**
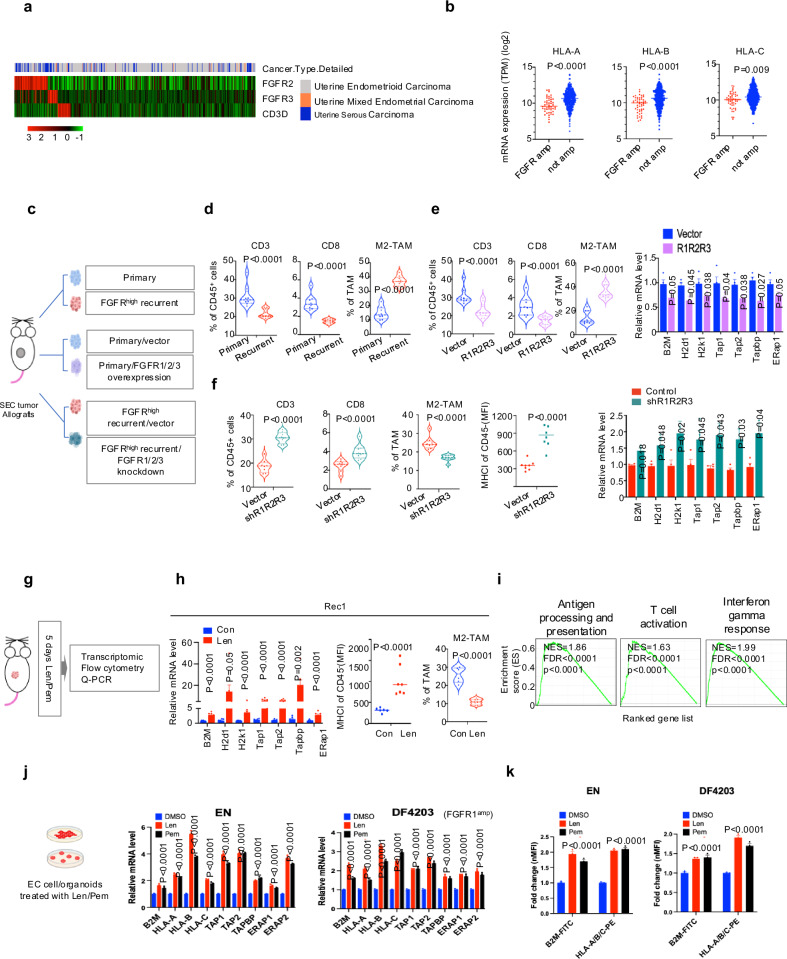
FGFR inhibition enhances antigen presentation and reduces M2-like tumor-associated macrophages. **a** TCGA analysis revealed an inverse relationship between FGFR expression and CD3D levels. RNA expression values (log2-transformed TPM) were obtained from the TCGA UCEC cohort (*n* = 527 tumors) and visualized using the R package pheatmap (https://www.cbioportal.org/study/summary?id=ucec_tcga_pan_can_atlas_2018). **b** Differential expression of HLA-A, HLA-B, and HLA-C in FGFR-amplified (*n* = 51) and non-amplified (*n* = 495) TCGA uterine endometrial carcinoma (UCEC) tumors. Each dot represents one independent tumor sample. RNA expression levels are shown as log₂-transformed TPM values. Horizontal lines indicate median values. Statistical significance was assessed using a two-sided Wilcoxon rank-sum (Mann-Whitney U) test. **c** Schematic of the in vivo experimental design. Created in BioRender. Gu, H. (2026) https://BioRender.com/956sotu. **d**–**f** Flow cytometry and RT-qPCR analyses of CD3^+^ and CD8^+^ T cells, M2-TAMs, and antigen-presentation gene expression in primary tumors, recurrent tumors, primary tumors overexpressing FGFR1/2/3, and recurrent tumors with FGFR knockdown. Each dot represents one biologically independent tumor. **d** Flow cytometric quantification of CD3⁺ cells, CD8⁺ cells, and M2-like TAMs in primary versus recurrent tumors; *n* = 8 biologically independent tumors for primary tumors and *n* = 10 for recurrent tumors. **e** Vector versus R1R2R3 tumors: flow cytometric analysis (left), *n* = 8 tumors for Vector and *n* = 10 tumors for R1R2R3; RT-qPCR analysis of antigen-presentation genes (right), *n* = 4 tumors per group. **f** Vector versus shR1R2R3 tumors: flow cytometric analysis (left), *n* = 8 tumors per group; RT-qPCR analysis of antigen-presentation genes (right), *n* = 4 tumors per group. Violin plots show the data distribution; the center line indicates the median. Data are presented as mean ± s.e.m. of biologically independent tumors. Statistical significance was determined by two-tailed unpaired Student’s t-test. **g**–**i** GSEA, RT-qPCR, and flow cytometry analyses demonstrating upregulation of antigen presentation-related genes and a reduction in M2-TAMs in FGFR-high recurrent Rec1 tumors following short-term lenvatinib treatment. **g** Schematic overview of experimental design. Created in BioRender. Gu, H. (2026) https://BioRender.com/ooq12da. **h** RT-qPCR analysis (left) of antigen-presentation genes in Rec1 tumors treated with vehicle control or lenvatinib; *n* = 6 tumors for the control group and *n* = 7 tumors for the lenvatinib treatment group. Data are presented as mean ± s.e.m. of biologically independent tumors. Statistical significance was determined by two-tailed unpaired Student’s t-test. Flow cytometric quantification of MHC I expression on CD45⁻ cells (middle) and M2-like TAMs (right); *n* = 6 tumors for the control group and *n* = 7 tumors for the lenvatinib treatment group. Each dot represents one independent tumor. Violin plots show the data distribution; the center line indicates the median. Statistical significance was determined by two-tailed unpaired Student’s t-test. **i** GSEA was performed using the GSEA software (v4.3.3, Broad Institute). Gene sets were evaluated using phenotype permutation testing. Normalized enrichment score (NES), nominal P values, and Benjamini-Hochberg FDR q values are shown. **j**, **k** Expression of antigen-presentation genes in EC cell lines and the DF4203 patient-derived organoid (PDO; FGFR1^amp^) following 7 days of FGFR inhibitor treatment. EN cells and DF4203 PDO were treated with lenvatinib (Len) or pemigatinib (Pem) as indicated. **j** RT-qPCR analysis of antigen-presentation genes in EN cells and DF4203 PDO. Experiments were independently repeated three times using the same cell line or PDO. For each independent experiment, RT-qPCR measurements were performed in technical triplicate. Data are presented as mean ± s.e.m. of *n* = 3 independent biological experiments. Statistical significance was determined by two-tailed unpaired Student’s t-test. **k** Flow cytometric analysis of surface B2M and HLA-A/B/C expression shown as fold change in mean fluorescence intensity (MFI). Experiments were independently repeated three times using the same cell line or PDO. Data represent mean ± s.e.m. from *n* = 3 independent experiments performed using the same cell line or PDO. Statistical significance was determined by two-tailed unpaired Student’s t-test. Illustration in **j** was created in BioRender. Gu, H. (2026) https://BioRender.com/uw4ak86. Source data are provided as a Source Data file.

Analysis of TCGA EC datasets revealed a significant inverse correlation between FGFR expression and CD3D levels, indicating reduced T-cell infiltration in FGFR-high tumors (Fig. 5a). Elevated FGFR2/3 expression was preferentially enriched in uterine serous carcinoma (Fig. 5a). Moreover, FGFR- amplified tumors (*n* = 51) exhibited significantly lower expression of major histocompatibility complex class I (MHC-I) genes (HLA-A, HLA-B, and HLA-C) compared with non-amplified tumors (*n* = 495) (Fig. 5b), consistent with impaired antigen presentation. CIBERSORT-based immune deconvolution further demonstrated that FGFR-high tumors contained fewer CD8⁺ T cells but a higher proportion of M2 macrophages, supporting a more immunosuppressive tumor microenvironment associated with FGFR activation (Supplementary Fig. 5a). Although changes in some immune subsets were less pronounced, FGFR-high tumors showed a general shift toward a less inflamed phenotype, with reduced effector T-cell signatures and a myeloid compartment skewed toward M2/TAM-like populations (Supplementary Fig. 5a).

Importantly, this immune landscape was recapitulated in our preclinical models (Fig. 5c). Both recurrent Rec1 tumors with spontaneous FGFR overexpression (Fig. 5d) and primary tumors engineered to overexpress FGFRs (Fig. 5e and Supplementary Fig. 5b) exhibited reduced T-cell infiltration, increased M2-type TAMs, and diminished MHC-I-mediated antigen processing and presentation. In contrast, tumors with shRNA-mediated FGFR knockdown displayed increased CD3⁺ and CD8⁺ T-cell infiltration, reduced M2 macrophages abundance, and enhanced MHC-I expression (Fig. 5f). Together, these findings indicate that elevated FGFR signaling drives an immunosuppressive tumor microenvironment in both human EC and the APM model, aligning the immune features of our preclinical system closely with those observed in patient tumors.

We next tested whether FGFR inhibition could reverse this immunosuppressive state. Using a FGFR-high serous-like EC allograft model, we performed immune and transcriptomic profiling following short-term (5 days) FGFR inhibitor treatment (Fig. 5g). FGFR blockade significantly increased the expression of MHC-I- associated and antigen-presentation genes and reduced M2-TAM populations (Fig. 5h and Supplementary Fig. 5c). GSEA of cancer- and immune-related genes revealed strong enrichment of antigen processing and presentation pathways in FGFR inhibitor-treated tumors (Fig. 5i), a pattern that was reproduced in additional FGFR-high recurrent Rec2 tumors (Supplementary Fig. 5d). Concordantly, human EC cell lines and patient-derived organoids (PDOs) harboring FGFR overexpression or amplification also exhibited enhanced antigen presentation following lenvatinib or pemigatinib treatment (Fig. 5j, k).

Collectively, these results support a model in which the primary immunomodulatory effects of lenvatinib in our system are mediated through tumor-cell-intrinsic FGFR blockade, rather than direct suppression of immune cells. Consistent with this, treatment of naïve, tumor-free mice with lenvatinib for 12 days did not alter the frequencies of M2-like macrophages or T cells in the spleen or peripheral blood (Supplementary Fig. 5e), arguing against a major direct systemic immune cell-intrinsic effect of the drug in the absence of tumor.

To define the downstream signaling mechanisms underlying FGFR-driven immune suppression, we next interrogated major pathways activated downstream of FGFR, including MAPK, STAT3, and PI3K-AKT signaling. Pharmacologic MAPK inhibition most closely recapitulated the immunomodulatory effects of FGFR blockade, increasing MHC-I expression on tumor cells both in vitro and in vivo Rec1 tumor allografts, while concomitantly reducing M2-type TAMs infiltration (Supplementary Fig. 5f, i). In contrast, inhibition of STAT3 or PI3K did not produce comparable changes in antigen-presentation gene expression (Supplementary Fig. 5g, h). Moreover, combined inhibition of MAPK and PI3K signaling markedly restored BYL719 sensitivity in PI3Kα-resistant APM-R tumors, which were generated by long-term BYL719 dose escalation and characterized by FGFR overexpression (Supplementary Fig. 2h). This combination led to robust tumor growth suppression in vivo (Supplementary Fig. 5j). Notably, mice maintained stable body weight during combined MAPK inhibitor and BYL719 treatment, indicating good tolerability (Supplementary Fig. 5j). Together, these findings identify MAPK-ERK signaling as a key downstream mediator of FGFR-driven immune suppression and therapeutic resistance, providing a mechanistic rationale for combination strategies integrating PI3Kα and MAPK pathway inhibition alongside FGFR-targeted therapy.

### FGFR inhibition enhances antitumor immunity and augments immunotherapy

**Fig. 6:**
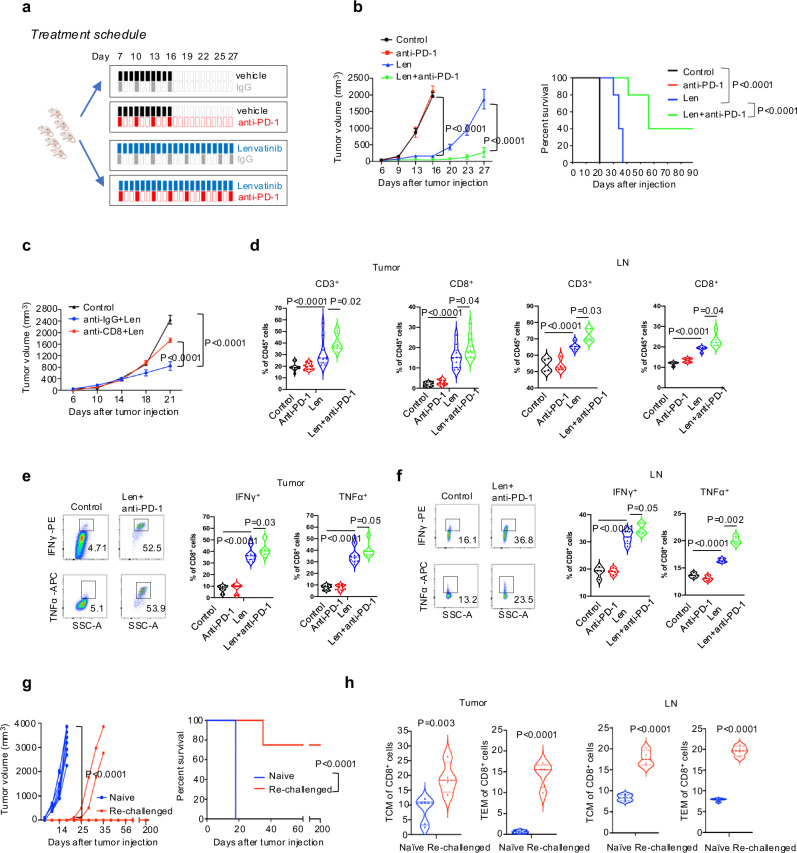
FGFR blockade enhances T-cell-mediated antitumor immunity and augments the therapeutic efficacy of PD-1 blockade. **a** Schematic of the experimental design for treating recurrent tumor allografts with the indicated agents for 21 days. Created in BioRender. Gu, H. (2026) https://BioRender.com/mitdqop. **b** Tumor volume curves (left) and Kaplan-Meier survival analysis (right) of recurrent Rec1 tumors treated with lenvatinib alone or in combination with anti-PD-1 therapy. Tumor volumes are shown as mean ± s.e.m., *n* = 10 tumors for the control, lenvatinib, and lenvatinib + anti-PD-1 groups, and *n* = 8 tumors for the anti–PD-1 group. Statistical significance for tumor growth was determined by two-way ANOVA with multiple-comparison correction. Survival analysis was performed using the two-sided log-rank (Mantel-Cox) test (*n* = 5 mice per group). **c** Tumor growth curves of mice treated with lenvatinib in the presence or absence of an anti-CD8 neutralizing antibody; tumor volumes are presented as mean ± s.e.m.; *n* = 6 tumors per group. Mice were randomly assigned to treatment groups. Statistical significance was determined by two-way ANOVA with multiple-comparison correction. **d**–**f** Flow cytometric analysis of intratumoral and draining lymph node (dLNs) CD3+ and CD8 + T-cell populations and effector cytokine production following treatment with the indicated agents (*n* = 10 tumors per group and *n* = 5 dLNs per group). Each dot represents one biologically independent tumor or dLN. Violin plots depict the data distribution; the center line indicates the median. Statistical significance was determined by two-tailed unpaired Student’s t-test. **g** Tumor growth curves and survival analysis of recurrent tumors transplanted into Naïve mice or mice rechallenged after complete tumor regression. Tumor growth is shown as mean ± s.e.m., *n* = 8 tumors per group. Mice were randomly assigned to experimental groups. Statistical significance for tumor growth was determined by two-way ANOVA with multiple-comparison correction. Survival was analyzed using the two-sided log-rank (Mantel-Cox) test (*n* = 5 mice per group). **h** Six days after recurrent tumor injection into Naïve or rechallenged mice, tumors and dLNs were harvested for memory T cell analysis (*n* = 6 tumors for naïve and *n* = 5 tumors for re-challenged; *n* = 5 dLNs per group). Flow cytometry was used to quantify CD8 TEM (CD45^+^ CD3^+^ CD8^+^ FOXP3^-^ CD62L^-^ CD44^+^ KLRG1^+^) and CD8 TCM (CD45^+^ CD3^+^ CD8^+^ FOXP3^-^ CD62L^+^ CD44^+^ KLRG1^-^). Each dot represents one biologically independent tumor. Violin plots depict the data distribution; the center line indicates the median. Statistical significance was determined by two-tailed unpaired Student’s t-test. Source data are provided as a Source Data file.

The observed increase in tumor antigen presentation and enhancement of antitumor T cell response suggested that combining FGFR inhibition with PD-1 blockade might further improve therapeutic efficacy. To test this hypothesis, we treated mice bearing FGFR-high recurrent tumors with lenvatinib alone or in combination with an anti-PD-1 antibody (Fig. 6a). Lenvatinib monotherapy produced transient tumor control followed by regrowth, whereas the combination led to markedly stronger and more durable tumor suppression (approximately 85% reduction in tumor volume by day 27; Fig. 6b), with comparable effects observed in additional FGFR-overexpressing recurrent tumors and in models treated with other FGFR inhibitors (Supplementary Fig. 6a, b).

Depletion of CD8^+^ T cells in immunocompetent FVB mice substantially attenuated the anti-tumor activity of lenvatinib (Fig. 6c and Supplementary Fig. 6c), indicating that CD8⁺ T cells are required for lenvatinib-mediated tumor control. Immunological profiling further revealed that lenvatinib monotherapy increased the production of effector cytokines (e.g., IFN-γ and TNF-α) by CD8^+^ T cells, an effect that was further enhanced by combination with anti-PD-1 therapy (Fig. 6d–f and Supplementary Fig. 6d, e), with similar results observed in tumors treated with other FGFR inhibitors (Supplementary Fig. 6f, g).

Notably, combined lenvatinib and anti-PD-1 therapy achieved complete tumor regression in ~40% of mice (Fig. 6b). To assess long-term immunity, mice that achieved complete responses were re-challenged with tumor cells four weeks after treatment cessation. Whereas all naive control mice developed tumors within one month, 80% of re-challenged mice remained tumor-free (Fig. 6g), consistent with the establishment of durable immune protection. Mechanistically, analysis performed six days after tumor re-inoculation revealed significantly increased frequencies of effector memory (TEM) and central memory (TCM) T cells in both tumors and draining lymph nodes compared with naive mice (Fig. 6h). Together, these findings demonstrate that FGFR inhibition combined with PD-1 blockade not only enhances immediate antitumor immunity but also induces durable immunological memory capable of preventing tumor recurrence.

## Discussion

SEC remains one of the most aggressive EC subtypes, yet progress in understanding its molecular pathogenesis and therapeutic vulnerabilities has been hindered by the lack of in vivo models that faithfully reproduce its defining biological features. SEC is characterized by extensive copy-number alterations, genomic instability, hallmark serous histology, and distinctive immune interactions, attributes largely absent from existing endometrioid-based models^50^. To address this gap, we generated a clinically relevant, immunocompetent SEC-like mouse model - termed APM - by integrating oncogenic PIK3CA mutation, Trp53 loss, and Myc overexpression, three genetic alterations that collectively define high-risk serous tumors^4^. This APM model system faithfully recapitulates key molecular hallmarks of human SEC, including a CNV-high genomic profile, enrichment of G2M checkpoint activation, replication stress programs, E2F and MYC transcriptional signatures, and classic serous morphology with high-grade architectural and nuclear atypia. Because APM tumors and their derived recurrent tumors evolve under selective pressures imposed by genomic instability, replication-associated stress, and immune surveillance, this system provides a unique opportunity to interrogate therapeutic pressure-driven clonal evolution while preserving intact tumor-immune interactions.

By integrating this model with analyses of EC cell lines, xenografts, and patient datasets, we identified FGFR signaling as a central determinant of resistance to PI3Kα-targeted therapy. Single-cell transcriptomic profiling reveals a functional hierarchy within the FGFR family: FGFR1 and FGFR2 are predominantly associated with intrinsic resistance, whereas FGFR3 becomes selectively enriched during acquired resistance, consistent with adaptive signaling rewiring under therapeutic pressure. Mechanistically, FGFR activation sustains mTORC1 activity and enhances MAPK signaling despite effective PI3Kα blockade, as evidenced by persistent phosphorylation of S6 and 4EBP1 together with increased ERK phosphorylation, while AKT and STAT3 activity remain largely unchanged. These findings position persistent MAPK-mTORC1 signaling downstream of FGFR as the dominant proliferative driver enabling escape from PI3Kα inhibition. Accordingly, dual PI3Kα and FGFR inhibition produced markedly enhanced antitumor effects in vivo, validating FGFR activation as a therapeutically actionable resistance node.

These mechanistic observations align with extensive preclinical evidence demonstrating that FGFR-mediated pathway rewiring enables tumor cells to escape PI3K or receptor tyrosine kinase (RTK) inhibition^39,51–53^, and that dual PI3K-FGFR blockade yields robust synergy across multiple cancer types^54,55^. Early-phase clinical trials evaluating alpelisib in combination with the FGFR1-3 inhibitor infigratinib further underscore the translational potential of this strategy, although overlapping toxicities, such as hyperglycemia and hyperphosphatemia, emphasize the importance of optimized dosing regimens and biomarker-guided patient selection^56^. Together with our mechanistic data, these findings provide strong biological and translational justification for pursuing PI3K-FGFR combination therapies in molecularly defined subsets of SEC.

Beyond tumor-intrinsic signaling, our data demonstrate that FGFR activation also orchestrates immune evasion. Elevated FGFR expression suppresses tumor immunogenicity by downregulating MHC-I/HLA-mediated antigen processing and presentation and by promoting an immunosuppressive tumor microenvironment characterized by increased infiltration of M2-type TAMs. Tumor-cell-specific FGFR depletion phenocopied the immunologic impact of systemic FGFR inhibition, confirming that these effects arise from tumor-intrinsic FGFR signaling rather than off-target effects on immune cells. Importantly, FGFR inhibition restored antigen presentation, enhanced CD8⁺ T-cell activation, improved responsiveness to PD-1 blockade, and promoted durable immunologic memory. These findings support a conceptual model in which FGFR signaling functions as a lineage-specific immune checkpoint that lowers tumor antigenicity and conditions an immunosuppressive microenvironment.

Our observations integrate into a broader mechanistic and clinical framework supporting FGFR-immune checkpoint blockade (ICB) combination strategies. In FGFR3-mutant bladder cancer, FGFR inhibition enhances anti-PD-1 efficacy by increasing CD8⁺ T-cell infiltration and restraining regulatory T-cell expansion^57^. Parallel immune-activating effects of FGFR blockade have been reported in hepatocellular, gastric, and lung cancers, where FGFR inhibition increases IFNγ production, boosts MHC-I expression, and strengthens T-cell activation^58,59^. Clinically, the combination of lenvatinib and pembrolizumab has produced substantial and clinically meaningful response rates and survival benefits in advanced EC^14^, while early-phase trials combining the FGFR4 inhibitor FGF401 with spartalizumab have demonstrated manageable safety profiles and preliminary efficacy across solid tumors^60^.

However, FGFR pathway activation alone is not sufficient to uniformly predict benefit from FGFR-targeted immunotherapy. Although lenvatinib plus PD-1 blockade has shown substantial activity, responses remain highly heterogeneous, and FGFR-high status has not consistently emerged as a predictive biomarker. Our study provides mechanistic insight into this heterogeneity by revealing the emergence of two distinct recurrent tumor states following PIK3CA withdrawal. One state is characterized by FGFR upregulation coupled to immune suppression mediated through impaired antigen presentation and macrophage polarization. Tumors within this state derive substantial benefit from combined FGFR inhibition and PD-1 blockade, consistent with FGFR functioning as a lineage-specific immune checkpoint in this context.

In contrast, we identified a subset of recurrent tumors characterized by increased cytokine expression in the absence of FGFR amplification or overexpression. These tumors exhibit features of an FGFR-independent immune-resistant program and appear to evade immune surveillance through inflammation-linked remodeling of the tumor microenvironment. Sustained cytokine signaling has been associated with immune dysfunction, including impaired antigen presentation, cytokine-driven myeloid cell infiltration, and the development of T-cell exhaustion, collectively promoting adaptive immune resistance^61–63^. Accordingly, cytokine-high recurrent tumors may be less amenable to immunotherapeutic strategies that rely on reinvigoration of cytotoxic T-cell responses, highlighting a potential limitation of immune checkpoint blockade in this subset. This alternative resistance state is not explained by FGFR-mediated immune suppression and provides a mechanistic framework for the observed heterogeneity in responses to FGFR-immune checkpoint blockade combinations, underscoring the need for biomarker frameworks that integrate inflammatory programs in addition to FGFR activity.

In conclusion, our study establishes a dual role for FGFR signaling in mediating both therapy resistance and immune evasion in SEC. By demonstrating that FGFR inhibition augments the efficacy of both PI3Kα-targeted therapy and immune checkpoint blockade, we provide a compelling rationale for therapeutically integrating FGFR blockade into combination regimens (Supplementary Fig. 6h). At the same time, overlapping toxicities, particularly in PI3K-FGFR combinations, remain a major clinical challenge, requiring careful dose optimization, scheduling, and supportive management. Ultimately, realizing the full clinical potential of these approaches will depend on biomarker-guided patient stratification, precision dosing paradigms, and rational combination designs that maximize antitumor potency while maintaining tolerability.

## Methods

### Mice

All animal experiments were performed in accordance with protocols (03-111, 06-034, 02-127) approved by the Dana-Farber Cancer Institute (DFCI) Institutional Animal Care and Use Committee (IACUC). Mice were housed under specific-pathogen-free conditions in a temperature- and humidity-controlled facility on a 12-hour light/12-hour dark cycle, with ambient temperature maintained at 22 ± 2 °C and relative humidity at 40–60%. Food and water were provided ad libitum. The TetO-PIK3CA^H1047R^ transgenic mouse line was maintained in our laboratory as previously described^33^. The Tp53^loxP/loxP^ mouse line (FVB.129P2-Trp53tm1Brn/Nci, #01XC2) was obtained from the National Cancer Institute Mouse Repository. Both strains were backcrossed for more than ten generations onto the FVB/N background before being intercrossed to generate homozygous lines. Female FVB/NJ and athymic nude mice (6 weeks of age) were obtained from The Jackson Laboratory.

### Cell culture

The following human EC cell lines were used in this study: HEC-1-A (ATCC, HTB-112) and HEC-1-B (ATCC, HTB-113); MFE-296 (Sigma Aldrich, 98031101-1VL); ARK1 (courtesy of Dr. Dipanjan Chowdhury, Dana-farber Cancer Institute); HEC-151, HEC-6, and HHUA (kindly provided by Dr. Jessie A. Sanai, Memorial Sloan Kettering Cancer Center); and EN, NOU-1, and SNG-M (generously provided by Dr. Gordon B. Mills, MD Anderson Cancer Center). All cell lines were routinely tested for mycoplasma contamination and authenticated by short tandem repeat (STR) profiling.

Primary mouse tumor-derived cells were established from freshly isolated primary or recurrent uterine tumors. Tumors were minced and enzymatically digested to generate single-cell suspensions, which were cultivated in serum-free F-Medium (Ham’s F-12:DMEM, 1:3) supplemented with 25 ng/mL hydrocortisone, 5 μg/mL insulin, 8.5 ng/mL cholera toxin, 0.125 ng/mL epidermal growth factor (EGF), 100 μg/mL penicillin-streptomycin, and 5 μM Y-27632 (ROCK1 inhibitor). Following initial tumor cell selection, cultures were maintained in F-medium supplemented with 1% fetal bovine serum (FBS).

### Generation of PI3Kα inhibitor-resistant cell lines

To generate BYL719-resistant cell lines, HEC-1-A, HEC-1-B, HEC-151, and NOU-1 cells were continuously exposed to increasing concentrations of BYL719, starting at 1ug/ml. Cells were serially passaged with stepwise dose escalation until resistant populations proliferated at rates comparable to parental controls in the presence of 50 μg/mL BYL719. This process required approximately six months and yielded stable BYL719-resistant derivatives.

Similarly, a GDC-0077-resistant HEC-1-A line (HEC-1-A iR) was generated through gradual dose escalation of GDC-0077. Acquisition of resistance was confirmed by drug withdrawal for 48 hours, followed by comparison of dose-response profiles between resistant and parental cells, demonstrating markedly reduced sensitivity to the corresponding PI3Kα inhibitor.

### Tumor growth and treatment

Primary and recurrent tumor cells (5 × 10^5^- 2×10^6^) for orthotopic implantation were suspended in serum-free DMEM mixed with 40% Matrigel and injected into 6-8-week-old female mice. For tumor-infiltrating immune cell profiling, 5×10^6^ recurrent tumor cells were implanted. Tumor size was measured 2-3 times per week, and the tumor volume was calculated using a modified ellipsoid formula (0.5 × length × width^2^). Mice bearing APM tumors received doxycycline-containing chow (2000 ppm) throughout the experiment. Mice were euthanized by CO2 inhalation when tumor volumes met humane endpoints described in the IACUC protocols or upon severe health deterioration. Tumor burden was monitored by volume, and all efforts were made to maintain tumor size within acceptable limits (generally not exceeding ~4000 mm³) in accordance with the relevant animal protocols.

For therapeutic studies, BYL719 and lenvatinib were dissolved in 0.5% methylcellulose and administered orally once daily at doses of 20 mg/kg and 10 mg/kg, respectively. Pemigatinib and trametinib were dissolved in 10% DMSO/90% corn oil and administered orally once daily at 1 mg/kg. GDC-0077 was formulated in 10% DMSO, 40% PEG300, 5% Tween-80, and 45% saline, and administered by oral gavage once daily at 50 mg/kg. Anti-PD-1 antibody (clone, 332.8H3, mouse IgG1) was diluted in PBS and administered intraperitoneally at 200 μg per mouse every 3 days^64^. Control IgG (RTK2758, Biolegend) and anti-CD8 antibody (clone 53.6.7, Biolegend) were diluted in PBS and administered intravenously at 200 μg per mouse once weekly. Animals were monitored daily for clinical signs of toxicity, including activity, appetite, grooming, hydration status, and body weight.

### Bioluminescence Imaging

Mice were administered D-luciferin (Gold Biotechnology) via intraperitoneal injection at a dose of 120 mg/kg. Ten minutes after injection, bioluminescent signals were acquired using an IVIS Spectrum imaging system (PerkinElmer). Images were processed and quantified using Living Image software (PerkinElmer).

### Tissue digestion

To generate single-cell suspensions, tumors were excised, minced and enzymatically dissociated in a collagenase/hyaluronidase buffer (DMEM supplemented with 5% FBS, 10 mM HEPES, 100 μg/mL penicillin-streptomycin, 20 μg/mL DNase I, and 1× collagenase/hyaluronidase). Tissues were incubated at 37 °C for 40-60 minutes with gentle agitation. Following digestion, samples were treated with ammonium-chloride-potassium (ACK) lysis buffer to remove red blood cells (RBCs) and then filtered through a 70 μm cell strainer to eliminate debris and undigested material.

Tumor-draining lymph nodes (TDLNs) were mechanically dissociated by gently passing the tissues through a 70 μm cell strainer using the plunger of a 5 mL syringe, followed by ACK lysis as described above.

### Cell viability assay

Tumor cells were seeded in 96-well plates at a density of 2500 cells per well and allowed to adhere overnight. Cells were then treated with the indicated drugs at varying concentrations for 72 hours. Cell viability was assessed using the CellTiter-Glo 2.0 assay (Promega, #G9242) according to the manufacturer’s instructions. Luminescence was measured and normalized to untreated controls, which were defined as 100% viability. Growth inhibition was calculated based on relative luminescence values. Dose-response curves were generated using nonlinear regression analysis in GraphPad Prism 8.2, and IC_50_ values were derived from the fitted curves.

### Generation of FGFR-deficient and FGFR-overexpressing cells

Five independent shRNA constructs targeting human FGFR1, FGFR2, and FGFR3, as well as five constructs targeting mouse Fgfr1, Fgfr2, and Fgfr3, were obtained from the Sigma Mission shRNA library. All shRNA candidates were screened across multiple tumor cell lines to evaluate knockdown efficiency. For each FGFR gene, the shRNA achieving the most robust and reproducible knockdown was selected for subsequent studies: human FGFR1 (TRCN0000121106), FGFR2 (TRCN0000000373), FGFR3 (TRCN0000231051); mouse Fgfr1 (TRCN0000023295), Fgfr2 (TRCN0000023715), and Fgfr3 (TRCN0000361155).

Lentiviral particles containing the selected shRNAs were produced and used to infect target cells, followed by selection with puromycin (1 μg/mL) to generate stable knockdown cell lines. Knockdown efficiency was confirmed by RT-qPCR and Western blotting analysis.

To generate FGFR-overexpressing cells, full-length coding sequences of mouse Fgfr1, Fgfr2, and Fgfr3 were amplified from cDNA and individually cloned into the pCDH-CMV-EF1-Puro lentiviral expression vector. All constructs were verified by Sanger sequencing. Lentiviral particles were produced using standard packaging plasmids in HEK293T cells and used to transduce tumor cells. Stable integrants were selected with puromycin (1 µg/ml). Overexpression levels were validated using RT-qPCR and Western blotting. Newly generated plasmids are available from the corresponding author upon reasonable request.

### Immunohistochemistry

Tumor specimens were fixed overnight in 10% neutral-buffered formalin and subsequently transferred to 70% ethanol. Tissue processing, paraffin embedding, sectioning, and hematoxylin and eosin (H&E) staining were performed by the Harvard Rodent Histopathology Core. Histological evaluation of primary and recurrent tumors was conducted by independent pathologists at Harvard Medical School.

Immunohistochemical (IHC) was performed using standard protocols. The following primary antibodies were used: anti-HA (1:400, Cell Signaling Technology, #3724), anti-pan- cytokeratin (1:200, Abcam, #ab7753), anti-ERα (1:200, Thermo Fisher Scientific, #RM9101S0), anti-Ki67 (1:500, Abcam, #ab15580), anti-cleaved caspase-3 (1:400, Cell Signaling Technology, #9661), anti-FGFR1 antibody (1:50, Abcam, #ab76464), anti-FGFR2 antibody (1:50, Abcam, #ab106648), and anti-FGFR3 antibody (1:50, Abcam, #ab180906).

### RNA extraction and reverse transcription-quantitative PCR (RT-qPCR)

Total RNA was isolated using the RNeasy Plus Mini Kit (QIAGEN, #74134) according to the manufacturer’s instructions. cDNA was synthesized from 2 µg of RNA using the iScript Reverse Transcription Supermix (Bio-Rad, #1708841). Quantitative PCR was performed using a real-time PCR system following standard protocols. Gene expression levels were normalized to endogenous reference genes and calculated using the comparative Ct method.

Primer sequences used for RT-qPCR are provided in Supplementary Table 2.

### Western blotting

Western blotting was performed using standard protocols. Primary antibodies used were as follows: HA (1:1000, 3F10, Roche), phospho-S6 ribosomal protein (Ser235/236, 1:1000, Cell Signaling Technology, #2211), phospho-S6 ribosomal protein (Ser240/244, 1:1000, Cell Signaling Technology, #2215), total S6 ribosomal protein (1:1000, Cell Signaling Technology, #2217), MYC (1:1000, Santa Cruz Biotechnology, sc-40), phospho-AKT (Ser473, 1:1000, Cell Signaling Technology, #4060), phospho-AKT (Thr308, 1:1000, Cell Signaling Technology, #2965), total AKT (1:1000, Cell Signaling Technology, #9272), phospho-FRS2 (Tyr436, 1:500, Cell Signaling Technology, #3861), total FRS2 (1:1000, R&D Systems, MAB4069), phospho-ERK1/2 (Thr202/Tyr204, 1:1000, Cell Signaling Technology, #9101), total ERK1/2 (1:1000, Cell Signaling Technology, #9102), phospho-PRAS40 (1:1000, Cell Signaling Technology, #2997), total PRAS40 (1:1000, Cell Signaling Technology, #2691), phospho-p70S6 (1:1000, Cell Signaling Technology, #9205), total p70S6 (1:1000, Cell Signaling Technology, #9202), FGFR1 (1:500, Cell Signaling Technology, #9740), FGFR2 (1:500, Cell Signaling Technology, #23328), FGFR3 (1:500, Cell Signaling Technology, #4574), phospho-4EBP1(Ser65, 1:1000, Cell Signaling Technology, #9451), phospho-4EBP1 (Thr37/46, 1:1000, Cell Signaling Technology, #2855), total 4EBP1 (1:1000, Cell Signaling Technology, #9452), phospho-eIF4B (Ser422, 1:1000, Cell Signaling Technology, #3591), total eIF4B (1:1000, Cell Signaling Technology, #3592), phospho-STAT3 (Tyr705, 1:1000, Cell Signaling Technology, #9145), total STAT3 (1:1000, Cell Signaling Technology, #9139), and vinculin (1:5000, Sigma -Aldrich, V9131). Uncropped and unprocessed full scans of the blots are provided in the Source Data file.

### Flow cytometry

Single-cell suspensions were prepared as described in the “Tissue Digestion” section. Cells were stained with LIVE/DEAD Fixable Aqua Dead Cell Stain (Thermo Fisher Scientific) in cold FACS buffer (PBS supplemented with 0.2% BSA and 5 mM EDTA) for 10 min on ice. Cells were then incubated with anti-mouse CD16/32 (1:50, clone 93, BioLegend) for 15 min on ice to block Fc receptors. Following Fc blocking, cells were stained with fluorophore-conjugated antibodies diluted in FACS buffer for 30 min on ice.

The following antibodies were use: CD45 (1:100, 30-F11, BioLegend), CD3 (1:100, 145-2C11, BioLegend), CD4 (1:100, RM4-5, BioLegend), CD8A (1:100, 53-6.7, BioLegend), CD44 (1:100, IM7, BioLegend), CD62L (1:100, MEL-14, BioLegend), FOXP3 (1:100, MF-14, BioLegend), IFN-γ (1:100, XMG1.2, BioLegend), TNF-α (1:100, MP6-XT22, BioLegend), KLRG1 (1:100, 2F1/KLRG1, BioLegend), H2-K^q^ (1:00, KH114, BioLegend), HLA-A/B/C (1:100, W6/32, BioLegend), β2-microglobulin (1:100, A17082A, BioLegend), CD11b (1:100, M1/70, BioLegend), F4/80 (1:100, BM8, BioLegend), CD206 (1:100, MMR, BioLegend), FGFR1 (1:50, M19B2, Thermo Fisher Scientific), FGFR2 (1:50, SP273, abcam), and FGFR3 (1:50, 2H10B4, Thermo Fisher Scientific).

For detection of unconjugated primary antibodies, cross-adsorbed goat anti-rabbit IgG (H + L) and goat anti-mouse IgG (H + L) secondary antibodies (1:5000; Life Technologies, #A11008 and #A21235) were used.

For intracellular staining, cells were fixed and permeabilized using the Foxp3/Transcription Factor Staining Buffer Set (eBioscience, #00-5523-00) according to the manufacturer’s instructions. For intracellular cytokine analysis, cells were stimulated with Leukocyte Activation Cocktail containing the protein transport inhibitor Brefeldin A (BD Biosciences, # 550583) at 37 °C for 4 hours prior to staining.

Flow cytometry data were acquired on an LSR Fortessa HTS analyzer (BD Biosciences) using BD FACSDiva software and analyzed with FlowJo (BD Biosciences). Gating strategies are shown in Supplementary Fig. 7.

### Transcriptome analysis

Transcriptomic profiling was performed using a custom Ion AmpliSeq panel comprising 4,604 cancer- and immune-associated genes, designed with the Ion AmpliSeq Designer (Thermo Fisher Scientific) as previously described. For each sample, 10 ng of total RNA was used for cDNA library preparation. Libraries were multiplexed, amplified using the Ion OneTouch™ 2 System, and sequenced on an Ion Torrent Proton™ System (Thermo Fisher Scientific). Raw sequencing data were processed using Torrent Suite software and the AmpliSeqRNA analysis plugin (Thermo Fisher Scientific) to generate gene-level count data.

For downstream analyses, including Gene Set Enrichment Analysis (GSEA) and pathway enrichment analysis based on the Kyoto Encyclopedia of Genes and Genomes (KEGG), genes with a mean fold change greater than 2 or less than 0.5 were selected.

### Organotypic tumor spheroid preparation and microfluidic culture

Tumor samples were collected and processed under Institutional Review Board (IRB)-approved protocols (02-051) at the Dana-Farber/Harvard Cancer Center, with written informed consent obtained from all participants. Patient-derived organotypic tumor spheroids (PDOTS) were generated and cultured as previously described^65^. For the experiments, PDOTS were exposed to FGFR inhibitors for 7 days to model sustained pathway inhibition under ex vivo conditions prior to downstream analyses.

### Single-nucleus GEM generation and gene expression library construction

Nuclei were isolated using a standardized protocol with low-retention microcentrifuge tubes (Fisher Scientific) to minimize nuclei loss^66^. Briefly, tumor tissues were manually dissociated with fine spring scissors for 10 minutes, homogenized in TST buffer, filtered through a 30-μm MACS SmartStrainer (Miltenyi Biotec), and pelleted by centrifugation at 500 × g for 10 minutes at 4ْ°C. Nuclei pellets were further purified using Anti-Nucleus MicroBeads (Miltenyi Biotec, #130-132-997) following the manufacturer’s instructions.

Purified nuclei were resuspended in 100 μL resuspension buffer (1X PBS supplemented with 1% BSA). Dual-fluorescent staining was performed using ViaStain^TM^ AOPI Staining Solution (Nexcelom Bioscience, #CS2-0106). Nuclei quality and concentration were assessed using a Cellometer-K2 Fluorescent Cell Counter (Nexcelom Bioscience). Although partial loss of nuclei integrity was observed, overall nuclei yield and quality were sufficient for downstream processing.

Single-nucleus gel bead-in-emulsion (GEM) generation was performed using the Chromium X system (10X Genomics) according to the Chromium Next GEM Single Cell 5’ Reagent Kits v2 (Dual Index) protocol. Sequencing-ready gene expression libraries were generated following the standard 10X Genomics 5′ workflow.

### snRNA-seq data analysis

#### Dataset quality control

snRNA-seq data were processed using the Cell Ranger pipeline (version 7.1.0, 10x Genomics)^67^. Reads were aligned to the mouse reference genome mm10 (refdata-gex-mm10-2020-A), followed by barcode assignment and UMI counting using the cellranger count workflow. Filtered feature-barcode matrices were imported into Scanpy (version 1.10.0) for downstream analysis^68^. Doublets were identified and removed using Scrublet (version 0.2.3)^69^. Nuclei with ≥500 detected genes, mitochondrial gene content <20%, and hemoglobin gene content <5% were retained for further analysis.

#### Cell clustering

Normalization, scaling, dimensionality reduction, clustering, and differential gene expression analyses were performed using Scanpy (version 1.10.0). Total-count normalization and log transformation were applied using “normalize_total”, followed by data scaling with “scale”. The top 2,000 highly variable genes were identified using “highly_variable_genes” and used for principal component analysis (PCA). Batch effects were corrected using “Harmony” applied to principal components^70^. A k-nearest neighbor graph was constructed using the first 30 principal components, and Uniform Manifold Approximation and Projection (UMAP) was used for visualization^71^. Differentially expressed genes (DEGs) were identified using the “rank_genes_groups” with default parameters.

#### Cell annotation

Cell annotation was assigned based on curated marker gene sets refined from published references and validated across multiple datasets^72,73^. Epithelial cells were identified by expression of *Cd9*, *Cd24a*, *Sox9*, *Cdh1*, and *Epcam*; fibroblasts by *Pdgfrb*, *Fap*, *Lum*, and *Dcn*; and endothelial cells by *Cdh5* and *Pecam1*. T-cell subsets were classified as CD4⁺ naïve (*Cd4*, *Tcf7*, *Il7r*, *Ccr7*), CD8⁺ naïve (*Cd8a*, *Cd8b1*, *Tcf7*, *Il7r*, *Ccr7*, *Sell*), CD4⁺ exhausted (*Cd4*, *Ctla4*, *Pdcd1*, *Havcr2*, *Lag3*), CD8⁺ exhausted (*Cd8a*, *Cd8b1*, *Ctla4*, *Pdcd1*, *Havcr2*), CD4⁺ regulatory (*Foxp3*, *Il2ra*, *Tnfrsf4*, *Itgae*), and CD8⁺ cytotoxic (*Cd8a*, *Cd8b1*, *Gzma*, *Gzmb*, *Prf1*, *Mki67*). Myeloid populations were annotated as M1 macrophages (*Il1a*, *Il1b*, *Tnf*, *Cxcl10*, *Nos2*), M2 macrophages (*Apoe*, *Cd163*, *Arg1*, *Irf4*), and dendritic cells (*Itgax*, *Cd209a*, *Zbtb46*, *Flt3*). Additional immune subsets included natural killer (NK) cells (*Klrd1*, *Cd94*). Final cell identities were determined by integrating canonical marker expression with unsupervised clustering to ensure consistency across samples.

#### RNA velocity analysis

RNA velocity analysis was performed to infer dynamic cell state transitions. Spliced and unspliced transcript counts were generated using velocyto (version 0.17.17) in run10x mode^74^. Velocity moments were computed using scVelo (version 0.3.2)^75^, and gene-specific splicing kinetics were inferred using *recover_dynamics*. RNA velocities were estimated in *dynamical* mode and visualized using partition-based graph abstraction (PAGA)^76^.

#### Survival analysis

Survival analyses were performed using the R packages survival (version 3.5.5) and survminer (version 0.4.9).

### Statistical analysis

Quantitative data are presented as means ± standard error of the mean (SEM). Statistical analyses were performed using unpaired two-tailed Student’s t-test for comparisons between two groups and one-way analysis of variance (ANOVA) for comparisons among three or more groups, as indicated in the figure legends. All statistical analyses were conducted using GraphPad Prism (version 8.0.2). A *p*-value < 0.05 was considered statistically significant.

### Reporting summary

Further information on research design is available in the Nature Portfolio Reporting Summary linked to this article.

## Supplementary information

Supplementary Information

reporting summary

Transparent Peer Review file

## Source data

Source Data

## Data Availability

The transcriptomic, snRNA-seq, and genome sequencing data generated in this study have been deposited in the Gene Expression Omnibus (GEO) under accession numbers: GSE292814GSE311373. Publicly available datasets analyzed in this study are available in GEO under accession number: GSE225691. The remaining data are available within the Article, Supplementary Information or Source Data file. Source data are provided with this paper.
