# Supplementary material for: Targeting FGFR signaling overcomes therapeutic resistance and immune evasion in oncogenic PIK3CA-driven serous-like endometrial cancer
Source: Nat Commun. 2026 May 4;17:5994. doi: 10.1038/s41467-026-72544-z (PMC13347040; doi:10.1038/s41467-026-72544-z)
Supplement: Supplementary file 1 — Supplementary Information [file 41467_2026_72544_MOESM1_ESM.pdf]

Supplementary Information

Targeting FGFR signaling overcomes therapeutic resistance and immune evasion in oncogenic PIK3CA-driven serous-like endometrial cancer

Xin Cheng[1,2], Yadong Zhang[3], Changli Qian[1], Erica Holdridge[4], Guruprasad Ananda[4], Tao Jiang[1], Jing Ni[1,2], Shaozhen Xie[1], Hao Gu[1,2], Renlei Ji[1,2], Elena V. Ivanova[5,6], Marisa R. Nucci[7], Zhe Wang[1], Kaifu Chen[3], Bose Kochupurakkal[6], Gordon J. Freeman[6], Geoffrey I. Shapiro[6], Joyce Liu[6,8], Panagiotis A. Konstantinopoulos[6,8], Ursula Matulonis[6,8], Jean J. Zhao[1,2,9,10*].

[1]Department of Cancer Biology, Dana-Farber Cancer Institute, Boston, MA, USA
[2]Department of Biological Chemistry and Molecular Pharmacology, Harvard Medical School, Boston, MA, USA
[3]Basic and Translational Research Division, Department of Cardiology, Boston Children's Hospital, Boston, MA, USA
[4]Department of Data Science, Dana-Farber Cancer Institute, Boston, MA, USA
[5]Belfer Center for Applied Cancer Science, Dana-Farber Cancer Institute, Boston, MA, USA
[6]Department of Medicine, Brigham and Women's Hospital, Harvard Medical School, Boston, MA, USA
[7]Department of Pathology, Brigham and Women's Hospital, Harvard Medical School, Boston, MA, USA
[8]Division of Gynecologic Oncology, Department of Medical Oncology, Dana-Farber Cancer Institute, Boston, MA, USA
[9]Broad Institute of Harvard and MIT, Cambridge, MA, USA
[10]Laboratory of Systems Pharmacology, Harvard Medical School, Boston, MA, USA.

*Corresponding author: jean_zhao@dfci.harvard.edu (J.J.Z.)

Table of contents:

Supplementary Figures 1-7
Supplementary Tables 1-2

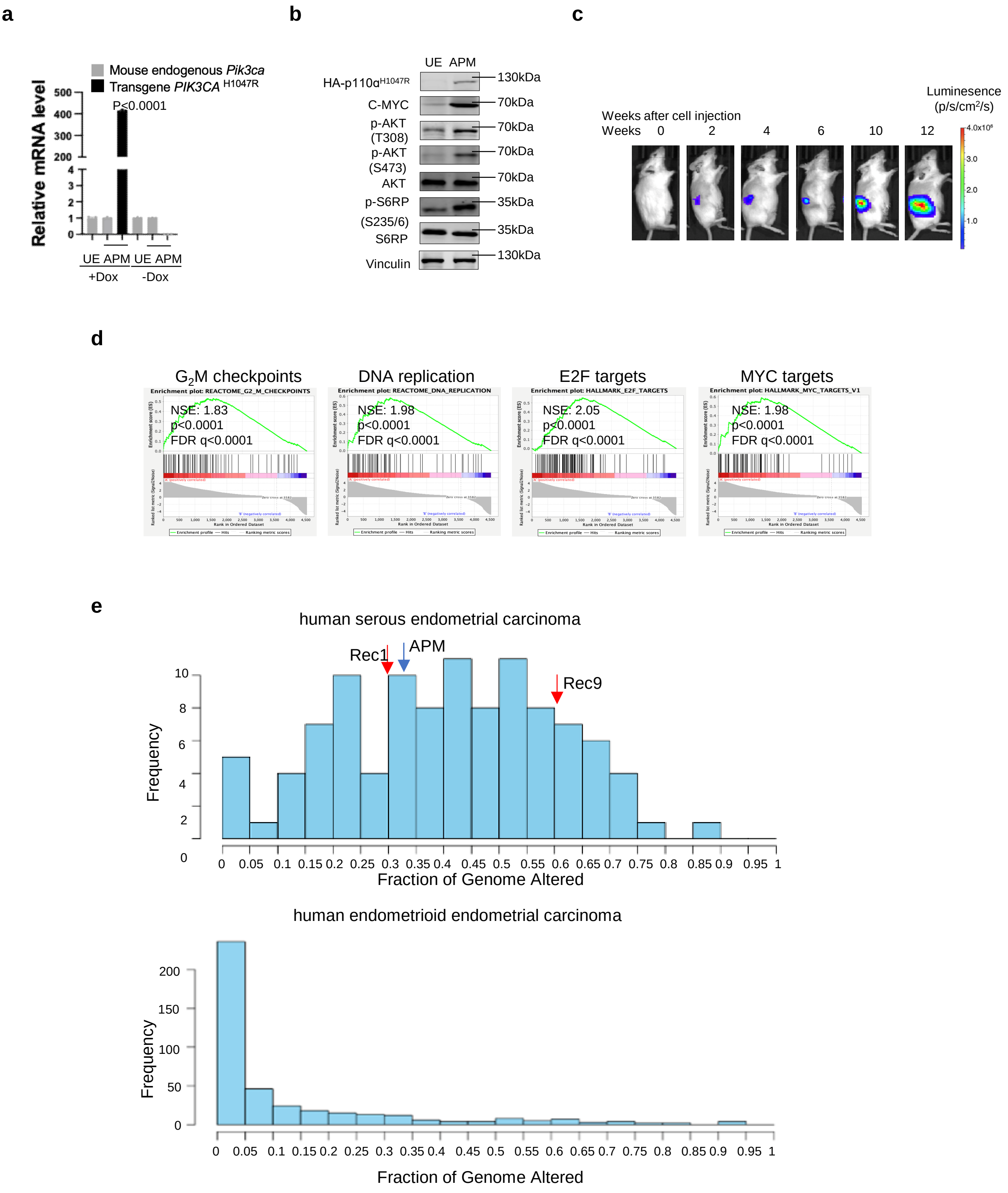

		APM		Rec1		Rec9	
Method	Total numbers of bases	Number of CNV	FGA	Number of CNV	FGA	Number of CNV	FGA
CNVkit (exome)	912677426	299641482	0.33	268929392	0.3	559340263	0.61

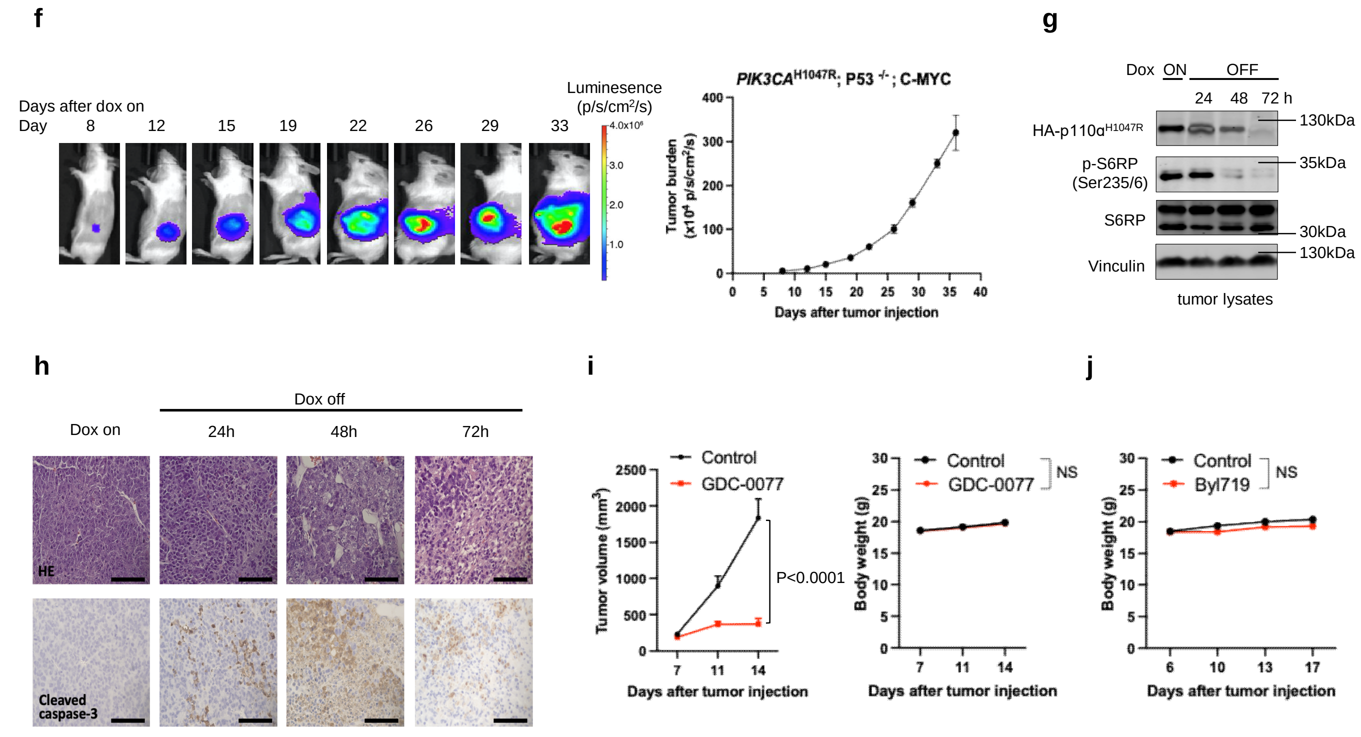

Supplementary Fig. 1. Establishment and characterization of the PIK3CA^{H1047R}; Trp53$^{-/-}$; Myc mouse model. (a) RT-qPCR of the tet-inducible PIK3CA^{H1047R} transgene and endogenous Pik3ca in uterine tissues from tumor-bearing mice with (+) or without (-) doxycycline. n = 3 biologically independent mice per group. Data are shown as mean ± s.e.m. Statistical significance was determined by two-tailed unpaired Student's t-test. **(b)** Immunoblotting analysis of PIK3CA, c-MYC, and downstream signaling components. UE, uterine surface epithelial (normal) cells. Experiments were independently repeated three times with similar results. **(c)** Representative longitudinal in vivo bioluminescence imaging of one mouse illustrating induction of tet-regulated PIK3CA^{H1047R} expression over time (n=5 mice per group). Similar tumor induction kinetics were observed across all mice. **(d)** RNA-seq-based GSEA comparing APM tumors with a published PTEN-loss EC GEMM reveals strong enrichment of G2M checkpoint, DNA replication stress-related programs, E2F targets, and MYC target pathways in APM tumors relative to the PTEN-loss model, consistent with molecular features of human SEC. Data are presented as normalized enrichment scores (NES) with FDR-adjusted q values, based on n = 3 biologically independent tumors per genotype. **(e)** Distribution of FGA in TCGA SEC demonstrates a high burden of CNV (median FGA = 0.41), whereas non-serous TCGA EC samples show a markedly lower alteration burden (median FGA ≈ 0.07). The APM primary tumor model and its recurrent tumors (Rec1 and Rec9) exhibit FGA values of 0.30 and 0.61, respectively, comparable to those observed in human SEC. **(f)** Representative bioluminescence imaging of mice bearing orthotopic, luciferase-expressing APM allografts (n=3 mice per group). Similar tumor growth patterns were observed in all mice. **(g)** Immunoblot analysis of APM tumors with doxycycline on versus after withdrawal. Experiments were independently repeated three times with similar results. **(h)** H&E staining of APM tumors with doxycycline on versus after withdrawal (scale bar, 50 µm). Representative images from n = 3 independent tumors per condition. **(i)** Tumor volume (n = 8 tumors in the vehicle group and n = 10 tumors in the GDC-0077 treatment group) and body weight (n=5 mice per group) changes in APM allografts treated with GDC-0077 versus vehicle. Tumor growth data are shown as mean ± s.e.m. Statistical significance was determined by two-way ANOVA with multiple-comparison correction. Body weight data are shown as mean ± s.e.m.; no statistically significant differences were observed (two-way ANOVA, P = 0.91). **(j)** Body weight trajectories of control- and BYL719-treated mice over time (n = 5 mice per group). No statistically significant differences were observed (two-way ANOVA, P = 0.74). No treatment-related toxicity or significant body-weight loss was observed. Source data are provided as a Source Data file.

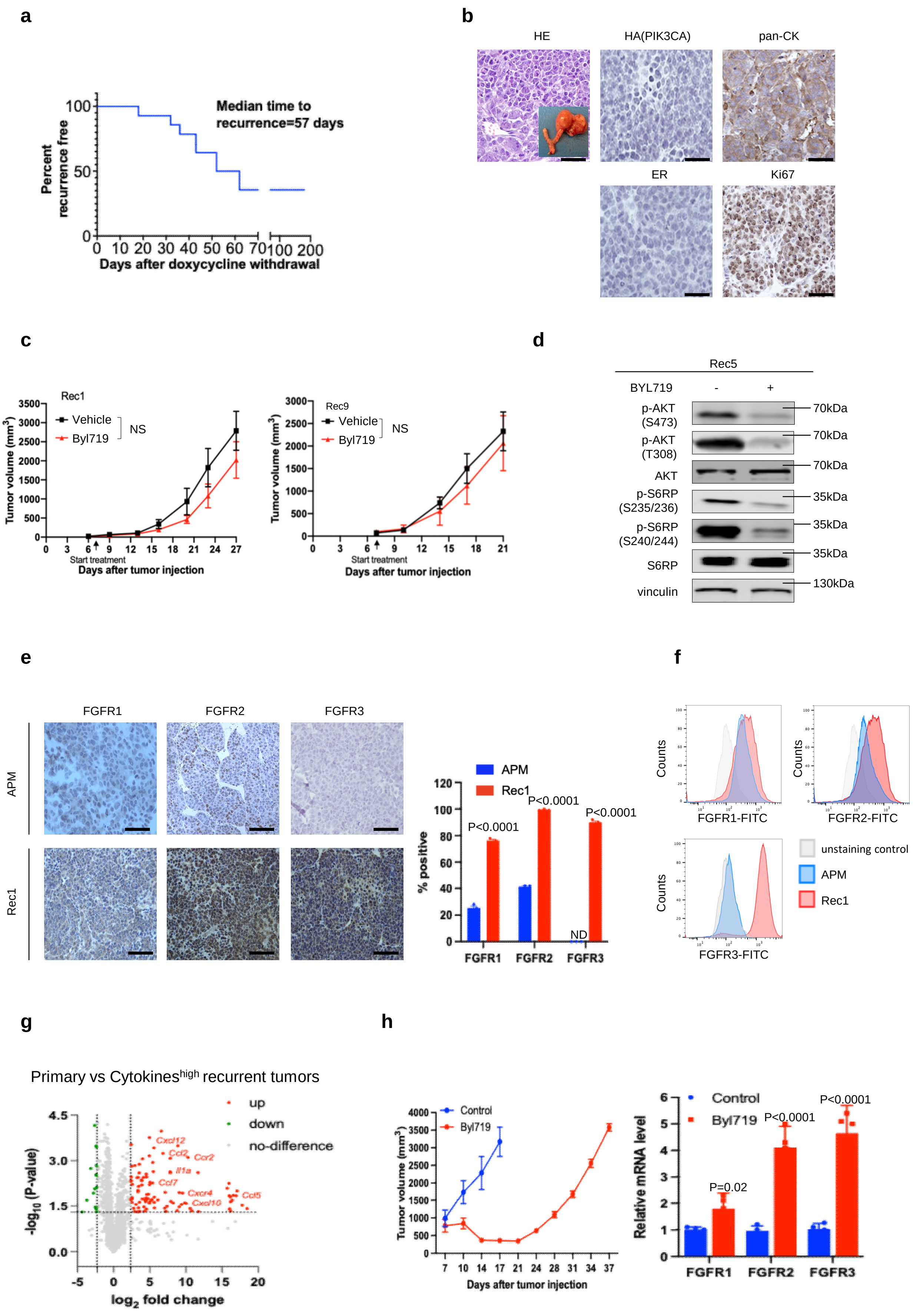

a
Median time to recurrence=57 days
Percent recurrence free
100
50
0
0 10 20 30 40 50 60 70 100 200
Days after doxycycline withdrawal
b
HE
HA(PIK3CA)
pan-CK
ER
Ki67
c
Rec1
Vehicle
Byl719
NS
Tumor volume (mm³)
Start treatment
Days after tumor injection
Rec9
d
Rec5
BYL719
-
+
p-AKT (S473)
p-AKT (T308)
AKT
p-S6RP (S235/236)
p-S6RP (S240/244)
S6RP
vinculin
70kDa
35kDa
130kDa
e
FGFR1
FGFR2
FGFR3
APM
Rec1
% positive
P<0.0001
ND
f
Counts
FGFR1-FITC
FGFR2-FITC
FGFR3-FITC
unstaining control
APM
Rec1
g
Primary vs Cytokines^high recurrent tumors
up
down
no-difference
Cxcl12
Ccl2
Ccr2
Il1a
Ccl7
Cxcr4
Cxcl10
Ccl5
-log10 (P-value)
log2 fold change
h
Control
Byl719
Tumor volume (mm³)
Days after tumor injection
Relative mRNA level
P=0.02
P<0.0001

Supplementary Fig. 2. Characterization of recurrent tumors following PIK3CA^{H1047R} withdrawal. (a) Kaplan-Meier analysis of time to tumor recurrence following doxycycline withdrawal (n = 14 tumors). Median time to recurrence was 57 days. Survival curves were generated using the Kaplan-Meier method. **(b)** Representative immunohistochemistry images from n = 3 biologically independent recurrent tumors (scale bar, 50 μm). **(c)** Tumor growth curves of recurrent tumors Rec1 and Rec9 treated with BYL719 (n=8 tumors per group). Data are shown as mean ± SD. No statistically significant differences were observed (two-way ANOVA, Rec1: P = 0.42; Rec9: P = 0.59). **(d)** Representative immunoblot analysis of Rec5 tumor cells treated with BYL719. Experiments were independently repeated three times with similar results. **(e, f)** Immunohistochemistry (e) and flow cytometric analysis (f) showing FGFR expression in primary versus recurrent tumors. Representative images are shown; similar results were obtained in three biologically independent tumors per group. Quantification is shown as mean ± s.e.m.; n = 3 biologically independent tumors per group. Statistical significance was determined by two-tailed unpaired Student's t-test. **(g)** Volcano plot illustrating significantly altered gene expression in Cytokines^{high} recurrent tumors based on transcriptomic analysis. (**h**) Tumor growth curves of primary APM tumors treated daily with BYL719 (20 mg/kg; n=8 tumors per group). Data are shown as mean ± s.e.m. Right, FGFR expression levels in vehicle-treated control tumors and APM allografts after one month of BYL719 treatment. Data are shown as mean ± s.e.m.; n = 4 biologically independent tumors per group. Statistical significance was determined by two-tailed unpaired Student's t-test. Source data are provided as a Source Data file.

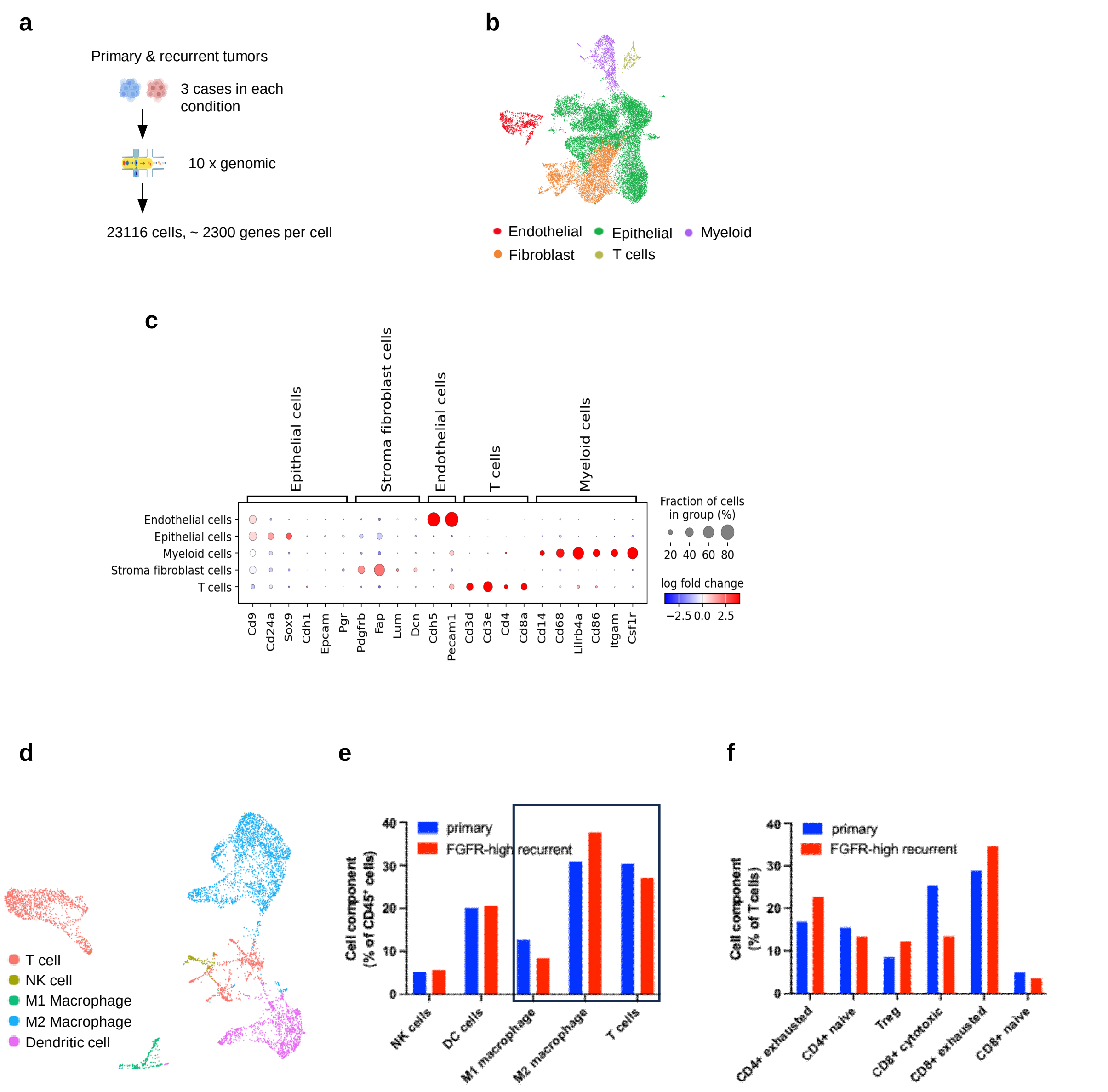

Supplementary Fig. 3. snRNA-seq analysis of FGFR-high recurrent tumors following *PIK3CA*^{H1047R} withdrawal. (a) Schematic illustrating the single-nucleus RNA-sequencing workflow for primary and recurrent tumors (n = 3 biologically independent tumors per group, pooled prior to sequencing). Created in BioRender. Gu, H. (2026) https://BioRender.com/56id7q5. **(b)** UMAP projection of 23,116 nuclei derived from primary and recurrent tumors, showing major cell populations including epithelial, fibroblast, endothelial, myeloid, and T cells. **(c)** Dot plot showing expression of canonical marker genes and differentially expressed genes (DEGs) across major cell types. Dot size represents the fraction of cells expressing each gene; color indicates scaled average expression. **(d)** UMAP projection of CD45^{+} cells from primary and recurrent tumors, reclustered into T cells, NK cells, M1- and M2-like macrophages, and dendritic cells. **(e)** Quantification of immune cell subsets in primary and recurrent tumors, showing increased M2-like macrophages with concomitant reductions in M1-like macrophages and total T cells in FGFR-high recurrent tumors. **(f)** Composition of T-cell subsets in primary versus recurrent tumors, demonstrating depletion of cytotoxic CD8^{+} T cells and enrichment of exhausted T cells and Tregs in recurrent tumors. Three biologically independent tumors per condition were pooled prior to library preparation; therefore, each condition represents one pooled biological sample. Differences are descriptive, and no statistical testing was performed. Source data are provided as a Source Data file.

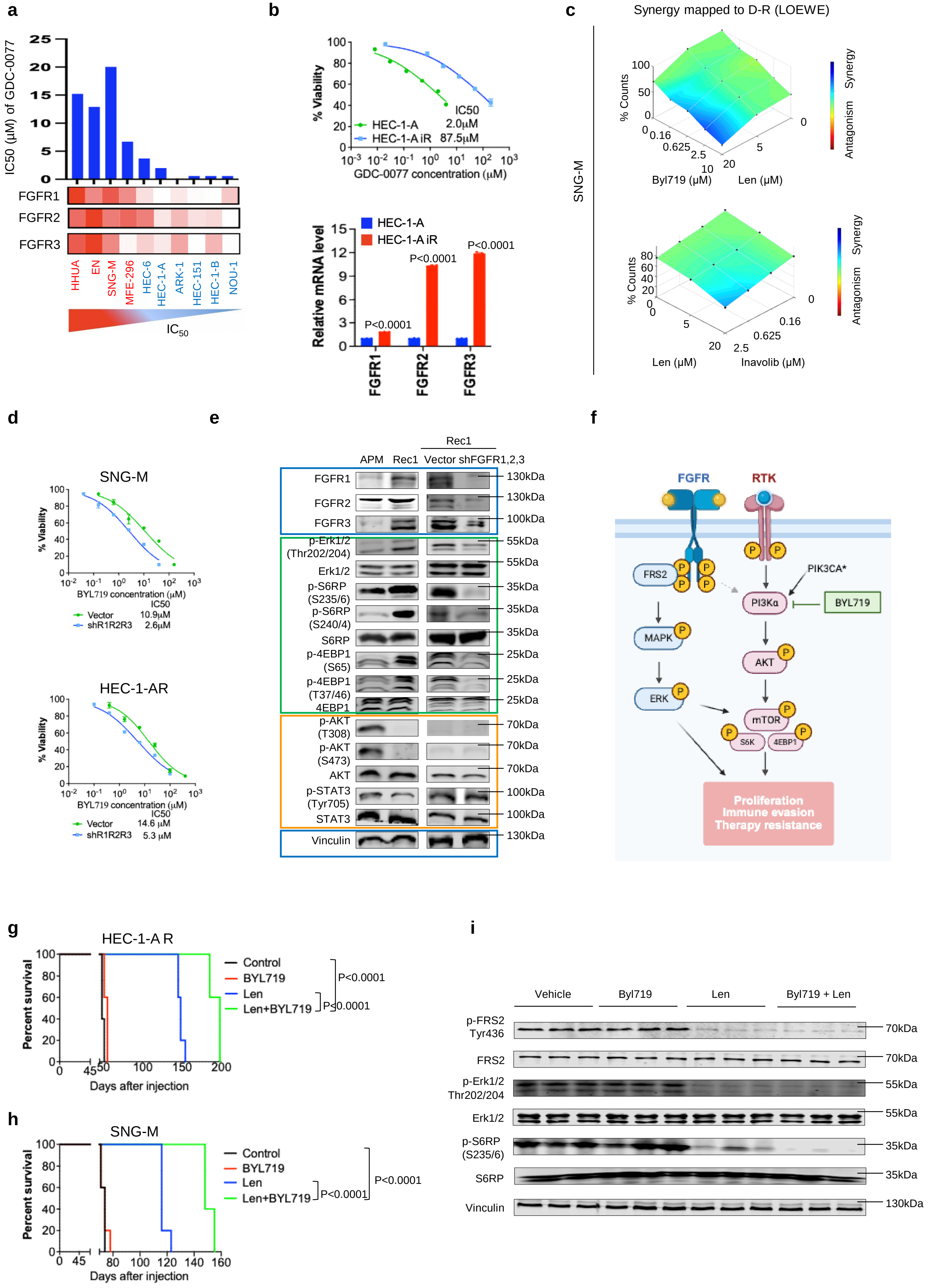

a
IC50 (μM) of GDC-0077
FGFR1
FGFR2
FGFR3
HHUA
EN
SNG-M
MFE-296
HEC-6
HEC-1-A
ARK-1
HEC-151
HEC-1-B
NOU-1
IC50
b
% Viability
GDC-0077 concentration (μM)
IC50
HEC-1-A 2.0μM
HEC-1-A iR 87.5μM
Relative mRNA level
HEC-1-A
HEC-1-A iR
P<0.0001
FGFR1
FGFR2
FGFR3
c
Synergy mapped to D-R (LOEWE)
SNG-M
% Counts
Byl719 (μM)
Len (μM)
Inavolib (μM)
Synergy
Antagonism
d
SNG-M
HEC-1-AR
% Viability
BYL719 concentration (μM)
IC50
Vector 10.9μM
shR1R2R3 2.6μM
Vector 14.6 μM
shR1R2R3 5.3 μM
e
Rec1
APM
Rec1
Vector
shFGFR1,2,3
FGFR1
FGFR2
FGFR3
p-Erk1/2 (Thr202/204)
Erk1/2
p-S6RP (S235/6)
p-S6RP (S240/4)
S6RP
p-4EBP1 (S65)
p-4EBP1 (T37/46)
4EBP1
p-AKT (T308)
p-AKT (S473)
AKT
p-STAT3 (Tyr705)
STAT3
Vinculin
130kDa
100kDa
55kDa
35kDa
25kDa
70kDa
f
FGFR
RTK
FRS2
PIK3CA*
PI3Kα
BYL719
MAPK
AKT
ERK
mTOR
S6K
4EBP1
Proliferation
Immune evasion
Therapy resistance
g
HEC-1-A R
Percent survival
Days after injection
Control
BYL719
Len
Len+BYL719
P<0.0001
h
SNG-M
i
Vehicle
Byl719
Len
Byl719 + Len
p-FRS2 Tyr436
FRS2
p-Erk1/2 Thr202/204
Erk1/2
p-S6RP (S235/6)
S6RP
Vinculin
70kDa
55kDa
35kDa
130kDa

j

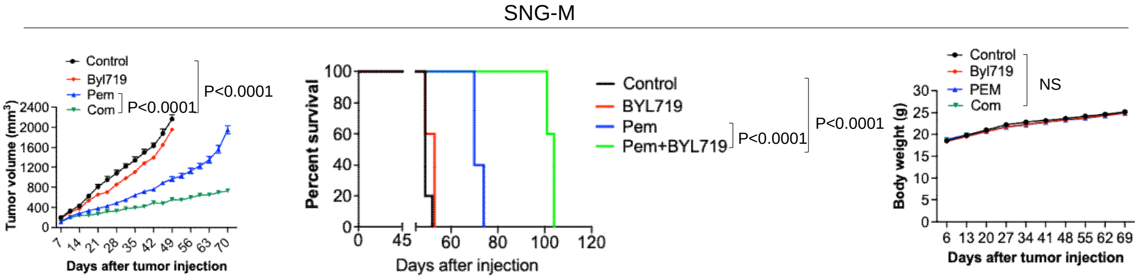
SNG-M
Control
Byl719
Pem
Com
P<0.0001
P<0.0001
Tumor volume (mm³)
Days after tumor injection
Percent survival
Days after injection
Control
BYL719
Pem
Pem+BYL719
P<0.0001
P<0.0001
Control
Byl719
PEM
Com
NS
Body weight (g)
Days after tumor injection

k

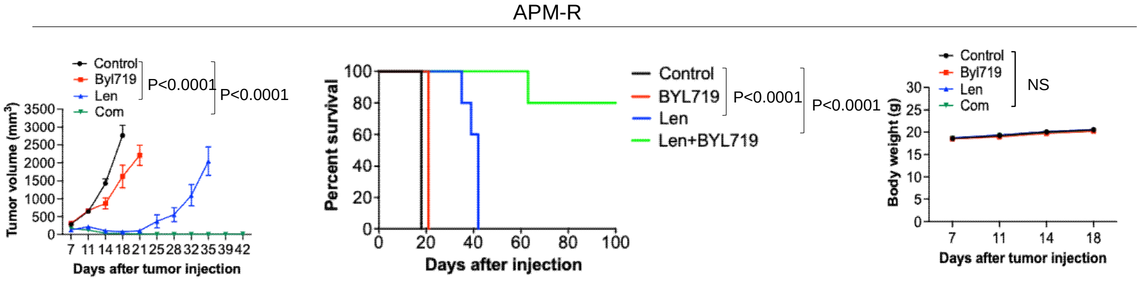
APM-R
Control
Byl719
Len
Com
P<0.0001
P<0.0001
Tumor volume (mm³)
Days after tumor injection
Percent survival
Days after injection
Control
BYL719
Len
Len+BYL719
P<0.0001
P<0.0001
Control
Byl719
Len
Com
NS
Body weight (g)
Days after tumor injection

l

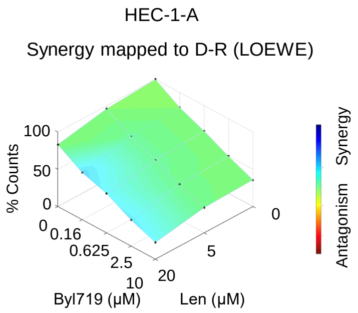
HEC-1-A
Synergy mapped to D-R (LOEWE)
% Counts
Byl719 (μM)
Len (μM)
Synergy
Antagonism

m

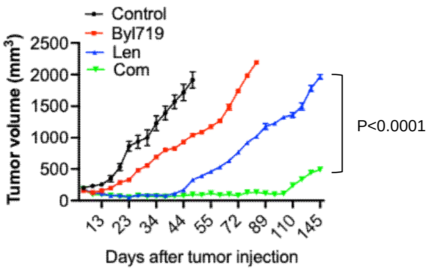
Control
Byl719
Len
Com
Tumor volume (mm³)
P<0.0001
Days after tumor injection

Supplementary Fig. 4. FGFR signaling limits sensitivity to PI3Kα inhibition. (a) IC_{50} values for GDC-0077 across ten PIK3CA-mutant human EC cell lines. IC_{50} values were calculated from dose-response curves derived from three independent biological experiments. **(b)** Dose-response curves showing sensitivity to inavolisib in HEC-1-A cells (sensitive) and their matched resistant counterpart (up), together with FGFR expression level in the sensitive versus resistant lines (down). Data are presented as mean ± s.e.m.; n = 3 independent biological experiments. Statistical significance was determined by two-tailed unpaired Student's t-test. **(c)** Isobologram analyses showing additive to synergistic effects of BYL719 plus lenvatinib and inavolisib plus lenvatinib on SNG-M cell viability (synergy-antagonism analysis using Combenefit). Experiments were independently repeated three times with similar results. **(d)** Effect of FGFR knockdown on BYL719 sensitivity in SNG-M and HEC-1-A R cells. Dose-response curves are representative of three independent biological experiments with similar results. **(e)** Immunoblot analysis of PI3K/AKT, MAPK, and STAT3 signaling in primary tumors, recurrent tumors, and recurrent tumors with FGFR knockdown. Experiments were independently repeated three times with similar results. **(f)** Schematic summary of the proposed mechanism in which FGFR upregulation sustains mTOR signaling through an AKT-independent, FRS2-MAPK-ERK axis despite PI3Kα inhibition, thereby promoting tumor cell survival, therapeutic resistance, and immune evasion. Created in BioRender. Gu, H. (2026) https://BioRender.com/u90bl0l. **(g, h)** Kaplan-Meier survival analyses of HEC-1-A R and SNG-M xenografts treated with lenvatinib and BYL719 (n=5 mice per group). Survival differences were analyzed using the two-sided log-rank (Mantel-Cox) test. **(i)** Immunoblot analysis of SNG-M-derived xenografts treated with the indicated therapies. Experiments were independently repeated three times with similar results. **(j)** Tumor growth curves, Kaplan-Meier survival analyses, and body-weight measurements of SNG-M xenografts treated with pemigatinib plus BYL719 (tumor growth: n = 8 tumors for control, n = 6 tumors for BYL719, n = 10 tumors for pemigatinib, and n = 8 tumors for the combination group; survival and body weight: n = 5 mice per group). Tumor growth data are shown as mean ± s.e.m. Statistical significance was determined by two-way ANOVA with multiple-comparison correction. Survival differences were analyzed using the two-sided log-rank (Mantel-Cox) test. Body weight data are shown as mean ± s.e.m.; no statistically significant differences were observed (two-way ANOVA, P = 0.84). No treatment-related toxicity or significant body weight loss was observed. **(k)** Tumor growth curves, Kaplan-Meier survival analyses, and body weight measurements of BYL719-resistant APM-R tumors treated with BYL719, lenvatinib, or the combination (tumor growth: n = 8 tumors per group; survival and body weight: n = 5 mice per group). Tumor growth data are shown as mean ± s.e.m. Statistical significance was determined by two-way ANOVA with multiple-comparison correction. Survival differences were analyzed using the two-sided log-rank (Mantel-Cox) test. Body weight data are shown as mean ± s.e.m.; no statistically significant differences were observed (two-way ANOVA, P = 0.99). No treatment-related toxicity or significant body weight loss was observed. **(l, m)** Isobologram analysis of BYL719 plus lenvatinib in HEC-1-A cells (diagonal line indicates additivity). Experiments were independently repeated three times with similar results. Tumor growth curves of HEC-1-A xenografts treated with the indicated therapies (n = 6 per group). Data are shown as mean ± s.e.m. Statistical significance was determined by two-way ANOVA with multiple-comparison correction. Source data are provided as a Source Data file.

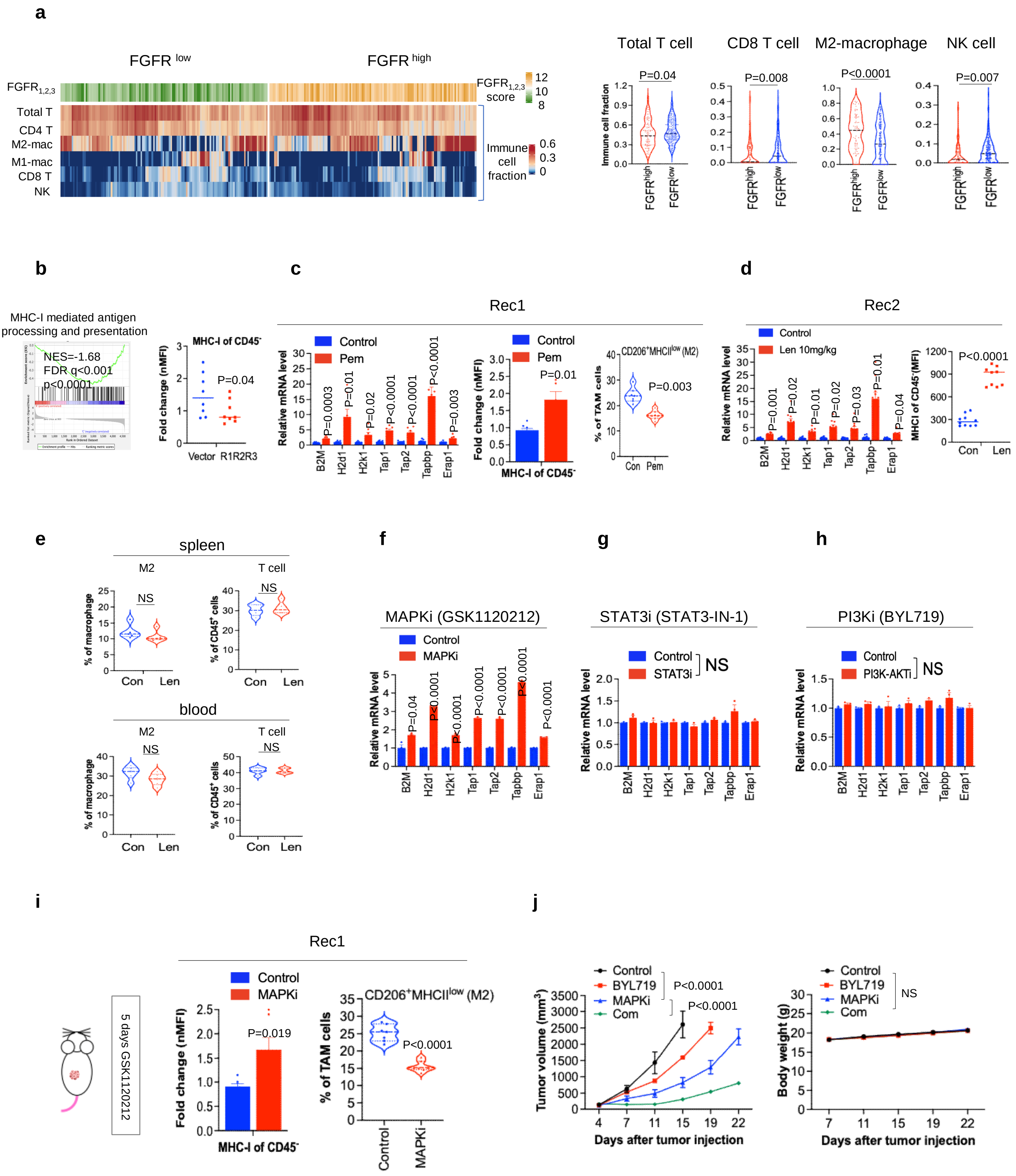
a
FGFR low
FGFR high
FGFR1,2,3
Total T
CD4 T
M2-mac
M1-mac
CD8 T
NK
FGFR1,2,3 score
12
10
8
Immune cell fraction
0.6
0.3
0
Total T cell
CD8 T cell
M2-macrophage
NK cell
P=0.04
P=0.008
P<0.0001
P=0.007
Immune cell fraction
FGFRhigh
FGFRlow
b
MHC-I mediated antigen processing and presentation
NES=-1.68
FDR q<0.001
p<0.0001
MHC-I of CD45-
Fold change (nMFI)
P=0.04
Vector
R1R2R3
c
Rec1
Control
Pem
Relative mRNA level
P=0.0003
P=0.01
P=0.02
P<0.0001
P<0.0001
P<0.0001
P=0.003
B2M
H2d1
H2k1
Tap1
Tap2
Tapbp
Erap1
Fold change (nMFI)
P=0.01
MHC-I of CD45-
CD206+MHCIIlow (M2)
% of TAM cells
P=0.003
Con
Pem
d
Rec2
Control
Len 10mg/kg
P=0.001
P=0.02
P=0.01
P=0.02
P=0.03
P=0.01
P=0.04
MHCI of CD45-(MFI)
P<0.0001
Con
Len
e
spleen
M2
T cell
NS
% of macrophage
% of CD45+ cells
blood
f
MAPKi (GSK1120212)
Control
MAPKi
P=0.04
P<0.0001
P<0.0001
P<0.0001
P<0.0001
P<0.0001
P<0.0001
g
STAT3i (STAT3-IN-1)
Control
STAT3i
NS
h
PI3Ki (BYL719)
Control
PI3K-AKTi
NS
i
Rec1
5 days GSK1120212
Control
MAPKi
P=0.019
Fold change (nMFI)
MHC-I of CD45-
CD206+MHCIIlow (M2)
% of TAM cells
P<0.0001
Control
MAPKi
j
Control
BYL719
MAPKi
Com
P<0.0001
P<0.0001
Tumor volume (mm3)
Days after tumor injection
NS
Body weight (g)
Days after tumor injection

Supplementary Fig. 5. FGFR inhibition enhances antigen presentation and reduces M2-like TAMs. (a) Heatmap showing relative FGFR1-3 expression and immune cell composition across uterine cancer samples from the TCGA UCEC cohort. A composite FGFR1-3 score was calculated for each sample as the mean $\log_2$ expression of FGFR1, FGFR2, and FGFR3. Immune cell fractions were estimated using CIBERSORT and normalized to sum to 1 per sample. Samples were ordered by increasing FGFR composite score and stratified into FGFR-low and FGFR-high groups defined by the bottom and top quartiles of the FGFR1-3 composite score (n = 66 each; left). Violin plots showing CIBERSORT-estimated immune cell fractions in FGFR-high tumors (top quartile, n = 66) compared with all remaining tumors (FGFR low/others, n=198). Statistical significance was determined using two-sided Wilcoxon rank-sum tests. **(b)** Downregulation of antigen presentation genes and MHC-I expression in primary tumors overexpressing FGFRs, assessed by GSEA and flow cytometry. Data are shown as mean ± s.e.m.; n = 8 biologically independent tumors. Statistical significance was determined by two-tailed unpaired Student's t-test. **(c, d)** Upregulation of antigen presentation-related genes and reduction of M2-type TAM populations in recurrent tumors following pemigatinib (c) or lenvatinib (d) treatment. In (c), RT–qPCR analysis was performed with n = 6 biologically independent tumors for the control group and n = 7 biologically independent tumors for the pemigatinib group, and flow cytometry analysis with n = 7 biologically independent tumors per group. In (d), RT–qPCR analysis was performed with n = 6 biologically independent tumors per group, and flow cytometry analysis with n = 10 biologically independent tumors per group. Data are shown as mean ± s.e.m. Statistical significance was determined by two-tailed unpaired Student's t-test. **(e)** Flow cytometric analysis of M2-like macrophages and T cells in the spleen and peripheral blood of naïve mice treated with vehicle or lenvatinib. Data are shown as mean ± s.e.m.; n = 5 mice per group. Statistical significance was determined by two-tailed unpaired Student's t-test. No statistically significant differences were observed between groups (M2 in blood, P = 0.21; T cells in blood, P = 0.96; T cells in spleen, P = 0.82; M2 in spleen, P = 0.28), indicating that, in the absence of tumor, lenvatinib has minimal direct impact on systemic immune cell populations. **(f-h)** RT-qPCR analysis of antigen processing and presentation genes in Rec1 tumor cells treated in vitro with inhibitors targeting major FGFR downstream pathways: MAPK/ERK (MAPKi, GSK1120212), STAT3 (STAT3i, STAT3-IN-1), or PI3K-AKT (PI3Ki, BYL719). Data are shown as mean ± s.e.m.; n = 3 independent biological replicates. Statistical significance was determined by two-tailed unpaired Student's t-test comparing each treatment to control. MAPK inhibition significantly upregulated antigen presentation-related genes, whereas STAT3 or PI3K-AKT inhibition had no significant effect (STAT3i vs control, P = 0.31; PI3Ki vs control, P = 0.15). **(i)** In vivo validation of MAPK-dependent immune modulation in Rec1 tumors. Flow cytometric analysis of tumors from mice treated with GSK1120212 for 5 days shows increased MHC-I expression (MFI) on $CD45^-$ cells (left) and a reduced percentage of $CD206^+MHCII^{low}$ M2-type TAMs (right) compared with vehicle-treated controls (n = 7 tumors per group). Data are shown as mean ± s.e.m.; Statistical significance was determined by two-tailed unpaired Student's t-test. Created in BioRender. Gu, H. (2026) https://BioRender.com/9jli8hr. **(j)** Tumor growth curves and body weight trajectories of mice bearing FGFR-high, BYL719-resistant APM-R tumors treated with BYL719, GSK1120212, or the combination (n = 6 tumors per group). Combination therapy produces the greatest inhibition of tumor growth without affecting body weight, supporting MAPK-ERK as a key functional effector downstream of FGFR in driving tumor progression and resistance. Tumor growth data are shown as mean ± s.e.m. Statistical significance was determined by two-way ANOVA with multiple-comparison correction. Body weight data are shown as mean ± s.e.m.; no statistically significant differences were observed (two-way ANOVA, P = 0.92). Source data are provided as a Source Data file.

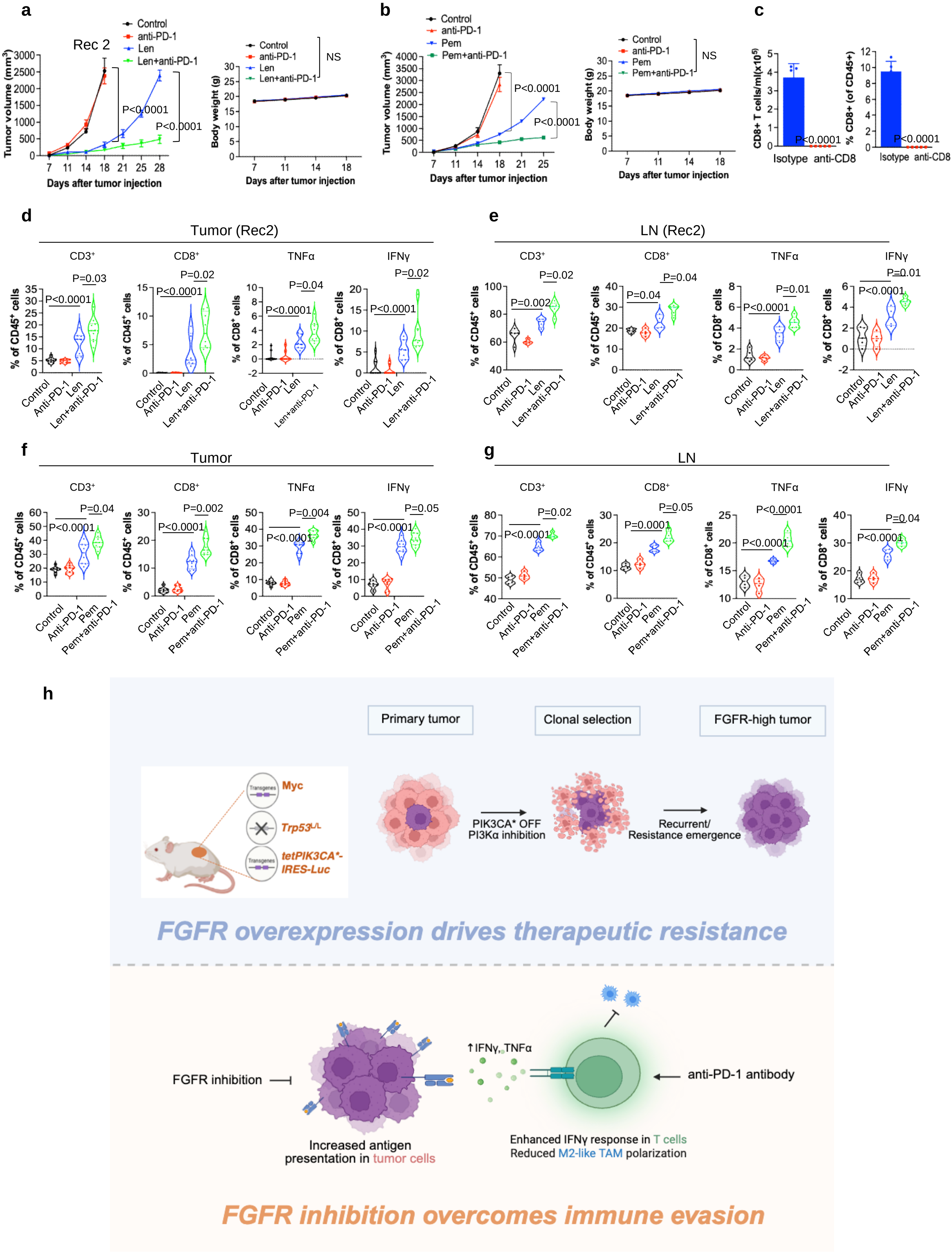
a
Rec 2
Control
anti-PD-1
Len
Len+anti-PD-1
Tumor volume (mm³)
Days after tumor injection
P<0.0001
P<0.0001
Body weight (g)
NS
b
Pem
Pem+anti-PD-1
c
CD8+ T cells/ml(x10⁵)
% CD8+ (of CD45+)
Isotype
anti-CD8
d
Tumor (Rec2)
CD3⁺
CD8⁺
TNFα
IFNγ
% of CD45⁺ cells
% of CD8⁺ cells
Control
Anti-PD-1
Len
Len+anti-PD-1
P=0.03
P=0.02
P=0.04
e
LN (Rec2)
P=0.002
P=0.01
f
Tumor
P=0.04
P=0.002
P=0.004
P=0.05
g
LN
P=0.02
P=0.05
h
Primary tumor
Clonal selection
FGFR-high tumor
Transgenes
Myc
Trp53L/L
tetPIK3CA*-IRES-Luc
PIK3CA* OFF
PI3Kα inhibition
Recurrent/
Resistance emergence
FGFR overexpression drives therapeutic resistance
FGFR inhibition
↑IFNγ, TNFα
anti-PD-1 antibody
Increased antigen
presentation in tumor cells
Enhanced IFNγ response in T cells
Reduced M2-like TAM polarization
FGFR inhibition overcomes immune evasion

Supplementary Fig. 6. FGFR inhibition enhances response to immunotherapy and promotes antitumor immunity. (a) Tumor growth curves and body weight measurements showing the response of recurrent tumor Rec2 to lenvatinib alone or in combination with anti-PD-1 therapy (tumor growth: n = 10 tumors for control, anti-PD-1 and combination groups, and n = 8 tumors for the lenvatinib group; body weight: n = 5 mice per group). Tumor growth data are shown as mean ± s.e.m. Statistical significance was determined by two-way ANOVA with multiple-comparison correction. Body weight data are shown as mean ± s.e.m.; no statistically significant differences were observed (two-way ANOVA, P = 0.97). No treatment-related toxicity or significant body weight loss was observed. **(b)** Tumor growth curves and body weight measurements showing the response of recurrent tumor Rec1 to pemigatinib alone or in combination with anti-PD-1 therapy (tumor growth: n=8 tumors per group; body weight: n = 5 mice per group). Tumor growth data are shown as mean ± s.e.m. Statistical significance was determined by two-way ANOVA with multiple-comparison correction. Body weight data are shown as mean ± s.e.m.; no statistically significant differences were observed (two-way ANOVA, P = 0.99). No treatment-related toxicity or significant body weight loss was observed. **(c)** Flow cytometric analysis of peripheral blood $CD8^+$ T cells following treatment with anti-CD8 or isotype control antibodies (n = 5 mice per group). Data are shown as mean ± s.e.m. Statistical significance was determined by two-tailed unpaired Student's t-test. **(d-g)** Flow cytometric analysis of intratumoral (d, f) and draining lymph node (e, g) $CD3^+$ and $CD8^+$ T-cell populations and effector cytokine production following the indicated treatments. In (d), n = 10 biologically independent tumors for the control group and n = 8 biologically independent tumors for the remaining groups. In (e), n = 5 biologically independent lymph nodes per group. In (f), n = 8 biologically independent tumors per group. In (g), n = 4 biologically independent lymph nodes per group. Data are shown as mean ± s.e.m. Statistical significance was determined by two-tailed unpaired Student's t-test. **(h)** Schematic model summarizing how FGFR signaling promotes therapeutic resistance and immune evasion in PIK3CA-driven serous-like EC by suppressing antigen presentation, limiting $CD8^+$ T-cell infiltration and function, and promoting an immunosuppressive tumor microenvironment. Created in BioRender. Gu, H. (2026) https://BioRender.com/b72wfa4. Source data are provided as a Source Data file.

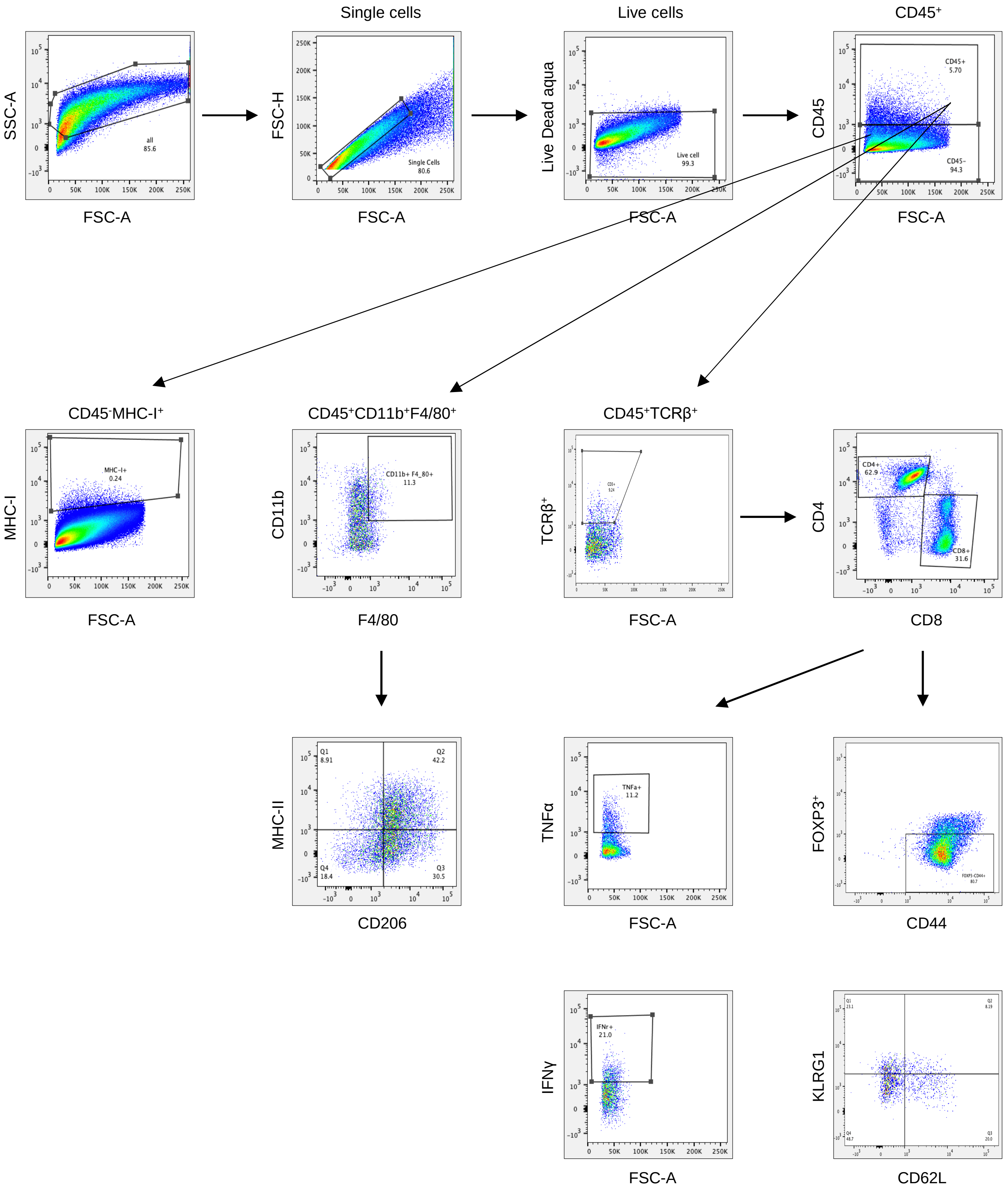

Supplementary Fig. 7. Gating strategies for flow cytometry. All flow cytometry plot axes are displayed on a logarithmic scale, except for forward side scatter (FSC), which is shown on a linear scale.

Supplementary Table 1. A panel of human endometrial cancer cell lines with known mutations in major oncogenes and tumor suppressors

	HHUA	EN	SNG-M	MFE-296	HEC-6	HEC-1-A	ARK-1	HEC-151	HEC-1-B	Nou-1
**PIK3CA**	R88Q	T1025A	R88Q	P539R	R108H	G1049R	E542K	C420R	G1049R	R38H
**TP53**	A138V	R273H	R280T	Y220C	R273H	R248Q	R248W	V31I	R248Q	WT
**PTEN**	V290fs	K267fs	V290*	R130Q	V290*	WT	WT	Y76*	WT	no mRNA
**PIK3R1**	WT	X279-splice	WT	WT	WT	WT	WT	WT	WT	WT
**KRAS**	G12V	WT	G12V	WT	V160A	G12D	WT	WT	D12D	G12D
**EGFR**	WT	WT	WT	WT	A289V	WT	WT	WT	WT	WT
**HER2-amplified**	No	No	No	No	No	No	Yes	No	No	No
**Pathological subtypes**	Endometrial adenocarcinoma	Endometrial carcinoma	Endometrial adenocarcinoma	EEC	Uterine adenosquamous carcinoma	Endometrial carcinoma	SEC	Endometrial carcinoma	Endometrial carcinoma	Endometrial carcinoma
**BYL719 IC_{50} (μM)**	27	16.8	12.3	12.3	5.1	3.2	2.9	2.8	2.4	0.9
**Inavolisib IC_{50} (μM)**	15.2	12.9	20	6.7	3.7	2.0	0.05	0.6	0.6	0.6

EEC, endometrioid endometrial cancer.

Supplementary Table 2. Primer sequences used for RT-qPCR

PIK3CA (human)	forward: 5′- GGAAAAGCTCATTAACTTAAC-3′
	reverse:5′- ATCTGGTCGCCTCATTTGCT-3′
Tp53 (mouse)	forward: 5′-CCCGAGTATCTGGAAGACAG-3′
	reverse: 5′-ATAGGTCGGCGGTTCAT-3′
Myc (mouse)	forward: 5′-CAGAGGAGGAACG AGCTGAAGCGC-3′
	reverse: 5′TTATGCACCAGAGTTTCGAAGCTGTTCG-3′
18S rRNA (mouse)	forward: 5′-CTTAGAGGGACAAGTGGCG-3′
	reverse: 5′-ACGCTGAGCCAGTCGTGTA-3′
Gapdh (mouse)	forward: 5′-ACAACTTTGGCATTGTGGAA-3′
	reverse: 5′-GATGCAGGGATGATGTTCTG-3′
Fgfr1 (mouse)	forward: 5′-TAATACCACCGACAAGGA-3′
	reverse: 5′-TGATGGGGAGTCCGATA-3′
Fgfr2 (mouse)	forward: 5′-GCCTCTCGAACAGTATTC-3′
	reverse: 5′-ACAGGGTTCATAAGGCAT-3′
Fgfr3 (mouse)	forward: 5′-GCCTGCGTGCTAGTGTTC-3′
	reverse: 5′-TACCATCCTTAGCCCAGA-3′
FGFR1 (human)	forward: 5′-AATGAGTACGGCAGCATC-3′
	reverse: 5′-ACCTCGATGTGCTTTAGC-3′
FGFR2 (human)	forward: 5′-GGTGGCTGAAAAACGGGA-3′
	reverse: 5′-AGATGGGACCACACTTTC-3′
FGFR3 (human)	forward: 5′-GCCAAGCCTGTCACCGTA-3′
	reverse: 5′-CAGAAACTCCCGCAGGTT-3′
GAPDH (human)	forward: 5′-GAAGGTGAAGGTCGGAGT-3′
	reverse: 5′-GAAGATGGTGATGGGATT-3′
B2m (mouse)	forward: 5′-TTCTGGTGCTTGTCTCACTGA-3′
	reverse: 5′-CAGTATGTTCGGCTTCCCATTC-3′
H2-D1 (mouse)	forward: 5′-GTGCTGCAGAGCATTACAAG-3′
	reverse: 5′-ATGTAAGAGTCAGTGGACGG-3′
H2-K1 (mouse)	forward: 5′-ATACCTGAAGAACGGGAACG-3′
	reverse: 5′-TCCAAGGACAACCAGAACAG-3′
Tap1 (mouse)	forward:5′-GGACTTGCCTTGTTCCGAGAG-3′
	reverse:5′-GCTGCCACATAAC TGATAGCGA-3′
Tap2 (mouse)	forward:5′-CTGGCGGACATGGCTTTACTT-3′
	reverse:5′-CTCCCACTTTTAGCAGTCCCC-3′
Tapbp (mouse)	forward:5′-GGCCTGTCTAAGAAACCTGCC-3′
	reverse: 5′-CCACCTTGAAGTATAGCTTTGGG-3′
Erap1 (mouse)	forward:5′-TAATGGAGACTCATTCCCTTGGA-3′
	reverse:5′-AAAGTCAGAGTGCTGAGGTTTG-3′
B2M (human)	forward:5′-GAGGCTATCCAGCGTACTCCA-3′
	reverse:5′-CGGCAGGCATACTCATCTTTT-3′
HLA-A (human)	forward:5′-ACCCTCGTCCTGCTACTCTC-3′
	reverse:5′-CTGTCTCCTCGTCCCAATACT-3′
HLA-B (human)	forward: 5′-CAGTTCGTGAGGTTCGACAG-3′
	reverse:5′-CAGCCGTACATGCTCTGGA-3′
HLA-C (human)	forward:5′-GGACAAGAGCAGAGATACACG-3′
	reverse:5′-CAAGGACAGCTAGGACAACC-3′
TAP1 (human)	forward: 5′-TGCCCCGCATATTCTCCCT-3′
	reverse: 5′-CACCTGCGTTTTCGCTCTTG-3′
TAP2 (human)	forward: 5′-TGGACGCGGCTTTACTGTG-3′
	reverse: 5′-GCAGCCCTCTTAGCTTTAGCA-3
TAPBP (human)	forward: 5′-TGGACCGGAAATGGGACCT-3′
	reverse: 5′-CCCCAGAAGGGTAGAAGTGG-3′
ERAP1 (human)	forward: 5′-CCCCTCAAATGGTCCCTTGC-3′
	reverse: 5′-GAGATGCTTCAGTGCTCTGAC-3′
ERAP2 (human)	forward: 5′-CACTAATGGGGAACGATTTCCTT-3′
	reverse: 5′-CTGACCAAGACTTCGATCTTCTC-3′
