# Supplementary material for: Targeting FGFR signaling overcomes therapeutic resistance and immune evasion in oncogenic PIK3CA-driven serous-like endometrial cancer
Source: Nat Commun. 2026 May 4;17:5994. doi: 10.1038/s41467-026-72544-z (PMC13347040; doi:10.1038/s41467-026-72544-z)
Supplement: Supplementary file 2 — reporting summary [file 41467_2026_72544_MOESM2_ESM.pdf]

nature portfolio

Corresponding author(s): Jean J. Zhao

Last updated by author(s): 3/1/2026

Reporting Summary

Nature Portfolio wishes to improve the reproducibility of the work that we publish. This form provides structure for consistency and transparency in reporting. For further information on Nature Portfolio policies, see our Editorial Policies and the Editorial Policy Checklist.

Statistics

For all statistical analyses, confirm that the following items are present in the figure legend, table legend, main text, or Methods section.

n/a	Confirmed	
☐	☒	The exact sample size (*n*) for each experimental group/condition, given as a discrete number and unit of measurement
☐	☒	A statement on whether measurements were taken from distinct samples or whether the same sample was measured repeatedly
☐	☒	The statistical test(s) used AND whether they are one- or two-sided<br>*Only common tests should be described solely by name; describe more complex techniques in the Methods section.*
☒	☐	A description of all covariates tested
☐	☒	A description of any assumptions or corrections, such as tests of normality and adjustment for multiple comparisons
☐	☒	A full description of the statistical parameters including central tendency (e.g. means) or other basic estimates (e.g. regression coefficient) AND variation (e.g. standard deviation) or associated estimates of uncertainty (e.g. confidence intervals)
☐	☒	For null hypothesis testing, the test statistic (e.g. *F*, *t*, *r*) with confidence intervals, effect sizes, degrees of freedom and *P* value noted<br>*Give P values as exact values whenever suitable.*
☒	☐	For Bayesian analysis, information on the choice of priors and Markov chain Monte Carlo settings
☒	☐	For hierarchical and complex designs, identification of the appropriate level for tests and full reporting of outcomes
☐	☒	Estimates of effect sizes (e.g. Cohen's *d*, Pearson's *r*), indicating how they were calculated

Our web collection on statistics for biologists contains articles on many of the points above.

Software and code

Policy information about availability of computer code

Data collection	BD FACSDiva (version 6), Ion Torrent paltform, Illumina sequencing platform.
Data analysis	Prism version 9 (GraphPad), Living Image Software version 4.5 (PerkinElmer), FlowJo (version 9), R packages survival (version 3.5.5), survminer (version 0.4.9), GSEA software (version 4.3.3) , and CNVkit (version 0.9.11).

For manuscripts utilizing custom algorithms or software that are central to the research but not yet described in published literature, software must be made available to editors and reviewers. We strongly encourage code deposition in a community repository (e.g. GitHub). See the Nature Portfolio guidelines for submitting code & software for further information.

Data

Policy information about availability of data

All manuscripts must include a data availability statement. This statement should provide the following information, where applicable:

- Accession codes, unique identifiers, or web links for publicly available datasets
- A description of any restrictions on data availability
- For clinical datasets or third party data, please ensure that the statement adheres to our policy

The transcriptomic, snRNA-seq, and genome sequencing data generated in this study have been deposited in the Gene Expression Omnibus (GEO) under accession numbers:
GSE292814 [https://www.ncbi.nlm.nih.gov/geo/query/acc.cgi?acc=GSE292814]

GSE311373 [https://www.ncbi.nlm.nih.gov/geo/query/acc.cgi?acc=GSE311373]
Publicly available datasets analyzed in this study are available in GEO under accession number: GSE225691 [https://www.ncbi.nlm.nih.gov/geo/query/acc.cgi?acc=GSE225691]
The remaining data are available within the Article, Supplementary Information or Source Data file. Source data are provided with this paper.

Research involving human participants, their data, or biological material

Policy information about studies with human participants or human data. See also policy information about sex, gender (identity/presentation), and sexual orientation and race, ethnicity and racism.

Reporting on sex and gender	Sex and gender were not analyzed as variables in this study. The human tumor samples were de-identified and used for organoid generation.
Reporting on race, ethnicity, or other socially relevant groupings	Race and ethnicity data were not collected and were not analyzed in this study.
Population characteristics	Human tumor sample was obtained from patient undergoing surgical resection at Dana-Farber Cancer Institute. Sample was de-identified prior to use. No additional clinical characteristics were analyzed in this study.
Recruitment	Tumor samples were obtained from patients undergoing clinically indicated procedures. No prospective recruitment was performed for this study.
Ethics oversight	The use of human tumor specimens was approved by the Institutional Review Board of DFCI (protocol #02-051). Written informed consent was obtained from all participants.

Note that full information on the approval of the study protocol must also be provided in the manuscript.

Field-specific reporting

Please select the one below that is the best fit for your research. If you are not sure, read the appropriate sections before making your selection.

☒ Life sciences ☐ Behavioural & social sciences ☐ Ecological, evolutionary & environmental sciences

For a reference copy of the document with all sections, see nature.com/documents/nr-reporting-summary-flat.pdf

Life sciences study design

All studies must disclose on these points even when the disclosure is negative.

Sample size	For animal experiments, sample sizes were determined based on previous experience with the models utilized, including observed variability in tumor growth (Nat Commun. 2022 May 31;13(1):3022; Nature 2023 May;617(7959):139–146). Sample sizes for other experiments are indicated in the relevant figure legends.
Data exclusions	No data were excluded from analysis.
Replication	All experiments were independently repeated at least three times with similar results. Detailed information on biological and technical replicates is provided in the manuscript and figure legends.
Randomization	Mice were randomized into treatment groups when tumors reached comparable average volumes.
Blinding	In vivo drug treatment studies, immunohistochemistry (IHC), and flow cytometry analyses were performed in a blinded manner when feasible. No other experiments required blinding during data collection or analysis.

Reporting for specific materials, systems and methods

We require information from authors about some types of materials, experimental systems and methods used in many studies. Here, indicate whether each material, system or method listed is relevant to your study. If you are not sure if a list item applies to your research, read the appropriate section before selecting a response.

Materials & experimental systems

n/a	Involved in the study
☐	☒ Antibodies
☐	☒ Eukaryotic cell lines
☒	☐ Palaeontology and archaeology
☐	☒ Animals and other organisms
☒	☐ Clinical data
☒	☐ Dual use research of concern
☒	☐ Plants

Methods

n/a	Involved in the study
☒	☐ ChIP-seq
☐	☒ Flow cytometry
☒	☐ MRI-based neuroimaging

Antibodies

Antibodies used

Antibodies for Flow cytometry:
CD45 (1:100, 30-F11, BioLegend), CD3 (1:100, 145-2C11, BioLegend), CD4 (1:100, RM4-5, BioLegend), CD8A (1:100, 53-6.7, BioLegend), CD44 (1:100, IM7, BioLegend), CD62L (1:100, MEL-14, BioLegend), FOXP3 (1:100, MF-14, BioLegend), IFN-γ (1:100, XMG1.2, BioLegend), TNF-α (1:100, MP6-XT22, BioLegend), KLRG1 (1:100, 2F1/KLRG1, BioLegend), H2-Kq (1:00, KH114, BioLegend), HLA-A/B/C (1:100, W6/32, BioLegend), β2-microglobulin (1:100, A17082A, BioLegend), CD11b (1:100, M1/70, BioLegend), F4/80 (1:100, BM8, BioLegend), CD206 (1:100, MMR, BioLegend), FGFR1 (1:50, M19B2, Thermo Fisher Scientific), FGFR2 (1:50, SP273, abcam), and FGFR3 (1:50, H10B4, Thermo Fisher Scientific), goat anti-rabbit IgG (H+L) and goat anti-mouse IgG (H+L) secondary antibodies (1:5000; Life Technologies, #A11008 and #A21235).
Antibodies for Immunohistochemistry:
anti-HA (1:400, Cell Signaling Technology, #3724), anti-pan- cytokeratin (1:200, Abcam, #ab7753), anti-ERα (1:200, Thermo Fisher Scientific, #RM9101S0), anti-Ki67 (1:500, Abcam, #ab15580), anti-cleaved caspase-3 (1:400, Cell Signaling Technology, #9661), anti-FGFR1 antibody (1:50, Abcam, #ab76464), anti-FGFR2 antibody (1:50, Abcam, #ab106648), and anti-FGFR3 antibody (1:50, Abcam, #ab180906).
Antibodies for in vivo treatment
An anti-PD-1 antibody (clone, 332.8H3, mouse IgG1), IgG control antibody (RTK2758, Biolegend), anti-CD8 antibody (53.6.7, Biolegend).
Antibodies for Western blots
HA (1:1000, 3F10, Roche), phospho-S6 ribosomal protein (Ser235/236, 1:1000, Cell Signaling Technology, #2211), phospho-S6 ribosomal protein (Ser240/244, 1:1000, Cell Signaling Technology, #2215), total S6 ribosomal protein (1:1000, Cell Signaling Technology, #2217), MYC (1:1000, Santa Cruz Biotechnology, sc-40), phospho-AKT (Ser473, 1:1000, Cell Signaling Technology, #4060), phospho-AKT (Thr308, 1:1000, Cell Signaling Technology, #2965), total AKT (1:1000, Cell Signaling Technology, #9272), phospho-FRS2 (Tyr436, 1:500, Cell Signaling Technology, #3861), total FRS2 (1:1000, R&D Systems, MAB4069), phospho-ERK1/2 (Thr202/Tyr204, 1:1000, Cell Signaling Technology, #9101), total ERK1/2 (1:1000, Cell Signaling Technology, #9102), phospho-PRAS40 (1:1000, Cell Signaling Technology, #2997), total PRAS40 (1:1000, Cell Signaling Technology, #2691), phospho-p70S6 (1:1000, Cell Signaling Technology, #9205), total p70S6 (1:1000, Cell Signaling Technology, #9202), FGFR1 (1:500, Cell Signaling Technology, #9740), FGFR2 (1:500, Cell Signaling Technology, #23328), FGFR3 (1:500, Cell Signaling Technology, #4574), phospho-4EBP1(Ser65, 1:1000, Cell Signaling Technology, #9451), phospho-4EBP1 (Thr37/46, 1:1000, Cell Signaling Technology, #2855), total 4EBP1 (1:1000, Cell Signaling Technology, #9452), phospho-eIF4B (Ser422, 1:1000, Cell Signaling Technology, #3591), total eIF4B (1:1000, Cell Signaling Technology, #3592), phospho-STAT3 (Tyr705, 1:1000, Cell Signaling Technology, #9145), total STAT3 (1:1000, Cell Signaling Technology, #9139), and vinculin (1:5000, Sigma -Aldrich, V9131).

Validation

Anti-PD-1 antibody (clone, 332.8H3) was generated and validated in the laboratory of Dr. Gordon Freeman at Dana-Farber Cancer Institute (Cancer Immunol Res. 2006 Feb;4 (2) 124-35). All the other antibodies used in this study are commercially available and have been verified by the manufactures.

Eukaryotic cell lines

Policy information about cell lines and Sex and Gender in Research

Cell line source(s)	HEC-1-A(ATCC, HTB-112), HEC-1-B (ATCC, HTB-113), MFE-296 (Sigma Aldrich, 98031101-1VL), ARK1 (courtesy of Dr. Dipanjan Chowdhury, Dana-farber Cancer Institute), HEC-151, HEC-6, HHUA (kindly provided by Dr. Jessie A. Sanai, Memorial Sloan Kettering Cancer Center), and EN, NOU-1, SNG-M (generously provided by Dr. Gordon B. Mills, MD Anderson Cancer Center).
Authentication	All cell lines were rigorously tested for mycoplasma contamination and authenticated using short tandem repeat (STR) profiling.
Mycoplasma contamination	All the cells were test negative for mycoplasma.
Commonly misidentified lines (See ICLAC register)	NA

Animals and other research organisms

Policy information about studies involving animals; ARRIVE guidelines recommended for reporting animal research, and Sex and Gender in Research

Laboratory animals	The TetO-PIK3CAH1047R mouse line was maintained in our laboratory (Nat Med 17, 2011,1116-1120) , and the Tp53loxP/loxP mouse line was obtained from the National Cancer Institute Mouse Repository (FVB.129P2-Trp53tm1Brn/Nci, # 01XC2). These mouse lines were backcrossed for more than 10 generations to the FVB/N background before being intercrossed to generate homozygous lines. FVB/NJ and nude mice were ordered from The Jackson Laboratory.
Wild animals	No wild animals were used in this study.
Reporting on sex	Female mice were used for all experiments, as this study focuses on endometrial cancer models. Sex was therefore not considered as a biological variable.
Field-collected samples	No filed-collected samples were used in this study.
Ethics oversight	All animal experiments in this study were conducted in accordance with animal protocols (03-111, 06-034, 02-127) approved by the Dana-Farber Cancer Institute (DFCI) Institutional Animal Care and Use Committee (IACUC).

Note that full information on the approval of the study protocol must also be provided in the manuscript.

Plants

Seed stocks	*Report on the source of all seed stocks or other plant material used. If applicable, state the seed stock centre and catalogue number. If plant specimens were collected from the field, describe the collection location, date and sampling procedures.*
Novel plant genotypes	*Describe the methods by which all novel plant genotypes were produced. This includes those generated by transgenic approaches, gene editing, chemical/radiation-based mutagenesis and hybridization. For transgenic lines, describe the transformation method, the number of independent lines analyzed and the generation upon which experiments were performed. For gene-edited lines, describe the editor used, the endogenous sequence targeted for editing, the targeting guide RNA sequence (if applicable) and how the editor was applied.*
Authentication	*Describe any authentication procedures for each seed stock used or novel genotype generated. Describe any experiments used to assess the effect of a mutation and, where applicable, how potential secondary effects (e.g. second site T-DNA insertions, mosiacism, off-target gene editing) were examined.*

Flow Cytometry

Plots

Confirm that:

☒ The axis labels state the marker and fluorochrome used (e.g. CD4-FITC).

☒ The axis scales are clearly visible. Include numbers along axes only for bottom left plot of group (a 'group' is an analysis of identical markers).

☒ All plots are contour plots with outliers or pseudocolor plots.

☒ A numerical value for number of cells or percentage (with statistics) is provided.

Methodology

Sample preparation	To obtain single-cell suspensions, tumors were excised, minced and dissociated in collagenase/hyaluronidase buffer (DMEM with 5% FBS, 10 mM HEPES, 100 µg/mL penicillin–streptomycin, 20 µg/mL DNase I, and 1× collagenase/hyaluronidase) for 40-60 min at 37 °C with agitation. After digestion, the suspension was treated with ammonium-chloride-potassium (ACK) buffer to lyse red blood cells (RBCs) and filtered through a 70 µm strainer to remove any undigested tumor tissues. Tumor-draining lymph nodes (TDLNs) were mechanically dissociated by passing the tissues through a 70 µm strainer using the plunger of a 5 mL syringe.
Instrument	LSRFortessa HTS (BD) and FACSAria-II SORP (BD) were used.
Software	FACSDiva HTS (BD) was used to collect the data, and the data was analyzed using FlowJo (BD).
Cell population abundance	After sorting, cells were counted and an equal number was prepared for each sample.
Gating strategy	Gating strategies are included in the Supplementary Information. Gates were set after appropriated compensation by single color stains.

☒ Tick this box to confirm that a figure exemplifying the gating strategy is provided in the Supplementary Information.
