# Supplementary material for: Targeting FGFR signaling overcomes therapeutic resistance and immune evasion in oncogenic PIK3CA-driven serous-like endometrial cancer
Source: Nat Commun. 2026 May 4;17:5994. doi: 10.1038/s41467-026-72544-z (PMC13347040; doi:10.1038/s41467-026-72544-z)
Supplement: Supplementary file 3 — Transparent Peer Review file [file 41467_2026_72544_MOESM3_ESM.pdf]

nature portfolio

Peer Review File

Targeting FGFR signaling overcomes therapeutic resistance and immune evasion in oncogenic PIK3CA-driven serous-like endometrial cancer

Corresponding Author: Professor Jean Zhao

Parts of this Peer Review File have been redacted as indicated to maintain the confidentiality of unpublished data.

This file contains all reviewer reports in order by version, followed by all author rebuttals in order by version.

Version 0:

Reviewer comments:

Reviewer #1

(Remarks to the Author)
This study presents a clinically relevant, immunocompetent mouse model of serous endometrial cancer (SEC) that incorporates three commonly observed genetic alterations in human SEC: PIK3CA mutation, TP53 loss, and MYC overexpression. This model recapitulates pathological and molecular features of a subset of human SEC and provides a valuable platform to investigate mechanisms of therapeutic resistance and immune evasion. The authors demonstrate that SEC tumors initially depend on PIK3CA signaling for growth and survival, but frequently develop resistance through activation of FGFR pathways. Specifically, FGFR1 and FGFR2 are associated with intrinsic resistance, while FGFR3 is linked to acquired resistance following targeted therapy. Furthermore, elevated FGFR signaling promotes immune evasion by downregulating MHC-I and HLA gene expression, resulting in impaired antigen presentation and reduced T cell infiltration. The study shows that combined inhibition of PI3Kα (using BYL719) and FGFR (using lenvatinib) effectively suppresses tumor growth in both primary and recurrent settings. Notably, FGFR blockade restores antigen presentation and enhances the therapeutic efficacy of anti-PD-1 immunotherapy, leading to durable immune responses in preclinical models. These findings highlight the dual role of FGFR signaling in mediating both therapeutic resistance and immune suppression, and support the rationale for combined targeted and immunotherapeutic strategies in the treatment of SEC.

While this model is an advancement on EC (SEC) model, several limitations should be addressed. One of the hallmark features of SEC is extensive genomic instability and widespread copy number alterations. Although TP53 loss is included in the model and might contribute to genomic instability, it remains unclear whether the model faithfully recapitulates the copy number alterations seen in human SEC. The authors should evaluate copy number variation in the model and compare the genomic landscape to human SEC tumors. If the model does not exhibit the high level of copy number alterations that is a hallmark of human SEC, it may not fully qualify as a representative SEC model, despite being a useful genetically engineered mouse model for endometrial cancer in general.

Additionally, it would be beneficial to compare this model to other genetically engineered mouse models of endometrial cancer (there is several EC mouse models, although they are not specifically for SEC) to better define its specificity and relevance to the serous subtype.

The study also employs ten human endometrial cancer cell lines to assess sensitivity to the PI3Kα inhibitor BYL719. However, the histological or molecular subtypes of these cell lines are not clearly defined. Are they all SEC lines? Since different subtypes of endometrial cancer, such as endometrioid and serous, exhibit distinct biological behaviors and drug responses, it is important for the authors to classify the cell lines used in the study to determine their relevance to serous endometrial cancer specifically.

In the analysis of TCGA endometrial cancer patient data, the authors report an inverse correlation between FGFR expression and CD3D expression, suggesting reduced T cell infiltration in FGFR-high tumors. While this is a useful observation, the authors should apply immune deconvolution tools such as CIBERSORT to more accurately assess the composition of immune cell subsets within the bulk tumor transcriptomic data. Importantly, how about other immune cell

populations, and whether they show similar as the model developed by this study.

The single-cell RNA sequencing analysis in this study reveals important insights into the heterogeneity of tumor and immune cell populations, but the immune profiling remains relatively superficial. More detailed characterization of immune cell subtypes, particularly among T cells (such as effector, memory, regulatory) and myeloid cells (including macrophage and dendritic cell subsets), would provide a clearer picture of the immune landscape in primary and recurrent tumors. This deeper resolution would help elucidate how FGFR signaling reshapes the tumor microenvironment and contributes to immune escape.

Another issue is the use of lenvatinib as the FGFR inhibitor in the study. Lenvatinib is a broad-spectrum tyrosine kinase inhibitor that targets not only FGFR1–4 but also VEGFR1–3, RET, KIT, and others. As a result, it is difficult to attribute the observed therapeutic effects solely to FGFR inhibition. To strengthen the mechanistic conclusions, the authors should evaluate more selective FGFR inhibitors, such as erdafitinib or pemigatinib, which would provide cleaner evidence of FGFR-specific contributions to resistance and immune evasion.

While the study shows that FGFR overexpression suppresses antigen presentation and promotes immune escape, the precise downstream signaling mechanisms responsible for this effect remain poorly defined. It would be informative to dissect which downstream pathways, such as MAPK, PI3K-AKT, or STAT signaling, mediate the immunosuppressive effects of FGFR activity. Understanding these mechanisms could further guide rational design of combination therapies.

Reviewer #2

(Remarks to the Author)
In their manuscript entitled “Targeting FGFR signaling overcomes therapeutic resistance and immune evasion in oncogenic PIK3CA-driven serous endometrial cancer,” Cheng et al. leverage a novel, clinically relevant, immunocompetent model of serous endometrial cancer (SEC) driven by PIK3CA mutation (PIK3CA-H1047R), TP53 loss, and c-MYC overexpression to explore mechanisms of resistance to PIK3CA therapy. In this model, FVB mouse uterine epithelial cells carrying Rosa-lsl-rtTA, floxed-Trp53, and tetracycline (tet)-regulated mutant PIK3CA were further modified with adenoviral Cre and lentiviral mouse Myc, and orthotopically transplanted into the uterine wall of FVB female recipients. Using single cell transcriptional profiling, the authors determined that FGFR1/2 signaling is associated with intrinsic resistance to PIK3CA targeted therapies. FGFR3, in turn, was linked to acquired resistance to these agents. Combined inhibition of FGFR and PI3Kα enhanced tumor suppression in this model. Further analysis of the downstream effects of FGFR signaling identified a role for this pathway in immune evasion via suppression of antigen-processing and presentation; FGFR inhibition reversed the effect and synergized with anti-PD-1 therapy. The data are generally of high quality and supportive of the conclusions of the authors. Together, these studies provide strong support for FGFR inhibition as a strategy for the treatment of a subset of patients with SEC. This a compelling study with high significance and should be of interest to the broad readership of Nature Communications.

Some issues to consider include:

1. Can the authors include information on the toxicity of PI3K -selective inhibitor BYL719 (alpelisib) in their model? Please also discuss toxicity of Lenvatinib and of the combination treatments tested in vivo (Lenvatinib + BYL719; Lenvatinib + anti-PD-1).

2. Fig. 2F: Can the authors provide a potential explanation for the transient effects of Lenvatinib in recurrent tumors?

3. Fig. 4e,f: Quantification of biological replicates should be performed to confirm conclusions, especially since some of the blots shown are overexposed.

4. Lenvatinib is used as an FGFR signaling inhibitor in all relevant experiments. Can the authors confirm that observed effects are predominantly due to FGFR inhibition (e.g., data in figures 5f-j)? Can inhibitory effects on additional Lenvatinib’s targets be excluded from playing a role?

Minor:
Line 126: missing text
Lines 141-144: The stated timing of tumor development (1 month) differs from 3-5 months in the first paragraph.
The labels on some of the figures (e.g., Figure 4a,b) are too small
Extended data 5b – please correct language

Reviewer #3

(Remarks to the Author)
Cheng et al. present a profound and well-written manuscript submitted for publication in Nature Communications. The work makes use of a novel, valuable GEMM of SEC to dissect the mechanism of resistance to the PI3Ka inhibitor alpelisib, and discovers FGFR gains as a driver mechanism. In order to produce a compelling manuscript ready for publication, the points delineated below should be addressed. In particular, the expansion to the use of FGFR-specific inhibitors is strongly advised.

1. In the introduction, the authors state that “several mechanisms of resistance to PIK3CA-targeted therapies have been proposed”. The manuscript introduction would benefit from exemplifying at least some of these resistance mechanisms in more detail rather than just citing a list of papers.

2. In the first results paragraph describing the SEC GEMM generation, the authors refer to “incorporating common genetic alterations observed in human SEC”. The authors should re-cite here the appropriate literature that they introduce in the introduction. Following that, the authors write: “This APM model was established by isolating uterine epithelial cells from FVB mice carrying three genetic elements: Rosa-lsl-rtTA, floxed-Trp53, and tetracycline (tet)-regulated (33)”. Shouldn’t it say (tet)-regulated PIK3CA-H1047R? Please update.

3. Fig 2c: The authors also observed upregulation of Fgf7 in RNAseq. This is worth mentioning in the manuscript text, given that Fgf7 may serve as ligand to the upregulated FGFRs.

4. Fig. 2f-g: Besides using lenvatinib, the authors should also use clinical FGFR-specific inhibitors, such as pemigatinib. The current data using a pan-RTK inhibitor are not strongly supporting the hypothesis that FGFRs are the causal resistance driver alterations.
Along those lines, the authors should provide causal confirmation that FGFRs can drive resistance to PI3Ki, first, by using the FGFR OE system that they introduce in Fig. 6c, and/or second, by using RNAi against the FGFRs to resensitize to PI3Ki. Moreover, besides phosphorylation of FRS2 and ERK, the authors should also evaluate the FGFRi impact on PI3K signaling (e.g., AKT, S6, etc.) as well as STAT3. Those signaling axes are known to mediated oncogenic FGFR pathway transduction besides MAPK. Speaking of, in Fig. 2b, do the p-AKT levels in resistant tumors represent reactivation of PI3K signaling due to FGFR? The levels seem definitely higher than the ones observed in Fig. 1g upon PI3K blockade (but before occurrence of resistance).

5. Fig. 3c: The authors should confirm the pre-existence of FGFR1/2 high and the absence of FGFR3 high epithelial cells in the primary tumors by using orthogonal, protein-level methods (for instance, IHC or FACS). This should be compared to resistant tumors.

6. Fig. 4b/d: The authors should perform full dose-titration curves rather than just showing few titration points, especially for the resistant models.

7. Fig. 4e/f: The authors should also evaluate phosphorylation of ERK (done in f), STAT3, 4EBP1, and EIF4B.

8. Fig. 4f-g: Also here, the manuscript would benefit from the use of an FGFR-specific inhibitor, such as pemigatinib.

9. Fig. 4g: Rather than using single doses, the authors may consider using full dose-titration matrixes to support these data. This should also be put into direct comparison with parental HEC-1-A. Is an additive (or synergistic) effect with PI3Ki + FGFRi also observed in PI3Ki-sensitive, parental cell lines?

10. Fig. 6d/e: Lenvatinib alone appears to have profound impact on the immune compartment. Can the authors rule out that lenvatinib directly impacts immune cells? The in vitro experiments (Fig. 5i/j) suggest that FGFRi indeed releases antigen presentation in a tumor cell-intrinsic manner. However, the tumor-intrinsic vs immune compartment impact of lenvatinib in vivo is less clear. The authors could deplete FGFRs in the tumor cells only and demonstrate that the impact on MHC-I presentation machinery and immune cells is akin to using systemic FGFR inhibitors. This would make a more compelling rationale for combining FGFRi with anti-PD1.

11. In Fig. 2c, the authors describe a subset of resistant tumors that gain expression of cytokines but are devoid of FGFR gains. Do those resistant tumors also benefit from anti-PD1? If not addressed functionally, this should at least be discussed in the results and/or discussion of the manuscript.

12. In general, the manuscript would benefit from expanding the used PI3Ki to the newest generation of PI3Ka inhibitors, such as inavolisib.

13. The authors should discuss in more detail and put their work into context of the current preclinical and clinical evidence for combining PI3Ki with FGFRi as well as FGFRi with ICB, whether actual cooperation of the combos has been observed, as well as the potential combo limitations and toxicities.

Version 1:

Reviewer comments:

Reviewer #1

(Remarks to the Author)
My questions have been fully addressed and paper is ready to be published.

Reviewer #2

(Remarks to the Author)
The authors have been highly responsive to the concerns raised by all three Reviewers, providing extensive new data and clarifying the text. All concerns raised by this Reviewer have been addressed. This is a strong study that will be of interest to the broad readership of Nature Communications.

Reviewer #3

(Remarks to the Author)
In the revised version of the manuscript, the authors addressed all the points raised.
One minor comment regarding Suppl. Fig. 4F: It should say PIK3CA*, not PI3KCA*

Open Access This Peer Review File is licensed under a Creative Commons Attribution 4.0 International License, which permits use, sharing, adaptation, distribution and reproduction in any medium or format, as long as you give appropriate credit to the original author(s) and the source, provide a link to the Creative Commons license, and indicate if changes were made.
In cases where reviewers are anonymous, credit should be given to 'Anonymous Referee' and the source.
The images or other third party material in this Peer Review File are included in the article's Creative Commons license, unless indicated otherwise in a credit line to the material. If material is not included in the article's Creative Commons license and your intended use is not permitted by statutory regulation or exceeds the permitted use, you will need to obtain permission directly from the copyright holder.
To view a copy of this license, visit https://creativecommons.org/licenses/by/4.0/

Point-by-point response to reviewers' comments

Title: Targeting FGFR signaling to overcome therapeutic resistance and immune evasion in oncogenic PIK3CA-driven serous endometrial cancer

Reviewer #1 (Remarks to the Author):

This study presents a clinically relevant, immunocompetent mouse model of serous endometrial cancer (SEC) that incorporates three commonly observed genetic alterations in human SEC: PIK3CA mutation, TP53 loss, and MYC overexpression. This model recapitulates pathological and molecular features of a subset of human SEC and provides a valuable platform to investigate mechanisms of therapeutic resistance and immune evasion. The authors demonstrate that SEC tumors initially depend on PIK3CA signaling for growth and survival, but frequently develop resistance through activation of FGFR pathways. Specifically, FGFR1 and FGFR2 are associated with intrinsic resistance, while FGFR3 is linked to acquired resistance following targeted therapy. Furthermore, elevated FGFR signaling promotes immune evasion by downregulating MHC-I and HLA gene expression, resulting in impaired antigen presentation and reduced T cell infiltration. The study shows that combined inhibition of PI3Kα (using BYL719) and FGFR (using Lenvatinib) effectively suppresses tumor growth in both primary and recurrent settings. Notably, FGFR blockade restores antigen presentation and enhances the therapeutic efficacy of anti-PD-1 immunotherapy, leading to durable immune responses in preclinical models. These findings highlight the dual role of FGFR signaling in mediating both therapeutic resistance and immune suppression, and support the rationale for combined targeted and immunotherapeutic strategies in the treatment of SEC.

1. While this model is an advancement on EC (SEC) model, several limitations should be addressed. One of the hallmark features of SEC is extensive genomic instability and widespread copy number alterations. Although TP53 loss is included in the model and might contribute to genomic instability, it remains unclear whether the model faithfully recapitulates the copy number alterations seen in human SEC. The authors should evaluate copy number variation in the model and compare the genomic landscape to human SEC tumors. If the model does not exhibit the high level of copy number alterations that is a hallmark of human SEC, it may not fully qualify as a representative SEC model, despite being a useful genetically engineered mouse model for endometrial cancer in general.

We appreciate your insightful comment regarding the SEC subtype, genomic instability and copy-number alterations in our models. Indeed, copy number variation (CNV) analysis of TCGA endometrial cancer samples shows that SEC exhibit a high fraction of genome altered (FGA; median = 0.41), consistent with extensive genomic instability and widespread copy number alterations (**Figure R1a**). In contrast, non-serous TCGA endometrial cancers display a much lower burden of copy number change (median FGA ≈ 0.07) (**Figure R1b**).

We performed the same CNV/FGA analysis on primary APM tumors and their derived recurrent tumors. The FGA values in APM tumors (harboring *PIK3CA* mutation, *Trp53* loss, and *Myc* overexpression) and its recurrent tumors (Rec 1 and Rec 9) are 0.3 and 0.61, respectively (**Figure R1c**), comparable to those observed in human SEC tumors (median FGA = 0.41) (**Figure R1a and c**). These findings indicate that our models faithfully recapitulate the high level of genomic instability

that is a defining hallmark of human SEC and place APM and its recurrent derivatives within the copy-number-high/serous-like molecular subgroup identified by TCGA. These data have been incorporated into the revised manuscript as **Supplementary Figure 1e**.

2. It would be beneficial to compare this model to other genetically engineered mouse models of endometrial cancer (there is several EC mouse models, although they are not specifically for SEC) to better define its specificity and relevance to the serous subtype.

We thank you for this valuable suggestion. Existing genetically engineered mouse models (GEMMs) of EC, including PTEN-loss, LKB1-loss, Mig-6 knockout, and p53-deficient systems, have been instrumental in elucidating the biology of endometrioid (Type I) EC and, to a lesser extent, some features of high-grade disease[1]. PTEN and LKB1 knockout models recapitulate estrogen-driven endometrioid hyperplasia and carcinoma, Mig-6-deficient mice highlight progesterone-estrogen imbalance in early EC pathogenesis, and p53 loss more closely approximates serous-like (Type II) disease and produces invasive tumors.

Among these, the PTEN-loss-driven EC model generated by Dr. Dolcet's group has been particularly well characterized in multiple studies and was classified as a Type I-like/endometrioid EC[2, 3, 4]. To directly benchmark our model, we performed RNA-seq-based GSEA comparing APM tumors with this PTEN-loss EC GEMM, using publicly available RNA-seq data (GSE225691) [4]. APM tumors showed strong enrichment of G2M checkpoint, DNA replication stress-associated programs, E2F targets, and MYC target pathways relative to the PTEN-loss model (**Figure R2a;** see also **Supplementary Figure 1d**), consistent with the latter representing an endometrioid-like EC and APM aligning with a serous-like (SEC) profile. Notably, the PTEN-loss model predominantly manifests as endometrial in situ adenocarcinoma, whereas APM forms advanced invasive tumors, further underscoring biological differences between the two systems.

REDACTED: Additional experimental model data that were not included in the revised manuscript have been removed from this response letter.

Together, these comparisons indicate that, whereas existing EC GEMMs (including Dolcet's PTEN-loss and our KP model) primarily model endometrioid/Type I-like disease, the APM model-generated in an immunocompetent background and integrating multiple co-occurring genetic drivers-faithfully reflects the complex, multi-driver biology of human SEC. In addition to extensive genomic instability and widespread copy-number alterations (see response to Comment #1), pathway-level transcriptional signatures align APM with the TCGA copy-number-high/serous-like molecular subgroup. Thus, APM and its recurrent derivatives provide a specific and biologically relevant system for studying serous-like EC, its therapeutic vulnerabilities, and mechanisms of resistance and immune evasion, while complementing existing Type I-biased EC GEMMs.

Because the primary focus of this manuscript is the establishment and characterization of the APM model, and the KP model is still under characterization, we feel it would be premature to include KP-derived data as main or supplementary figures. Accordingly, **Figure R2b-d** is provided here solely to support our response to the reviewer and is not included in the revised manuscript.

3. The study also employs ten human endometrial cancer cell lines to assess sensitivity to the PI3Kα inhibitor BYL719. However, the histological or molecular subtypes of these cell lines are not clearly defined. Are they all SEC lines? Since different subtypes of endometrial cancer, such as endometrioid and serous, exhibit distinct biological behaviors and drug responses, it is important for the authors to classify the cell lines used in the study to determine their relevance to serous endometrial cancer specifically.

We appreciate this important point. In the revised manuscript, we now provide the mutational profiles and histologic/molecular subtypes for all EC cell lines used in this study (**Figure R3a**; see also **Supplementary Fig. 4a**). These annotations, derived from published pathology reports and publicly available genomic datasets, indicate that our panel includes both endometrioid and non-endometrioid/serous-like lines, rather than being restricted exclusively to SEC models. Human SEC cell lines are relatively limited; ARK1 is the only bona fide SEC line in our panel that harbors a PIK3CA mutation, and it is included in all relevant analyses.

Across the full panel, IC_{50} analyses show that higher FGFR expression is associated with reduced sensitivity to both BYL719 (alpelisib) and the next-generation PI3Kα inhibitor GDC-0077 (inavolisib), indicating that this relationship is not restricted to a single EC subtype (**Figure R3b, c**). Notably, 9 of the 10 EC cell lines in our panel harbor TP53 mutations, a defining molecular feature of the TCGA copy-number–high / serous-like subtype, which is highly enriched in serous endometrial carcinoma. This molecular profile further supports the relevance of our cell line panel to the APM model and to serous-like EC biology. Among *PIK3CA*-mutant EC cell lines, responsiveness to BYL719 is better explained by FGFR expression levels than by histologic subtype: $FGFR^{high}$ lines, irrespective of whether they are endometrioid or serous-like, exhibit relative resistance, whereas $FGFR^{low}$ lines remain sensitive. Together, these findings support FGFR-driven modulation of PI3Kα inhibitor response as a largely subtype-agnostic resistance mechanism in EC, with our in vivo APM model and the ARK1 SEC line specifically anchoring this biology in the serous-like context.

4. In the analysis of TCGA endometrial cancer patient data, the authors report an inverse correlation between FGFR expression and CD3D expression, suggesting reduced T cell infiltration in FGFR-high tumors. While this is a useful observation, the authors should apply immune deconvolution tools such as CIBERSORT to more accurately assess the composition of immune cell subsets within the bulk tumor transcriptomic data. Importantly, how about other immune cell populations, and whether they show similar as the model developed by this study.

We thank you for this constructive suggestion. In response, we performed CIBERSORT-based immune deconvolution of the TCGA EC transcriptomic dataset to more comprehensively characterize immune cell subsets in relation to FGFR expression. This analysis revealed that FGFR-high tumors harbor significantly reduced $CD8^+$ T-cell and an increased proportion of M2 macrophages, consistent with a more immunosuppressive tumor microenvironment in FGFR-activated tumors (**Figure R4a, b**; see also **Supplementary Fig. 5a**). We further examined other immune cell populations across FGFR

expression levels. While changes in some subsets were less pronounced, FGFR-high tumors showed a overall shift toward a less inflamed landscape, characterized by reduced effector T-cell signatures and a myeloid compartment skewed toward M2/TAM-like phenotypes (**Figure R4a, b**; see also **Supplementary Fig. 5a**).

Importantly, these immune deconvolution results closely parallel the immune landscape observed in our preclinical models. In both the recurrent Rec1 tumors with spontaneous FGFR overexpression (**Figure R4c**; see also **Fig. 5d**) and primary tumors engineered to overexpress FGFRs (**Figure R4d**; see also **Fig. 5e**), we observe reduced T-cell infiltration and an increased proportion of M2-type TAMs by flow cytometry. Together, these data indicate that elevated FGFR signaling drives a conserved immunosuppressive shift in both human EC and the APM model, closely aligning the immune landscape of our preclinical system with that observed in patient tumors.

5. The single-cell RNA sequencing analysis in this study reveals important insights into the heterogeneity of tumor and immune cell populations, but the immune profiling remains relatively superficial. More detailed characterization of immune cell subtypes, particularly among T cells (such as effector, memory, regulatory) and myeloid cells (including macrophage and dendritic cell subsets), would provide a clearer picture of the immune landscape in primary and recurrent tumors. This deeper resolution would help elucidate how FGFR signaling reshapes the tumor microenvironment and contributes to immune escape.

We appreciate this excellent suggestion. In response, we reanalyzed the the single-cell RNA-seq dataset with a focus on achieving greater granularity in immune profiling and updated the immune cell annotations accordingly. Specifically, we reclustered T cells, NK cells, and myeloid subsets, including M1- and M2-like macrophages as well as dendritic cell-enriched clusters (**Figure R5a**; see also **Supplementary Fig. 3d**).

The more detailed analysis revealed that FGFR-high recurrent tumors (Rec1) exhibited an increase in M2-TAMs and a concomitant reduction in M1-TAMs and total T-cell infiltration relative to primary tumors (APM) (**Figure R5b**; see also **Supplementary Fig. 3e**). Winthin the T-cell compartment, we observe a marked depletion of cytotoxic $CD8^{+}$ T cells, accompanied by a relative enrichment of exhausted T cells and regulatory T cells (Tregs), immune subsets commonly associated with immunosuppression and T-cell dysfunction (**Figure R5c**; see also **Supplementary Fig. 3f**). Together, these analyses provide a more detailed picture of the immune landscape in primary versus FGFR-high recurrent tumors and demonstrate that FGFR activation is associated with a coordinated shift toward an immunosuppressive tumor microenvironment-characterized by M2-dominant TAMs, loss of cytotoxic $CD8^{+}$ T cells, and expansion of Tregs-which provides mechanistic insight into how FGFR signaling may promote immune escape and tumor recurrence.

6. Another issue is the use of lenvatinib as the FGFR inhibitor in the study. Lenvatinib is a broad-spectrum tyrosine kinase inhibitor that targets not only FGFR1-4 but also VEGFR1-3, RET, KIT, and others. As a result, it is difficult to attribute the observed therapeutic effects solely to FGFR inhibition. To strengthen the mechanistic conclusions, the authors should evaluate more selective FGFR inhibitors, such as erdafitinib or pemigatinib, which would provide cleaner evidence of FGFR-specific contributions to resistance and immune evasion.

We appreciate this important point and agree that the broad target profile of lenvatinib can complicate

mechanistic interpretation. We initially selected lenvatinib because it is the clinically approved partner with PD-1 blockade in advanced EC. In response to your suggestion, we performed additional experiments using pemigatinib, a more FGFR-selective inhibitor that is approved for FGFR-driven cancers[5].

Specifically, we carried out the following studies:

(1) **FGFR-dependent tumor growth and signaling in FGFR-high recurrent tumors:** Pemigatinib significantly suppressed the growth of Rec1 tumors, a recurrent model that arises after *PIK3CA*-H1047R inactivation and overexpresses FGFR1-3 (**Figure R6a**; see also **Fig. 2h**). This growth inhibition was accompanied by reduced phosphorylation of FRS2 and ERK in tumors (**Figure 6b**; see also **Fig. 2i**). These effects closely mirror those observed with lenvatinib (**Figure R6c,d**; see also **Fig. 2f, g**), indicating on-target blockade of FGFR signaling. Together, these data demonstrate that inhibition of FGFR signaling is sufficient to impair growth of FGFR-high recurrent tumors, supporting a functional dependence on FGFR signaling in this setting.

(2) **Restoration of PI3Kα inhibitor sensitivity in FGFR-high, *PIK3CA*-mutant human EC models:** We next evaluated combinations of pemigatinib with BYL719 (alpelisib) or GDC-0077 (inavolisib) in FGFR-overexpressing, *PIK3CA*-mutant EC cell lines, including HEC-1-AR (a PI3Kαi-resistant line generated by continuous, dose-escalating BYL719 exposure that acquired FGFR1-3 overexpression) and SNG-M (a de novo PI3Kα inhibitor-resistant, FGFR-high line). In HEC-1-AR cells, pemigatinib restored PI3Kα pathway suppression when combined with BYL719 or GDC-0077 and produced additive to synergistic effects on cell viability, as quantified by Combenefit (**Figure R7a, b**; see also **Fig. 4f, g**). In SNG-M xenografts, BYL719 alone had minimal activity and pemigatinib alone produced only modest tumor growth delay, whereas the combination resulted in significantly greater tumor growth inhibition, comparable to BYL719 plus lenvatinib (**Figure R7c**; see also **Supplementary Fig. 4j**). These findings demonstrate that selective FGFR blockade with pemigatinib is sufficient to reverse PI3Kα inhibitor resistance in FGFR-high models, indicating that the effects observed with lenvatinib indeed reflect FGFR-specific modulation rather than off-target kinases inhibition.

(3) **FGFR-specific regulation of antigen presentation and myeloid/T-cell composition in FGFR-high EC models:** To interrogate FGFR-specific contributions to immune evasion, we performed in vitro studies in FGFR-high human EC models (EN cells with FGFR1-3 overexpression and PDO DF4203 harboring FGFR1 amplification) and in vivo analyses in the syngeneic Rec1 allograft model. In EN cells and DF4203 organoids, both lenvatinib and pemigatinib induced comparable upregulation of genes involved in antigen processing and presentation (**Figure R8a, b**; see also **Fig. 5j, k**). Consistently, in Rec1 tumors, pemigatinib treatment or genetic FGFR knockdown induced antigen-presentation gene signatures similar to those observed with lenvatinib (**Figure R8c, d**; see also **Supplementary Fig. 5c and Fig. 5f**). At the tumor microenvironment level, pemigatinib increased total and $CD8^+$ T-cell infiltration while reducing M2-type TAMs, closely mirroring the immune effects of lenvatinib (**Figure R8c, e**; see also **Supplementary Fig. 5c and Fig. 5f**). Thses data indicate that enhanced antigen presentation and immune remodeling are direct consequence of FGFR inhibition.

(4) **Enhancement of antitumor immunity and response to checkpoint blockade:** Finally, in the syngeneic Rec1 model, pemigatinib enhanced antitumor immunity and augmented the efficacy

of anti-PD-1 therapy, similar to the effects observed with lenvatinib (**Figure R9a;** see also **Supplementary Fig. 6b**). Analysis of tumor-infiltrating lymphocytes and draining lymph nodes (LN) showed increased frequencies of $CD3^+$ and $CD8^+$ T cells among $CD45^+$ cells, as well as higher proportions of TNFα- and IFNγ-producing $CD8^+$ T cells in the pemigatinib or pemigatinib plus anti-PD-1 treatment groups (**Figure R9b, c**; see also **Supplementary Fig. 6f, g**). These results provide functional evidence that selective FGFR inhibition remodels the immune microenvironment and enhances responsiveness to immunotherapy in FGFR-high recurrent tumors.

Together, these experiments using a highly selective FGFR inhibitor, along with genetic FGFR knockdown, clearly demonstrate that the key therapeutic and immunomodulatory effects observed in our studies are mediated by specific inhibition of FGFR signaling rather than off-target effects of lenvatinib. This strengthens the mechanistic link between FGFR activation, PI3Kα inhibitor resistance, and immune evasion.

7. While the study shows that FGFR overexpression suppresses antigen presentation and promotes immune escape, the precise downstream signaling mechanisms responsible for this effect remain poorly defined. It would be informative to dissect which downstream pathways, such as MAPK, PI3K-AKT, or STAT signaling, mediate the immunosuppressive effects of FGFR activity. Understanding these mechanisms could further guide rational design of combination therapies.

We appreciate this important mechanistic question. To identify the key downstream mediators of FGFR-driven immune suppression, we systematically interrogated major signaling pathways downstream of FGFR activation, including MAPK-ERK, STAT3, and PI3K-AKT signaling. Pharmacologic inhibition of the MAPK-ERK pathway most closely recapitulated the immunomodulatory effects of FGFR blockade, leading to increased MHC-I expression on tumor cells both in vitro and in vivo in Rec 1 tumor allografts, together with a reduction M2-type TAMs in Rec1 tumor model (**Figure R10a, b**; see also **Supplementary Fig. 5 f, i**). In contrast, inhibition of STAT3 or PI3K signaling did not produced comparable changes in antigen-presentation gene expression (**Figure R10a**; see also **Supplementary Fig. 5 g, h**).

Moreover, combined inhibition of MAPK and PI3K signaling markedly restored sensitivity to BYL719 in PI3Kα inhibitor-resistant APM-R tumors (generated by long-term BYL719 dose escalation and are characterized by acquired FGFR overexpression; Supplementary Fig. 2h) (**Figure R10c**; see also **Supplementary Fig. 5j**). Notably, mice maintained body weight during combined MAPK inhibitor plus BYL719 treatment, suggesting that this regimen was well tolerated in vivo. Together, these results delineate MAPK-ERK signaling as a key downstream mediator of the immunosuppressive and resistance-promoting effects of FGFR activation and provide a mechanistic rationale for combination strategies that incorporate PI3Kα and MAPK pathway inhibition alongside FGFR-targeted therapy.

Reviewer #2 (Remarks to the Author):

In their manuscript entitled “Targeting FGFR signaling overcomes therapeutic resistance and immune evasion in oncogenic PIK3CA-driven serous endometrial cancer,” Cheng et al. leverage a novel, clinically relevant, immunocompetent model of serous endometrial cancer (SEC) driven by PIK3CA mutation (PIK3CA-H1047R), TP53 loss, and c-MYC overexpression to explore mechanisms of resistance to PIK3CA therapy. In this model, FVB mouse uterine epithelial cells carrying Rosa-lsl-rtTA,

floxed-Trp53, and tetracycline (tet)-regulated mutant PIK3CA were further modified with adenoviral Cre and lentiviral mouse Myc, and orthotopically transplanted into the uterine wall of FVB female recipients. Using single cell transcriptional profiling, the authors determined that FGFR1/2 signaling is associated with intrinsic resistance to PIK3CA targeted therapies. FGFR3, in turn, was linked to acquired resistance to these agents. Combined inhibition of FGFR and PI3Kα enhanced tumor suppression in this model. Further analysis of the downstream effects of FGFR signaling identified a role for this pathway in immune evasion via suppression of antigen-processing and presentation; FGFR inhibition reversed the effect and synergized with anti-PD-1 therapy. The data are generally of high quality and supportive of the conclusions of the authors. Together, these studies provide strong support for FGFR inhibition as a strategy for the treatment of a subset of patients with SEC. This a compelling study with high significance and should be of interest to the broad readership of Nature Communications.

We are grateful for your positive and invaluable comments, which are highly beneficial for deepening and expanding our research. We have carefully reviewed the comments and addressed the concerns below.

Some issues to consider include:

1. Can the authors include information on the toxicity of PI3Ka-selective inhibitor BYL719 (alpelisib) in their model? Please also discuss toxicity of Lenvatinib and of the combination treatments tested in vivo (Lenvatinib + BYL719; Lenvatinib + anti-PD-1).

We appreciate your comment and fully agree that toxicity is an important consideration, particularly for agents such as alpelisib, lenvatinib, and anti-PD-1, all of which have well-described clinical adverse events. In patients, alpelisib (BYL719) is most commonly associated with hyperglycemia, rash, and diarrhea; lenvatinib with hypertension, fatigue, and stomatitis; and anti-PD-1 antibodies with immune-related adverse events (irAEs). Although our preclinical study was not designed as a formal toxicology assessment, we prospectively monitored the tolerability of the PI3Kα-selective inhibitor BYL719, FGFR inhibitors, and all combination regimens in our in vivo models.

To evaluate potential toxicity, animals were closely monitored throughout the study (Page 15, Lines 563-564). Serial body weight measurements showed no significant weight loss with PIK3a-selective inhibitor monotherapy (**Figure R11a**; see also **Supplementary Fig. 1i, g**), with combinations of BYL719 plus lenvatinib or pemigatinib (**Figure R11b**; see also **Supplementary Fig. 4j, k**), with GSK1120212 plus BYL719 (**Figure R11c**; see also **Supplementary Fig. 5j**), or with lenvatinib or pemigatinib plus anti-PD-1 (**Figure R11d**; see also **Supplementary Fig. 6a, b**). In parallel, routine health monitoring during treatment revealed no signs of drug-related toxicity, including no reduced activity or lethargy, no loss of appetite, no ruffled or unkempt fur, no dehydration or diarrhea, and no mortality or early euthanasia.

Together, these findings indicate that BYL719, lenvatinib, and the various combination regimens tested were well tolerated under the dosing schedules used in our in vivo experiments. We have now explicitly incorporated these preclinical tolerability data into the revised manuscript, while also acknowledging the distinct toxicity profiles of alpelisib and Lenvatinib observed clinically and the importance of careful dose optimization and monitoring in future translational studies.

2. Fig. 2F: Can the authors provide a potential explanation for the transient effects of Lenvatinib in recurrent tumors?

We appreciate your insightful question. The transient effects of lenvatinib observed in Rec1 tumors are most likely attributable to the emergence of adaptive resistance mechanisms, a common consequences of targeted therapies oberserved in both clinical and preclinical settings[6].

To investigate this possibility in our system, we generated a lenvatinib-resistant (Len-R) model by transplanting Rec1 tumor allografts at the time they became refractory to continuous lenvatinib treatment, followed by two additional passages under sustained lenvatinib exposure (**Figure R12a**, see also **Fig. 2f**). These transplanted tumors no longer responded to lenvatinib, confirming the establishment of Len-R tumors (**Fig. R12a**). Whereas previous studies have reported lenvatinib resistance mechanisms involving reactivation of downstream or parallel signaling pathways and epithelial-mesenchymal transition (EMT)[7, 8], our Len-R tumors did not show significant changes in STAT3, ERK, PI3K-AKT, c-MET, or EMT pathway activation compared with parental Rec1 tumors (**Fig. R12b, c**). These findings suggest that additional or alternative resistance mechanisms, beyond canonical pathway reactivation, may contribute to the transient antitumor effects of lenvatinib in our setting.

In addition, prior studies have demonstrated that lenvatinib exerts complex and dynamic effects on the tumor microenvironment. Although lenvatinib can initially reduce M2-type TAMs, this effect is often not durable, with re-emergence of an immunosuppressive milieu characterized by increased M2 macrophages infiltration[9]. Consistent with these observations, we observed a significant increase in M2-type TAMs in Len-R tumors compared with parental Rec1 tumors (**Fig. R12d**), supporting a role for microenvironmental remodeling in the loss of therapeutic efficacy.

Together, these results indicate that the transient response to lenvatinib reflects a combination of adaptive tumor-intrinsic and microenvironmental changes, rather than sustained inhibition of FGFR signaling alone. This observation further underscores the rationale for combination therapeutic strategies targeting both FGFR signaling and the tumor immune microenvironment to achieve more durable antitumor responses.

3. Fig. 4e,f: Quantification of biological replicates should be performed to confirm conclusions, especially since some of the blots shown are overexposed.

We thank you for this valuable comment. To address this point, we have repeated the Western blot experiments shown in Fig. 4e and f using three independent biological replicates under non-saturating exposure conditions. Densitometric quantification has now been included, confirming the reproducibility and statistical robustness of the findings (**Figure R13**; updated **Fig. 4e, f**). All uncropped blots and raw quantification data have been provided in the Source Data file.

4. Lenvatinib is used as an FGFR signaling inhibitor in all relevant experiments. Can the authors confirm that observed effects are predominantly due to FGFR inhibition (e.g., data in figures 5f-j)? Can inhibitory effects on additional Lenvatinib's targets be excluded from playing a role?

We appreciate this important point and agree that the broad target profile of lenvatinib can complicate mechanistic interpretation. We initially selected lenvatinib because it is the clinically approved partner

with PD-1 blockade in advanced EC. In response to your suggestion, we performed additional experiments using pemigatinib, a more FGFR-selective inhibitor that is approved for FGFR-driven cancers[5].

Specifically, we carried out the following studies:

(1) **FGFR-dependent tumor growth and signaling in FGFR-high recurrent tumors:** Pemigatinib significantly suppressed the growth of Rec1 tumors, a recurrent model that arises after *PIK3CA*-H1047R inactivation and overexpresses FGFR1-3 (**Figure R6a**; see also **Fig. 2h)**. This growth inhibition was accompanied by reduced phosphorylation of FRS2 and ERK in tumors (**Figure 6b**; see also **Fig. 2i**). These effects closely mirror those observed with lenvatinib (**Figure R6c,d**; see also **Fig. 2f, g**), indicating on-target blockade of FGFR signaling. Together, these data demonstrate that inhibition of FGFR signaling is sufficient to impair growth of FGFR-high recurrent tumors, supporting a functional dependence on FGFR signaling in this setting.

(2) **Restoration of PI3Kα inhibitor sensitivity in FGFR-high, *PIK3CA*-mutant human EC models:** We next evaluated combinations of pemigatinib with BYL719 (alpelisib) or GDC-0077 (inavolisib) in FGFR-overexpressing, *PIK3CA*-mutant EC cell lines, including HEC-1-AR (a PI3Kαi-resistant line generated by continuous, dose-escalating BYL719 exposure that acquired FGFR1-3 overexpression) and SNG-M (a de novo PI3Kα inhibitor-resistant, FGFR-high line). In HEC-1-AR cells, pemigatinib restored PI3Kα pathway suppression when combined with BYL719 or GDC-0077 and produced additive to synergistic effects on cell viability, as quantified by Combenefit (**Figure R7a, b**; see also **Fig. 4f, g**). In SNG-M xenografts, BYL719 alone had minimal activity and pemigatinib alone produced only modest tumor growth delay, whereas the combination resulted in significantly greater tumor growth inhibition, comparable to BYL719 plus lenvatinib (**Figure R7c**; see also **Supplementary Fig. 4j**). These findings demonstrate that selective FGFR blockade with pemigatinib is sufficient to reverse PI3Kα inhibitor resistance in FGFR-high models, indicating that the effects observed with lenvatinib indeed reflect FGFR-specific modulation rather than off-target kinases inhibition.

(3) **FGFR-specific regulation of antigen presentation and myeloid/T-cell composition in FGFR-high EC models:** To interrogate FGFR-specific contributions to immune evasion, we performed in vitro studies in FGFR-high human EC models (EN cells with FGFR1-3 overexpression and PDO DF4203 harboring FGFR1 amplification) and in vivo analyses in the syngeneic Rec1 allograft model. In EN cells and DF4203 organoids, both lenvatinib and pemigatinib induced comparable upregulation of genes involved in antigen processing and presentation (**Figure R8a, b**; see also **Fig. 5j, k**). Consistently, in Rec1 tumors, pemigatinib treatment or genetic FGFR knockdown induced antigen-presentation gene signatures similar to those observed with lenvatinib (**Figure R8c, d**; see also **Supplementary Fig. 5c and Fig. 5f**). At the tumor microenvironment level, pemigatinib increased total and $CD8^+$ T-cell infiltration while reducing M2-type TAMs, closely mirroring the immune effects of lenvatinib (**Figure R8c, e**; see also **Supplementary Fig. 5c and Fig. 5f**). Thses data indicate that enhanced antigen presentation and immune remodeling are direct consequence of FGFR inhibition.

(4) **Enhancement of antitumor immunity and response to checkpoint blockade:** Finally, in the syngeneic Rec1 model, pemigatinib enhanced antitumor immunity and augmented the efficacy of anti-PD-1 therapy, similar to the effects observed with lenvatinib (**Figure R9a;** see also

Supplementary Fig. 6b). Analysis of tumor-infiltrating lymphocytes and draining lymph nodes (LN) showed increased frequencies of $CD3^+$ and $CD8^+$ T cells among $CD45^+$ cells, as well as higher proportions of TNFα- and IFNγ-producing $CD8^+$ T cells in the pemigatinib or pemigatinib plus anti-PD-1 treatment groups (**Figure R9b, c**; see also **Supplementary Fig. 6f, g**). These results provide functional evidence that selective FGFR inhibition remodels the immune microenvironment and enhances responsiveness to immunotherapy in FGFR-high recurrent tumors.

Together, these experiments using a highly selective FGFR inhibitor, along with genetic FGFR knockdown, clearly demonstrate that the key therapeutic and immunomodulatory effects observed in our studies are mediated by specific inhibition of FGFR signaling rather than off-target effects of lenvatinib. This strengthens the mechanistic link between FGFR activation, PI3Kα inhibitor resistance, and immune evasion.

Minor:

1. Line 126: missing text

   We have revised the text to: "This APM model was established by isolating uterine epithelial cells from FVB mice carrying three genetic elements: Rosa-lsl-rtTA, floxed-Trp53, and tetracycline (tet)-inducible $PIK3CA^{H1047R}$-IRES-luciferase transgene (Page 4, Line 134)."

2. Lines 141-144: The stated timing of tumor development (1 month) differs from 3-5 months in the first paragraph.

   We thank you for highlighting this point of clarification. The timelines described in the manuscript refer to two distinct stages of model establishment.

   First, *de novo* primary APM tumors were generated from uterine epithelial cells isolated from compound genetically enginerred mouse model (GEMM) on an FVB background carrying Rosa-lsl-rtTA, floxed-Trp53, and a tet-inducible $PIK3CA^{H1047R}$-IRES-Luciferase transgene. These cells were treated in vitro with adenoviral Cre to induce Trp53 deletion and rtTA activation, transduced with lentiviral mouse Myc, and orthotopically transplanted into the uterus of syngenic FVB female mice. In this primary setting, tumors developed over 3-5 months with full penetrance.

   To create a more time-efficient and experimentally tractable system for mechanistic and therapeutic studies, primary tumor cells derived from these *de novo* APM tumors were subsequently orthotopically injected into the uterine wall of secondary syngenic FVB recipient mice. In this secondary transplantation setting, doxycycline-induced $PIK3CA^{H1047R}$ expression led to rapid tumor formation within approximately one month in all recipients.

   We have clarified this two-step process and the corresponding timelines in the revised manuscript (Page 4, Lines 132-138 and Page 5, Lines 161-165).

3. The labels on some of the figures (e.g., Figure 4a,b) are too small

   The font sizes and labels in the affected figures (e.g., Fig. 4a-d) have been enlarged in the revised version to improved clarity and readability.

4. Extended data 5b - please correct language

We have revised the language in Supplementary Fig. 5b for clarity. The updated text now reads: "(c, d) Upregulation of antigen processing and presentation-related genes and reduction of M2-type TAMs in recurrent tumors following lenvatinib or pemigatinib treatment (n = 8-10 per group)."

Reviewer #3 (Remarks to the Author):

Cheng et al. present a profound and well-written manuscript submitted for publication in Nature Communications. The work makes use of a novel, valuable GEMM of SEC to dissect the mechanism of resistance to the PI3Ka inhibitor alpelisib, and discovers FGFR gains as a driver mechanism. In order to produce a compelling manuscript ready for publication, the points delineated below should be addressed. In particular, the expansion to the use of FGFR-specific inhibitors is strongly advised.

We greatly appreciate your positive feedback and constructive suggestions. In particular, we fully agree with the importance of using FGFR-selective inhibitors to strengthen the mechanistic conclusions. In response, we have incorporated a selective FGFR1-3 inhibitor (pemigatinib) into all key experiments, including tumor growth assays, PI3Kα inhibitor resistance studies, antigen presentation analyses, and immunotherapy combination experiments. These additional studies rigorously validate that the observed effects on therapeutic resistance and immune evasion are specifically mediated by FGFR signaling.

1. In the introduction, the authors state that "several mechanisms of resistance to PIK3CA-targeted therapies have been proposed". The manuscript introduction would benefit from exemplifying at least some of these resistance mechanisms in more detail rather than just citing a list of papers.

Thank you for this helpful suggestion. In the revised manuscript, we have expanded the introduction to provide more detailed examples of resistance mechanisms to PIK3CA-targeted therapies. Specifically, we now state: "Several mechanisms of resistance to PIK3CA-targeted therapies have been reported, including feedback activation of AXL/EGFR/PKC signaling, compensatory activation of PI3Kβ in PTEN-deficient tumors, and reactivation of parallel MAPK and mTORC1 pathways[10, 11, 12, 13]. Additional studies have revealed metabolic and kinase rewiring, involving PIM1 upregulation and insulin/IGF feedback loops, which restore AKT-mTOR signaling and promote tumor survival despite PI3K inhibition[14, 15]." These revisions are reflected in the revised manuscript (Page 3, Lines 107-112).

2. In the first results paragraph describing the SEC GEMM generation, the authors refer to "incorporating common genetic alterations observed in human SEC". The authors should re-cite here the appropriate literature that they introduce in the introduction. Following that, the authors write: "This APM model was established by isolating uterine epithelial cells from FVB mice carrying three genetic elements: Rosa-lsl-rtTA, floxed-Trp53, and tetracycline (tet)-regulated (33)". Shouldn't it say (tet)-regulated PIK3CA-H1047R? Please update.

We have updated the text to clarify the description of the APM model. The revised text now reads: "Rosa-lsl-rtTA, floxed-Trp53, and a tetracycline (tet)-regulated PIK3CA-H1047R-IRES-luciferase

transgene". In addition, we have re-cited the relevant literature in this section to explicitly reference the common genetic alterations observed in human SEC, consistent with those described in the Introduction (Page 4, Lines 132 and 134).

3. Fig 2c: The authors also observed upregulation of Fgf7 in RNAseq. This is worth mentioning in the manuscript text, given that Fgf7 may serve as ligand to the upregulated FGFRs.

Thank you for this valuable suggestion. In the revised manuscript, we now highlight the upregulation of Fgf7 in the main text (Page 6, Line 199), noting that it may act as a ligand for the upregulated FGFRs. In addition, we have emphasized Fgf7 in Fig. 2c to make this point clearer.

4. Fig. 2f-g: Besides using lenvatinib, the authors should also use clinical FGFR-specific inhibitors, such as pemigatinib. The current data using a pan-RTK inhibitor are not strongly supporting the hypothesis that FGFRs are the causal resistance driver alterations.

We appreciate this important point and agree that the broad target profile of lenvatinib can complicate mechanistic interpretation. We initially selected lenvatinib because it is the clinically approved partner with PD-1 blockade in advanced EC. In response to your suggestion, we performed additional experiments using pemigatinib, a more FGFR-selective inhibitor that is approved for FGFR-driven cancers[5].

Specifically, we carried out the following studies:

(1) **FGFR-dependent tumor growth and signaling in FGFR-high recurrent tumors:** Pemigatinib significantly suppressed the growth of Rec1 tumors, a recurrent model that arises after *PIK3CA*-H1047R inactivation and overexpresses FGFR1-3 (**Figure R6a**; see also **Fig. 2h**). This growth inhibition was accompanied by reduced phosphorylation of FRS2 and ERK in tumors (**Figure 6b**; see also **Fig. 2i**). These effects closely mirror those observed with lenvatinib (**Figure R6c,d**; see also **Fig. 2f, g**), indicating on-target blockade of FGFR signaling. Together, these data demonstrate that inhibition of FGFR signaling is sufficient to impair growth of FGFR-high recurrent tumors, supporting a functional dependence on FGFR signaling in this setting.

(2) **Restoration of PI3Kα inhibitor sensitivity in FGFR-high, *PIK3CA*-mutant human EC models:** We next evaluated combinations of pemigatinib with BYL719 (alpelisib) or GDC-0077 (inavolisib) in FGFR-overexpressing, *PIK3CA*-mutant EC cell lines, including HEC-1-AR (a PI3Kαi-resistant line generated by continuous, dose-escalating BYL719 exposure that acquired FGFR1-3 overexpression) and SNG-M (a de novo PI3Kα inhibitor-resistant, FGFR-high line). In HEC-1-AR cells, pemigatinib restored PI3Kα pathway suppression when combined with BYL719 or GDC-0077 and produced additive to synergistic effects on cell viability, as quantified by Combenefit (**Figure R7a, b**; see also **Fig. 4f, g**). In SNG-M xenografts, BYL719 alone had minimal activity and pemigatinib alone produced only modest tumor growth delay, whereas the combination resulted in significantly greater tumor growth inhibition, comparable to BYL719 plus lenvatinib (**Figure R7c**; see also **Supplementary Fig. 4j**). These findings demonstrate that selective FGFR blockade with pemigatinib is sufficient to reverse PI3Kα inhibitor resistance in FGFR-high models, indicating that the effects observed with lenvatinib indeed reflect FGFR-specific modulation rather than off-target kinases inhibition.

(3) **FGFR-specific regulation of antigen presentation and myeloid/T-cell composition in FGFR-high EC models:** To interrogate FGFR-specific contributions to immune evasion, we performed in vitro studies in FGFR-high human EC models (EN cells with FGFR1-3 overexpression and PDO DF4203 harboring FGFR1 amplification) and in vivo analyses in the syngeneic Rec1 allograft model. In EN cells and DF4203 organoids, both lenvatinib and pemigatinib induced comparable upregulation of genes involved in antigen processing and presentation (**Figure R8a, b**; see also **Fig. 5j, k**). Consistently, in Rec1 tumors, pemigatinib treatment or genetic FGFR knockdown induced antigen-presentation gene signatures similar to those observed with lenvatinib (**Figure R8c, d**; see also **Supplementary Fig. 5c and Fig. 5f**). At the tumor microenvironment level, pemigatinib increased total and $CD8^+$ T-cell infiltration while reducing M2-type TAMs, closely mirroring the immune effects of lenvatinib (**Figure R8c, e**; see also **Supplementary Fig. 5c and Fig. 5f**). Thses data indicate that enhanced antigen presentation and immune remodeling are direct consequence of FGFR inhibition.

(4) **Enhancement of antitumor immunity and response to checkpoint blockade:** Finally, in the syngeneic Rec1 model, pemigatinib enhanced antitumor immunity and augmented the efficacy of anti-PD-1 therapy, similar to the effects observed with lenvatinib (**Figure R9a;** see also **Supplementary Fig. 6b**). Analysis of tumor-infiltrating lymphocytes and draining lymph nodes (LN) showed increased frequencies of $CD3^+$ and $CD8^+$ T cells among $CD45^+$ cells, as well as higher proportions of TNFα- and IFNγ-producing $CD8^+$ T cells in the pemigatinib or pemigatinib plus anti-PD-1 treatment groups (**Figure R9b, c**; see also **Supplementary Fig. 6f, g**). These results provide functional evidence that selective FGFR inhibition remodels the immune microenvironment and enhances responsiveness to immunotherapy in FGFR-high recurrent tumors.

Together, these experiments using a highly selective FGFR inhibitor, along with genetic FGFR knockdown, clearly demonstrate that the key therapeutic and immunomodulatory effects observed in our studies are mediated by specific inhibition of FGFR signaling rather than off-target effects of lenvatinib. This strengthens the mechanistic link between FGFR activation, PI3Kα inhibitor resistance, and immune evasion.

Along those lines, the authors should provide causal confirmation that FGFRs can drive resistance to PI3Ki, first, by using the FGFR OE system that they introduce in Fig. 6c, and/or second, by using RNAi against the FGFRs to resensitize to PI3Ki.

We appreciate this excellent suggestion and agree that causal evidence is needed to firmly link FGFR activity to PI3Kα inhibitor resistance. In response, we performed additional gain- and loss-of-function experiments using both FGFR overexpression and RNAi-mediated knockdown, and integrated these new data with our existing results in **Figure R14**.

First, we examined the relationship between endogenous FGFR levels and PI3Kα inhibitor sensitivity across a panel of human EC cell lines. BYL719 IC_{50} values inversely correlated with FGFR1-3 expression, with FGFR-high lines (e.g., EN, SNG-M) clustering among the least sensitive to BYL719 (**Figure 14a**; see also **Fig. 4a**). We then generated matched BYL719-resistant derivatives (HEC-1-AR, HEC-1-BR, HEC-151R, Nou-1R) and observed that acquired resistance was consistently accompanied by marked upregulation of FGFR1-3 (**Figure R14b**; see also **Fig. 4c**), together with

right-shifted BYL719 dose-response curves and increased IC_{50} values (**Figure R14c**; see also **Fig. 4b**).

To directly test causality, we used both RNAi-mediated knockdown and enforced overexpression approaches. shRNA-mediated knockdown of FGFR1-3 (shR1R2R3) in BYL719-resistant HEC-1-AR and SNG-M lines significantly resensitized these cells to BYL719, as evidenced by left-shifted dose-response curves and reduced IC_{50} values (**Figure R14d**; see also **Supplementary Fig. 4d**). Conversely, enforced overexpression of FGFR1-3 in APM tumor cells (APM/FGFR1/2/3) conferred resistance to BYL719 both in vitro and in vivo, resulting in increased IC_{50} values in culture and markedly attenuated BYL719-mediated tumor growth inhibition in allografts compared with vector controls (**Figure R14e, f**; see also **Fig. 4d**).

Together, these new data provide direct causal confirmation that FGFR overexpression is sufficient to drive PI3Kα inhibitor resistance, and that genetic suppression of FGFR1-3 can reverse this phenotype, thereby strengthening the mechanistic link between FGFR signaling and PI3Kα inhibitor resistance in endometrial cancer models.

Moreover, besides phosphorylation of FRS2 and ERK, the authors should also evaluate the FGFRi impact on PI3K signaling (e.g., AKT, S6, etc.) as well as STAT3. Those signaling axes are known to mediated oncogenic FGFR pathway transduction besides MAPK. Speaking of, in Fig. 2b, do the p-AKT levels in resistant tumors represent reactivation of PI3K signaling due to FGFR? The levels seem definitely higher than the ones observed in Fig. 1g upon PI3K blockade (but before occurrence of resistance).

We appreciate this important mechanistic comment. In response, we systematically examined the impact of FGFR blockade on multiple downstream signaling pathways, including MAPK-ERK, PI3K-AKT, and STAT3, across several FGFR-high, PI3Kα inhibitor-resistant models.

(1) In the APM/Rec1 system, we compared primary APM tumors, FGFR-high recurrent Rec1 tumors, and Rec1 tumors with shRNA-mediated FGFR1-3 knockdown. Relative to APM tumors, Rec1 tumors show marked upregulation of FGFR1-3 accompanied by increased p-ERK1/2 and activation of mTOR outputs (p-S6RP and p-4EBP1), whereas p-STAT3 levels remain largely unchanged (**Figure R15a**; see also **Supplementary Fig. 4e**). Genetic suppression of FGFR1-3 in Rec1 tumors substantially reduces p-ERK1/2 and p-S6RP/p-4EBP1(**Figure R15a**; see also **Supplementary Fig. 4e**), indicating that FGFR upregulation primarily sustains MAPK-mTOR signaling in the recurrent setting. Notably, p-AKT is robust in APM tumors but not maintained in Rec1 tumors, consistent with doxycycline withdrawal and inactivation of PIK3CA-H1047R. Thus, AKT is not the dominant effector of FGFR-driven signaling in this model (**Figure R15a**; see also **Supplementary Fig. 4e**).

    Regarding your question comparing p-AKT levels in Fig. 2b versus Fig. 1g, the apparent differences reflects distinct experimental contexts and exposure conditions. Following doxycycline withdrawal, most recurrent tumors show rapid loss of PIK3CA-H1047R expression and correspondingly low p-AKT; only one recurrent lesion (Rec5) retains relatively higher p-AKT, consistent with partial maintenance of PIK3CA activity.

(2) We then analyzed human EC cell lines. In matched PI3Kα inhibitor-sensitive and -resistant pairs

(HEC-1-A vs HEC-1-AR and HEC-1-B vs HEC-1-BR), BYL719 effectively suppresses p-AKT and p-PRAS40 in both parental and resistant cells. However, phosphorylation of downstream mTOR effectors (p-P70S6K, p-S6RP, p-4EBP1, and p-eIF4B) is markedly reduced only in the sensitive lines and largely preserved in the resistant derivatives (**Figure R15b**; see also **Fig. 4e**), indicating uncoupling of mTOR output from AKT in the resistant state. In contrast, p-ERK1/2 is strongly elevated in resistant cells and shows minimal response to BYL719, consistent with MAPK pathway activation as a key adaptive resistance mechanism.

(3) To directly test the role of FGFR signaling, we treated HEC-1-AR cells with FGFR inhibitors alone or in combination with BYL719. Both lenvatinib and pemigatinib, particularly in combination with BYL719, markedly suppress p-FRS2, p-ERK1/2, and downstream mTOR targets (p-S6RP, p-4EBP1, p-eIF4B), whereas p-STAT3 remains largely unchanged (**Figure R15c**; see also **Fig. 4f**). These data indicate that FGFR blockade primarily attenuates the FRS2-MAPK-mTOR axis rather than AKT or STAT3 signaling in resistant human models (**Figure R15d**; see also **Supplementary Fig. 4f**)..

(4) Finally, using pathway-selective inhibitors, we systematically interrogated MAPK, PI3K-AKT, and STAT3 signaling to identify downstream mediators of FGFR-driven immune suppression (**Figure R10**). Pharmacologic MAPK inhibition most closely recapitulated the immunomodulatory effects of FGFR blockade, including increased tumor cell MHC-I expression and reduced M2-type TAMs in vivo. In contrast, inhibition of PI3K or STAT3 did not produce comparable effects on antigen presentation or myeloid composition.

Collectively, these data establish FGFR activation as a key driver of PI3Kα inhibitor resistance and clarify that both resistance and the associated immune phenotype are mediated predominantly through the MAPK-ERK-S6 axis, with minimal contribution from AKT or STAT3 signaling in this context (**Figure R15d**; see also **Supplementary Fig.4f**).

5. Fig. 3c: The authors should confirm the pre-existence of FGFR1/2 high and the absence of FGFR3 high epithelial cells in the primary tumors by using orthogonal, protein-level methods (for instance, IHC or FACS). This should be compared to resistant tumors.

We thank you for this insightful suggestion. To validate our snRNA-seq findings at the protein level using orthogonal approaches, we performed both immunohistochemistry (IHC) and flow cytometry (FACS) analyses on matched primary APM and recurrent Rec1 tumors.

IHC quantification shows that primary APM tumors contain $FGFR1^{high}$ and $FGFR2^{high}$ epithelial cell populations but essentially no detectable $FGFR3^{high}$ cells, whereas recurrent Rec1 tumors display strong and diffuse staining for FGFR1, FGFR2, and FGFR3 (**Figure R16a**; see also **Supplementary Fig. 2e**). Consistently, FACS analysis of dissociated tumor cells demonstrates a rightward shift in surface expression of FGFR1, FGFR2, and particularly FGFR3 in Rec1 tumors compared with APM tumors (**Figure R16b**; see also **Supplementary Fig. 2f**).

These protein-level data are fully concordant with our snRNA-seq results and support the conclusion that $FGFR1/2^{high}$ populations are already present in primary tumors, whereas FGFR3 upregulation is specifically associated with tumor recurrence. These findings and the corresponding panels have been incorporated into the revised manuscript (**Supplementary Fig. 2e, f**).

6. Fig. 4b/d: The authors should perform full dose-titration curves rather than just showing few titration points, especially for the resistant models.

Thank you for this valuable suggestion. In response, we performed full dose-response assays for both parental and resistant human EC lines, as well as in the corresponding mouse EC models. The revised manuscript now includes these expanded titration curves in **Fig. 4b and d** (see also **Figure R14c and e**). These data provide a more comprehensive and quantitative assessment of drug sensitivity and further confirm the differential responses between PI3Kα inhibitor- sensitive and -resistant models.

7. Fig. 4e/f: The authors should also evaluate phosphorylation of ERK (done in f), STAT3, 4EBP1, and EIF4B.

We thank you for this helpful suggestion. In the revised manuscript, we have evaluated the phosphorylation status of ERK, STAT3, 4EBP1, and eIF4B, providing further clarity on downstream signaling changes associated with FGFR and PI3Kα inhibition. The updated data have been incorporated into the revised **Fig. 4e and f** (see also **Figure R15b, c**), thereby strengthening the mechanistic conclusions of the study.

8. Fig. 4f-g: Also here, the manuscript would benefit from the use of an FGFR-specific inhibitor, such as pemigatinib.

We appreciate the suggestion to incorporate a more FGFR-specific inhibitor. In response, we performed additional experiments using pemigatinib, a highly selective FGFR1-3 inhibitor, in parallel with lenvatinib. As shown in the newly generated data (**Figure R7a, b**; incorporated into the revised manuscript as **Fig. 4f, g**), pemigatinib phenocopied the key effects of lenvatinib in FGFR-high, *PIK3CA*-mutant EC models. Specifically, pemigatinib suppressed FGFR downstream signaling and, when combined with BYL719 or GDC-0077, restored effective PI3Kα pathway inhibition and significantly enhanced growth suppression. These results demonstrate that the observed effects are attributable to on-target FGFR blockade rather than off-target activities of lenvatinib.

9. Fig. 4g: Rather than using single doses, the authors may consider using full dose-titration matrixes to support these data. This should also be put into direct comparison with parental HEC-1-A. Is an additive (or synergistic) effect with PI3Ki + FGFRi also observed in PI3Ki-sensitive, parental cell lines?

We appreciate this suggestion and agree that matrix-based analyses provide a more rigorous assessment of drug interactions. In response, we generated two-dimensional dose-titration matrices for PI3Kα and FGFR inhibition and directly compared parental HEC-1-A cells with their PI3Kα-resistant counterpart HEC-1-AR (**Figure R17**).

Using a BYL719-lenvatinib dose matrix analyzed with Combenefit (Loewe model), we observed largely additive to mildly synergistic effects in parental HEC-1-A cells, whereas HEC-1-AR cells showed a broader and more pronounced synergy landscape at clinically relevant concentrations (**Figure R17a, b**; updated **Fig. 4g** and **Supplementary Fig. 4l**). Consistent with these in vitro findings, combination treatment produced greater tumor growth inhibition than either single agent in both HEC-1-A and HEC-1-AR xenografts, with a particularly marked benefit in the resistant model (**Figure**

R17c,d; see also **Fig. 4h** and **Supplementary Fig. 4m**).

In addition, we performed analogous combination studies using the selective FGFR inhibitor pemigatinib. BYL719 (alpelisib) + pemigatinib and GDC-0077 (inavolisib) + pemigatinib displayed additive to synergistic effects in FGFR-high, PI3Kα-resistant HEC-1-AR cells by Combenefit analysis (**Figure R7b**; see also **Fig. 4g**), mirroring the results obtained with lenvatinib.

Together, these matrix-based analyses demonstrate that PI3Kα and FGFR co-inhibition is at least additive and often synergistic in both PI3Kα-sensitive and -resistant settings, with synergy most pronounced in the resistant, FGFR-high context.

10. Fig. 6d/e: Lenvatinib alone appears to have profound impact on the immune compartment. Can the authors rule out that lenvatinib directly impacts immune cells? The in vitro experiments (Fig. 5i/j) suggest that FGFRi indeed releases antigen presentation in a tumor cell-intrinsic manner. However, the tumor-intrinsic vs immune compartment impact of lenvatinib in vivo is less clear. The authors could deplete FGFRs in the tumor cells only and demonstrate that the impact on MHC-I presentation machinery and immune cells is akin to using systemic FGFR inhibitors. This would make a more compelling rationale for combining FGFRi with anti-PD1.

We appreciate this thoughtful comment and agree that distinguishing tumor-intrinsic versus systemic immune effects of lenvatinib is important for interpreting our data and for establishing a mechanistic rationale for combining FGFR inhibition with anti-PD-1 therapy.

To address this, we performed two complementary sets of experiments (**Figure R18**). First, we generated Rec1 allografts with stable, tumor-cell-intrinsic knockdown of FGFR1/2/3 (shFGFR1/2/3) and compared them with vector-control Rec1 tumors, in the absence of any systemic FGFR inhibitor treatment (**Figure R18a**). Tumor-intrinsic FGFR depletion was sufficient to recapitulate the key immune phenotypes observed with systemic lenvatinib treatment: shFGFR1/2/3 Rec1 tumors exhibited increased intratumoral $CD3^+$ and $CD8^+$ T cells, reduced M2-type TAMs, and elevated MHC-I expression on tumor cells (**Figure R18b**; see also **Fig. 5f**). Consistently, RT-qPCR analysis demonstrated coordinated upregulation of antigen processing and presentation genes (including *B2m, H2-d1, H2-k1, Tap1/2, Tapbp,* and *Erap1*) in shFGFR1/2/3 tumors relative to vector controls (**Figure R18c**; see also **Fig. 5f**). These findings indicate that FGFR signaling within tumor cells is sufficient to suppress antigen presentation and promote an immunosuppressive myeloid/T-cell landscape, and that the immunomodulatory effects of FGFR blockade are primarily tumor-intrinsic.

Second, to assess whether lenvatinib directly perturbs immune cells in vivo, we treated naïve, tumor-free mice with vehicle or lenvatinib for 12 days and analyzed spleen and peripheral blood immune populations by flow cytometry (**Figure R18d**). We did not observe significant differences in the frequencies of M2-like macrophages or T cells between vehicle- and lenvatinib-treated mice (**Figure R18e**; see also **Supplementary Fig. 5e**), arguing against a major direct effect of lenvatinib on systemic immune cell populations in the absence of tumor.

Together with our in vitro data showing that FGFR inhibition enhances antigen presentation in tumor cell-intrinsic manner, these results support a model in which the dominant immune effects of lenvatinib in our system are mediated through tumor-intrinsic FGFR blockade rather than direct suppression of immune cells. This, in turn, provides a strong mechanistic rationale for for combining FGFR inhibition

with anti-PD-1 therapy to enhance antitumor immunity and therapeutic efficacy.

11. In Fig. 2c, the authors describe a subset of resistant tumors that gain expression of cytokines but are devoid of FGFR gains. Do those resistant tumors also benefit from anti-PD1? If not addressed functionally, this should at least be discussed in the results and/or discussion of the manuscript.

We appreciate this insightful comment. The cytokine-high, FGFR-low recurrent tumors represent a biologically distinct subgroup that lacks FGFR amplification and thus is not the primary focus of the mechanistic investigations presented here.

Cytokine-driven microenvironments can be associated with both inflammatory signaling and immune-suppressive features, as well as replication stress-related vulnerabilities. Accordingly, responsiveness to anti-PD-1 therapy in this subgroup may be variable and potentially limited.

We have now incorporated a discussion in the revised manuscript emphasizing that distinct recurrent states (FGFR-high versus cytokine-high/FGFR-low) may exhibit differential therapeutic vulnerabilities and may require tailored combination strategies (Page 13, Lines 482-495).

We agree that direct functional evaluation of immune checkpoint blockade in cytokine-high recurrent tumors will be of considerable interest and remains an important direction for future investigation.

REDACTED: The previously included cytokine-high recurrent tumor-related data have been removed from the response letter, as these data were not incorporated into the revised manuscript and extend beyond the central focus of the present study.

12. In general, the manuscript would benefit from expanding the used PI3Ki to the newest generation of PI3Ka inhibitors, such as inavolisib.

We agree that it is important to demonstrate that our findings extend to newer-generation PI3Kα inhibitors. In response, we incorporated the next-generation PI3Kα inhibitor inavolisib (GDC-0077) into both correlative and functional analyses.

First, using a panel of human EC cell lines, we profiled sensitivity to inavolisib alongside BYL719 and correlated IC_{50} values with FGFR1-3 expression (**Figure R3a-c**; see also **Supplementary Fig. 4a**). Similar to BYL719, FGFR-high lines were relatively less sensitive to inavolisib, indicating that FGFR status predicts response to both first- and next-generation PI3Kα inhibitors.

Second, we evaluated inavolisib in vivo in the APM model. GDC-0077 monotherapy significantly suppressed APM tumor growth to a degree comparable to BYL719 (**Figure R20a, b**; see also **Supplementary Fig. 1i**), confirming its activity in this setting. We then assessed combined FGFR and PI3Kα inhibition in PI3Kα-resistant, FGFR-high human EC models. Combenefit analysis of two-dimensional dose matrices revealed additive to synergistic interactions for lenvatinib combined with either BYL719 or inavolisib in HEC-1-AR and SNG-M cells (**Figure R20c**; see also **Fig. 4g** and **Supplementary Fig. 4c**), demonstrating that PI3Kα/FGFR co-inhibition remains effective when a next-generation PI3Kα inhibitor is used.

Finally, we generated an inavolisib-resistant derivative (HEC-1-A iR) through stepwise does escalation. HEC-1-A iR cells displayed a pronounced rightward shift in inavolisib dose-response curves together with robust upregulation of FGFR1-3 (**Figure R20d**; see also **Supplementary Fig. 4b**), mirroring the FGFR-driven resistance mechanism observed with BYL719.

Together, these data demonstrate that FGFR-associated PI3Kα inhibitor resistance and its reversal by FGFR blockade are not limited to BYL719 but extend to the next-generation PI3Kα inhibitor inavolisib. These results have been incorporated and explicitly noted in the revised manuscript.

13. The authors should discuss in more detail and put their work into context of the current preclinical and clinical evidence for combining PI3Ki with FGFRi as well as FGFRi with ICB, whether actual cooperation of the combos has been observed, as well as the potential combo limitations and toxicities.

We thank you for this insightful and important suggestion. In response, we have substantially expanded the **Discussion** to place our findings within the broader context of current preclinical and clinical evidence supporting (i) combined PI3K-FGFR inhibition and (ii) FGFR inhibition in combination with immune checkpoint blockade.

For **PI3K-FGFR combinations**, we now discuss more detail how FGFR1 and FGFR2 drive intrinsic resistance, whereas FGFR3 is preferentially enriched in acquired resistance in our models. This distinction is consistent with reports across multiple tumor types in which FGFR activation contributes

to therapy resistance[16, 17, 18, 19]. We further incorporated preclinical studies demonstrating strong cooperation between PI3K and FGFR inhibition, including lung cancer models in which FGFR1-mediated resistance to EGFR inhibitors was overcome by combined FGFR-PI3K/Akt blockade, resulting in markedly superior antitumor activity compared with monotherapy[20]. In addition, we reference a Phase I trial evaluating alpelisib in combination with the FGFR inhibitor infigratinib, which demonstrated feasibility of co-administration at clinically relevant doses while also highlighting overlapping toxicities, such as hyperglycemia and hyperphosphatemia, underscoring the need for biomarker-guided patient selection and careful dose optimization[21]. Together, these additions strengthen the mechanistic and translational rationale for PI3Kα-FGFR dual inhibition observed in our SEC models.

For **FGFRi-ICB combinations**, we expanded the discussion of FGFR-mediated immune evasion and incorporated preclinical evidence from multipe cancer types demonstrating that FGFR inhibition can enhance anti-PD-1 activity by increases $CD8^{+}$ T-cell infiltration, reducing regulatory T cells, normalizing tumor vasculature, downregulating PD-L1 expresssion, and converting immunologically “cold” tumors into “hot” ones[22, 23, 24, 25]. We also highlight relevant clinical data, including the robust efficacy of lenvatinib plus pembrolizumab in advanced EC and emerging early-phase trials evaluating FGFRi-ICB combinations, to emphasize the translational relevance of these mechanisms[26, 27].

Finally, the revised Discussion explicitly addresses potential **limitations and toxicities** of these combination strategies. While our preclinical studies demonstrate good tolerability in mice, clinical experience with PI3Ki, FGFRi, and ICB indicates distinct and sometimes overlapping adverse-event profiles. These considerations will necessitate thoughtful optimization of dosing, scheduling, and patient selection. We concluded by emphasizing the dual role of FGFR signaling in driving both therapy resistance and immune evasion, the cooperative efficacy observed when FGFR inhibitors are combined with PI3Ki or ICB, and the practical clinical considerations that will be critical for translating PI3K-FGFR-ICB combination strategies into the clinic.

References:

1. Xue Y, *et al.* Preclinical research models for endometrial cancer: development and selection of animal models. *Front Oncol* **15**, 1512616 (2025).

2. Vidal-Sabanes M, *et al.* Endometrial cancer progression driven by PTEN-deficiency requires miR-424(322)(~)503. *Cell Death Dis* **16**, 705 (2025).

3. Mirantes C, *et al.* An inducible knockout mouse to model the cell-autonomous role of PTEN in initiating endometrial, prostate and thyroid neoplasias. *Dis Model Mech* **6**, 710–720 (2013).

4. Cassier PA, *et al.* Netrin-1 blockade inhibits tumour growth and EMT features in endometrial cancer. *Nature* **620**, 409–416 (2023).

5. Wu L, *et al.* Discovery of Pemigatinib: A Potent and Selective Fibroblast Growth Factor Receptor (FGFR) Inhibitor. *J Med Chem* **64**, 10666–10679 (2021).

6. Bergholz JS, Wang Q, Kabraji S, Zhao JJ. Integrating Immunotherapy and Targeted Therapy in Cancer Treatment: Mechanistic Insights and Clinical Implications. *Clin Cancer Res* **26**, 5557–5566 (2020).

7. Fu R, Jiang S, Li J, Chen H, Zhang X. Activation of the HGF/c-MET axis promotes lenvatinib resistance in hepatocellular carcinoma cells with high c-MET expression. *Med Oncol* **37**, 24 (2020).

8. Lee YS, *et al.* Anti-cancer Effects of HNHA and Lenvatinib by the Suppression of EMT-Mediated Drug Resistance in Cancer Stem Cells. *Neoplasia* **20**, 197–206 (2018).

9. Yang L, *et al.* M2 macrophage inhibits the antitumor effects of Lenvatinib on intrahepatic cholangiocarcinoma. *Front Immunol* **14**, 1251648 (2023).

10. Juric D, *et al.* Convergent loss of PTEN leads to clinical resistance to a PI(3)Kalpha inhibitor. *Nature* **518**, 240–244 (2015).

11. Costa C, *et al.* Measurement of PIP3 levels reveals an unexpected role for p110beta in early adaptive responses to p110alpha-specific inhibitors in luminal breast cancer. *Cancer Cell* **27**, 97–108 (2015).

12. Elkabets M, *et al.* mTORC1 inhibition is required for sensitivity to PI3K p110alpha inhibitors in PIK3CA-mutant breast cancer. *Sci Transl Med* **5**, 196ra199 (2013).

13. Elkabets M, *et al.* AXL mediates resistance to PI3Kalpha inhibition by activating the EGFR/PKC/mTOR axis in head and neck and esophageal squamous cell carcinomas. *Cancer Cell* **27**, 533–546 (2015).

14. Le X, *et al.* Systematic Functional Characterization of Resistance to PI3K Inhibition in Breast Cancer. *Cancer Discov* **6**, 1134–1147 (2016).

15. Hopkins BD, *et al.* Suppression of insulin feedback enhances the efficacy of PI3K inhibitors. *Nature* **560**, 499–503 (2018).

16. Gan Y, Wientjes MG, Au JL. Expression of basic fibroblast growth factor correlates with resistance to paclitaxel in human patient tumors. *Pharm Res* **23**, 1324–1331 (2006).

17. Zhou Y, Wu C, Lu G, Hu Z, Chen Q, Du X. FGF/FGFR signaling pathway involved resistance in various cancer types. *J Cancer* **11**, 2000–2007 (2020).

18. Formisano L, *et al.* Aberrant FGFR signaling mediates resistance to CDK4/6 inhibitors in ER+ breast cancer. *Nat Commun* **10**, 1373 (2019).

19. Turner N, *et al.* FGFR1 amplification drives endocrine therapy resistance and is a therapeutic target in breast cancer. *Cancer Res* **70**, 2085–2094 (2010).

20. Terp MG, *et al.* Combined FGFR and Akt pathway inhibition abrogates growth of FGFR1 overexpressing EGFR-TKI-resistant NSCLC cells. *NPJ Precis Oncol* **5**, 65 (2021).

21. Hyman DM, *et al.* Combined PIK3CA and FGFR Inhibition With Alpelisib and Infigratinib in Patients With PIK3CA-Mutant Solid Tumors, With or Without FGFR Alterations. *JCO Precis Oncol* **3**, 1–13 (2019).

22. Okato A, *et al.* FGFR inhibition augments anti-PD-1 efficacy in murine FGFR3-mutant bladder cancer by abrogating immunosuppression. *J Clin Invest* **134**, (2024).

23. Yi C, *et al.* Lenvatinib Targets FGF Receptor 4 to Enhance Antitumor Immune Response of Anti-Programmed Cell Death-1 in HCC. *Hepatology* **74**, 2544–2560 (2021).

24. Palakurthi S, *et al.* The Combined Effect of FGFR Inhibition and PD-1 Blockade Promotes Tumor-Intrinsic Induction of Antitumor Immunity. *Cancer Immunol Res* **7**, 1457–1471 (2019).

25. Xiang H, *et al.* Preclinical characterization of bemarituzumab, an anti-FGFR2b antibody for the treatment of cancer. *MAbs* **13**, 1981202 (2021).

26. Makker V, *et al.* Lenvatinib plus Pembrolizumab for Advanced Endometrial Cancer. *N Engl J Med* **386**, 437–448 (2022).

27. Chan SL, *et al.* A first-in-human phase 1/2 study of FGF401 and combination of FGF401 with spartalizumab in patients with hepatocellular carcinoma or biomarker-selected solid tumors. *J Exp Clin Cancer Res* **41**, 189 (2022).

a

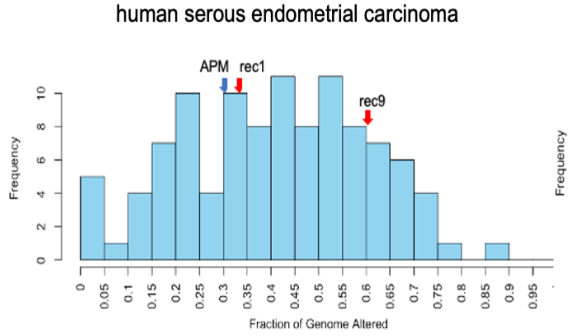

b

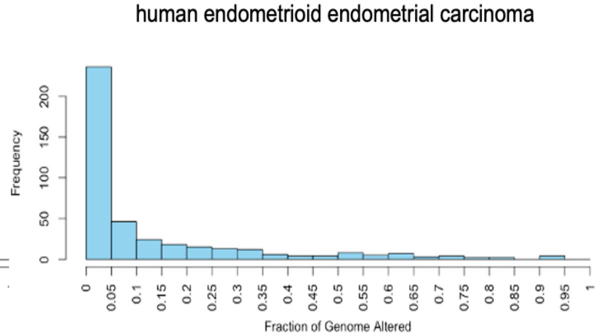

c

		APM		Rec1		Rec9	
Method	Total numbers of bases	Number of CNV	FGA	Number of CNV	FGA	Number of CNV	FGA
CNVkit (exome)	912677426	299641482	0.33	268929392	0.3	559340263	0.61

Figure R1. APM tumors and their recurrent derivatives exhibit SEC-like high fractions of genome altered.

(a) Distribution of fraction of genome altered (FGA) in TCGA serous endometrial carcinomas (SEC), demonstrating a high burden of copy-number alterations (median FGA = 0.41).

(b) Distribution of FGA in non-serous TCGA endometrial cancer samples, which display a markedly lower copy-number alteration burden (median FGA ≈ 0.07).

(c) FGA values for the APM primary tumors model and their recurrent tumors (Rec1 and Rec9), showing FGA values of 0.30 and 0.61, respectively, comparable to those observed in human SEC.

REDACTED: additional experimental model data not included in revised manuscript.

a A panel of human EC lines with known mutations in major oncogenes and tumor suppressors

	HHUA	EN	SNG-M	MFE-296	HEC-6	HEC-1-A	ARK-1	HEC-151	HEC-1-B	Nou-1
**PIK3CA**	R88Q	T1025A	R88Q	P539R	R108H	G1049R	E542K	C420R	G1049R	R38H
**TP53**	A138V	R273H	R280T	Y220C	R273H	R248Q	R248W	V31I	R248Q	WT
**PTEN**	V290fs	K267fs	V290*	R130Q	V290*	WT	WT	Y76*	WT	no mRNA
**PIK3R1**	WT	X279-splice	WT	WT	WT	WT	WT	WT	WT	WT
**KRAS**	G12V	WT	G12V	WT	V160A	G12D	WT	WT	D12D	G12D
**EGFR**	WT	WT	WT	WT	A289V	WT	WT	WT	WT	WT
**HER2-amplified**	No	No	No	No	No	No	Yes	No	No	No
**Pathological subtypes**	Endometrial adenocarcinoma	Endometrial carcinoma	Endometrial adenocarcinoma	EEC	Uterine adenosquamous carcinoma	Endometrial carcinoma	SEC	Endometrial carcinoma	Endometrial carcinoma	Endometrial carcinoma
**BYL719 IC_{50} (μM)**	27	16.8	12.3	12.3	5.1	3.2	2.9	2.8	2.4	0.9
**Inavolisib IC_{50} (μM)**	15.2	12.9	20	6.7	3.7	2.0	0.05	0.6	0.6	0.6

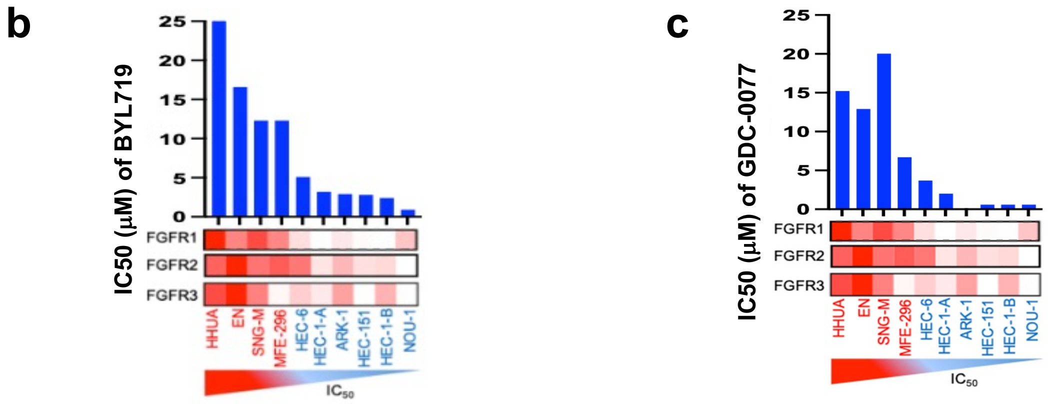

Figure R3. Histologic/molecular annotation of the endometrial cancer cell line panel and FGFR-dependent modulation of PI3Kα inhibitor response.

(a) Summary of the ten human endometrial cancer (EC) cell lines used in this study, showing histologic/molecular subtype (endometrioid vs non-endometrioid/serous-like), key mutations (including **PIK3CA**, **PTEN**, and **TP53**) based on published pathology reports and publicly available genomic datasets. ARK1, the only bona fide serous EC (SEC) line in the panel harboring a *PIK3CA* mutation, is indicated.

(b) Relationship between FGFR expression (as determined by qPCR) and sensitivity to the PI3Kα inhibitor BYL719 (alpelisib) across the EC cell line panel. IC_{50} values are plotted against FGFR expression, with histologic subtype annotated. Higher FGFR expression is associated with reduced sensitivity to BYL719, independent of subtype.

(c) Relationship between FGFR expression and sensitivity to the next-generation PI3Kα inhibitor GDC-0077 (inavolisib) in the same cell line panel. Consistent with (b), $FGFR^{high}$ lines show relative resistance, whereas $FGFR^{low}$ lines remain sensitive, supporting FGFR-driven modulation of PI3Kα inhibitor response as a largely subtype-agnostic resistance mechanism in EC.

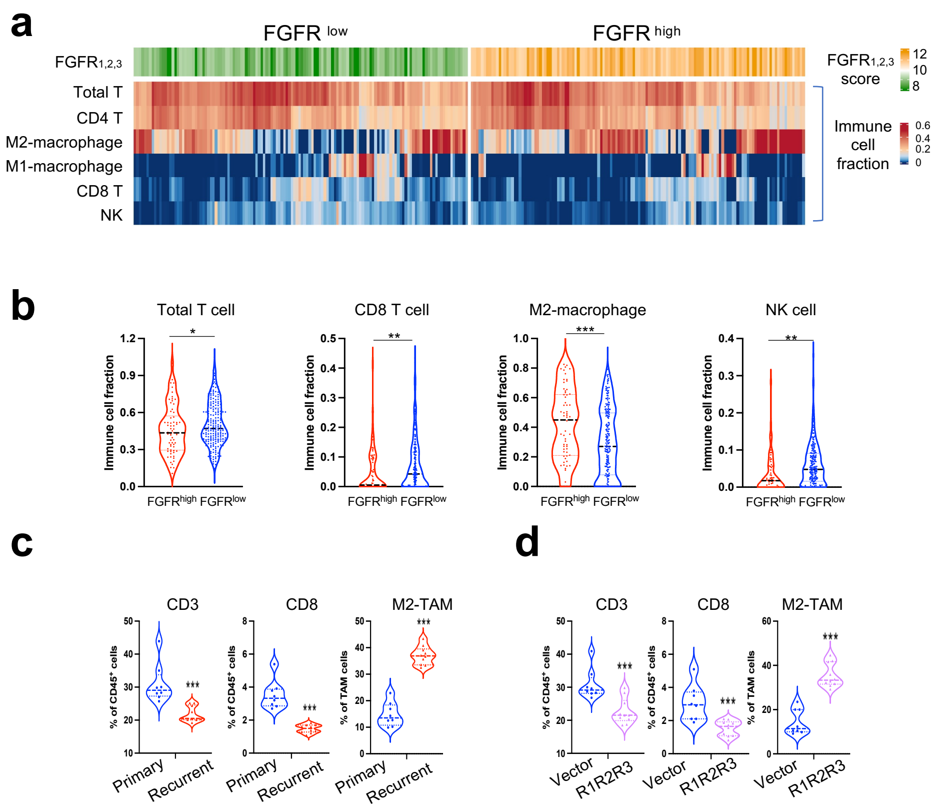

Figure R4. High FGFR signaling is associated with an immunosuppressive tumor microenvironment in TCGA EC and FGFR-high recurrent models.

(a) Heatmap showing relative FGFR1-3 expression and immune cell composition across uterine cancer samples from the TCGA UCEC cohort. A composite FGFR1-3 score was calculated for each sample as the mean log_2 expression of FGFR1, FGFR2, and FGFR3. Immune cell fractions were estimated using CIBERSORT and normalized to sum to 1 per sample. Samples were ordered by increasing FGFR composite score and stratified into FGFR-low (bottom 25%, n=66) and FGFR-high (top 25%, n=66) groups.

(b) Violin plots showing CIBERSORT-estimated immune cell fractions in FGFR-high tumors (top 25% by FGFR composite score, n=66) compared with all remaining tumors (FGFR-low/others, n=198). FGFR-high tumors exhibit reduced total T cells, $CD8^+$ T cells, and NK cells, along with an increased proportion of M2 macrophages, consistent with a more immunosuppressive and less inflamed tumor microenvironment. Statistical significance is indicated.

(c) Immune profiling of primary (APM) and recurrent (Rec1) tumors with spontaneous FGFR overexpression. Flow cytometry analysis of FGFR-high recurrent versus FGFR-low primary tumors (n = 8-10 per group) shows reduced T-cell infiltration ($CD3^+$, $CD8^+$) and an increased proportion of M2-like TAMs in recurrent tumors, mirroring the immune pattern observed in FGFR-high TCGA tumors.

(d) Immune profiling of primary APM tumors engineered to overexpress FGFRs. Flow cytometry analysis show decreased T-cell infiltration ($CD3^+$, $CD8^+$) and enrichment of M2/TAM-like myeloid cells in FGFR-overexpressing tumors relative to control tumors.

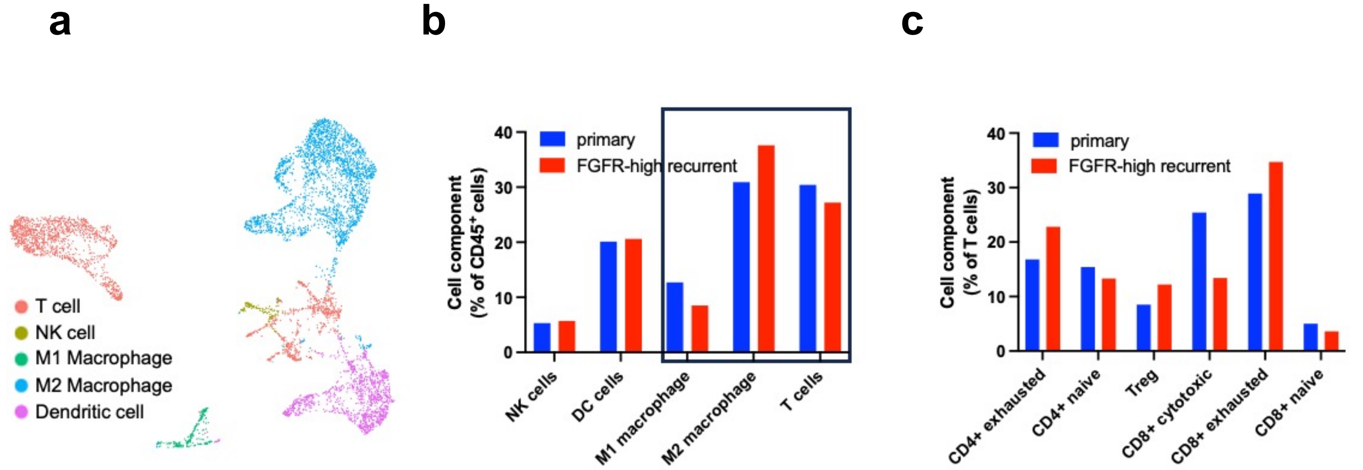

Figure R5. High-resolution single-cell immune profiling of primary APM vs FGFR-high recurrent Rec1 tumors.

(a) UMAP projection of $CD45^+$ immune cells from primary (APM) and recurrent (Rec1) tumors following reclustering and annotation into major immune populations, including T cells, NK cells, M1- and M2-like macrophages, and dendritic cell-enriched clusters.

(b) Quantification of immune cell subsets in primary versus FGFR-high recurrent tumors, showing as proportions of total $CD45^+$ cells per tumor. FGFR-high recurrent tumors exhibit an increased abundance of M2-like TAMs, together with a concomitant reduction in M1-like macrophages and overall T-cell infiltration.

(c) Composition of T-cell subsets in primary versus recurrent tumors. FGFR-high recurrent tumors display marked depletion of cytotoxic $CD8^+$ T cells and relative enrichment of exhausted T cells and Tregs, indicative of an immunosuppressive T-cell compartment.

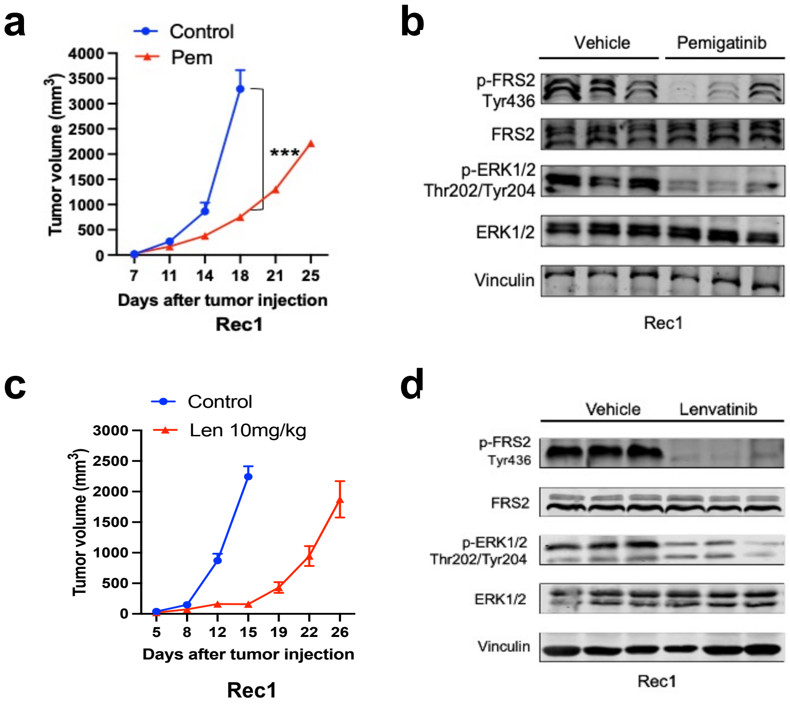

Figure R6. Effects of FGFR inhibitors pemigatinib and lenvatinib on FGFR-high recurrent tumors.

(a) Tumor growth curves of $FGFR_{1\text{-}3}$ overexpressing recurrent tumors (Rec1) treated with vehicle or the selective FGFR inhibitor pemigatinib (n = 8-10 per group; mean ± SEM). Pemigatinib significantly suppresses Rec1 tumor growth, indicating functional dependence on FGFR signaling.

(b) Western blot analysis of of FGFR pathway activation in Rec1 tumors after 12 days of treatment. Pemigatinib reduces phosphorylation of FRS2 and ERK, confirming effective on-target inhibition of FGFR signaling in vivo.

(c, d) For comparison, lenvatinib treatment in Rec1 tumors produces a similar pattern of growth inhibition (c) and suppression of p-FRS2 and p-ERK (d), closely mirroring the effects observed with pemigatinib (see also Fig. 2f, g).

Together, these data demonstrate that FGFR blockade per se is sufficient to impair the growth of FGFR-high recurrent tumors, supporting a critical role for FGFR signaling in maintaining the recurrent tumor state.

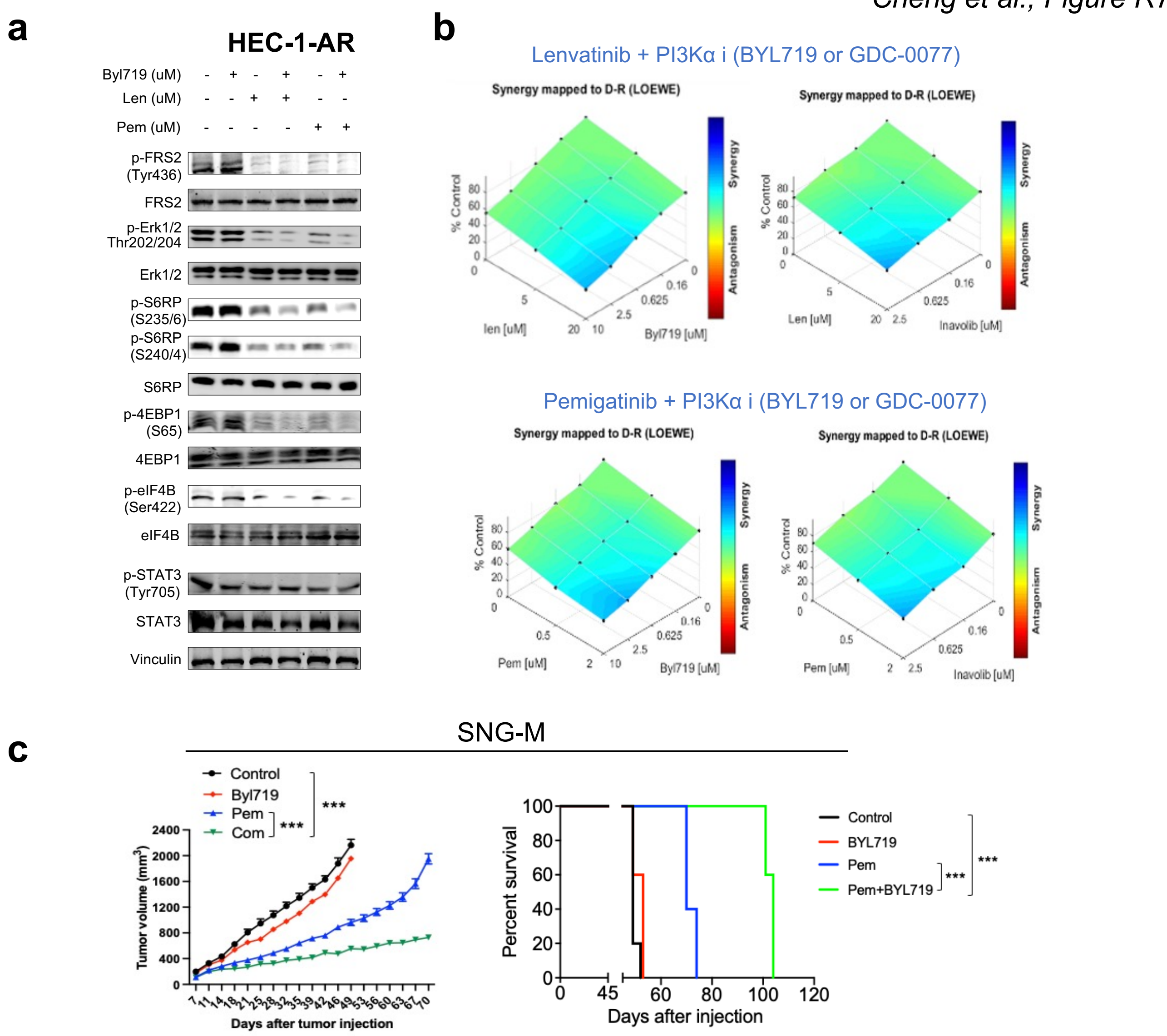

Figure R7. FGFR inhibition restores PI3Kα inhibitor sensitivity in FGFR-high, *PIK3CA*-mutant human endometrial cancer models.

(a) Western blot analysis of PI3K and FGFR pathways in HEC-1-AR cells treated with BYL719 in combination with either lenvatinib or pemigatinib (1 μM BYL719, 20 μM lenvatinib, 2 μM pemigatinib, or their combinations). Both pemigatinib and lenvatinib enhance PI3Kα inhibitor-mediated suppression of downstream signaling, indicating restoration of pathway blockade in FGFR-high cells.

(b) Effects of combined PI3Kα and FGFR inhibition on HEC-1-AR cell viability. Cells were treated with lenvatinib + BYL719 (or GDC-0077), pemigatinib + BYL719 (or GDC-0077) over a range of concentrations. Combination effects were quantified using the Combenefit synergy-antagonism model, which shows additive to synergistic interactions between PI3Kα inhibitors and FGFR inhibitors.

(c) In vivo efficacy of combined PI3Kα and FGFR inhibition in SNG-M xenografts. Tumor-bearing mice were treated with vehicle, BYL719 alone, pemigatinib alone, or the BYL719 plus pemigatinib combination. Whereas single-agent treatments produced limited or modest tumor control, combined pemigatinib and BYL719 treatment resulted in marked tumor growth inhibition, demonstrating that selective FGFR blockade is sufficient to resensitize FGFR-high tumors to PI3Kα inhibition (n=8-10 per group).

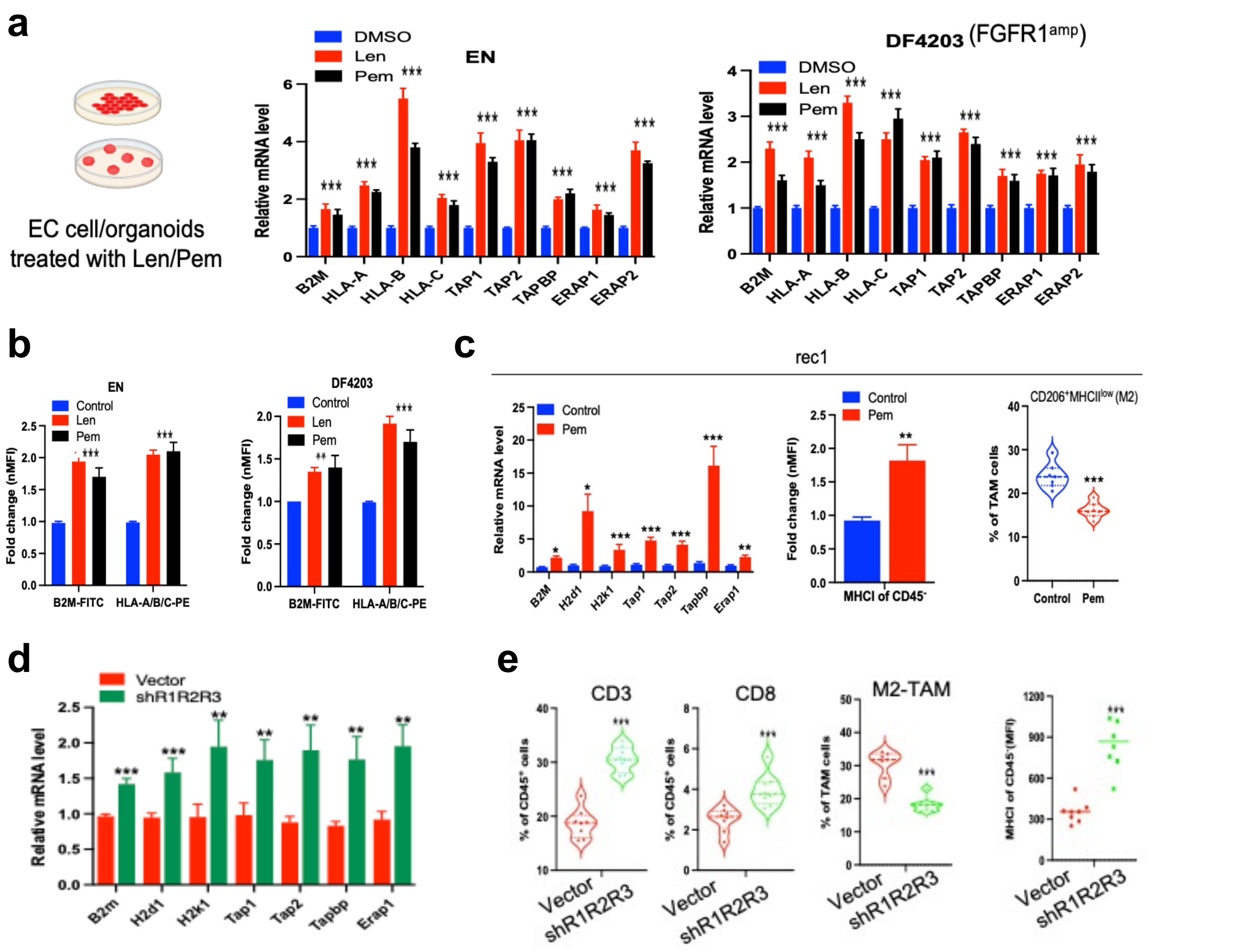

Figure R8. FGFR-specific regulation of antigen presentation and myeloid/T-cell composition in FGFR-high endometrial cancer models.

(a, b) Expression of genes involved in antigen processing and presentation in FGFR1-3-overexpressing EN cells and FRFG1- amplified PDO DF4203 treated with vehicle, lenvatinib, or pemigatinib. Both FGFR inhibitors induce comparable upregulation of antigen-presentation programs (see also Fig. 5j, k).

(c) Flow cytometry-based immune profiling of Rec1 tumors treated in vivo with vehicle, or pemigatinib, showing changes in tumor cell antigen processing and presentation, MHC-I expression, and macrophage subsets. Pemigatinib increases overall T-cell infiltration and reduced M2-type TAMs relative to vehicle (n = 8-10 per group).

(d) Genetic FGFR1-3 knockdown in Rec1 tumors similarly increased expression of antigen processing and presentation genes, mirroring the effects observed with lenvatinib and pemigatinib treatment, indicating that these changes reflect on-target FGFR blockade rather than off-target activities of lenvatinib.

(e) Quantification of total T cells and CD8$^+$ T cells, as well as M2-type TAMs, in FGFR1–3 knockdown Rec1 tumors reveals increased infiltration of total and CD8$^+$ T cells accompanied by a reduction in M2-type TAMs. These findings indicate that FGFR inhibition promotes enhanced antigen presentation, T-cell enrichment, and macrophage repolarization within the tumor microenvironment.

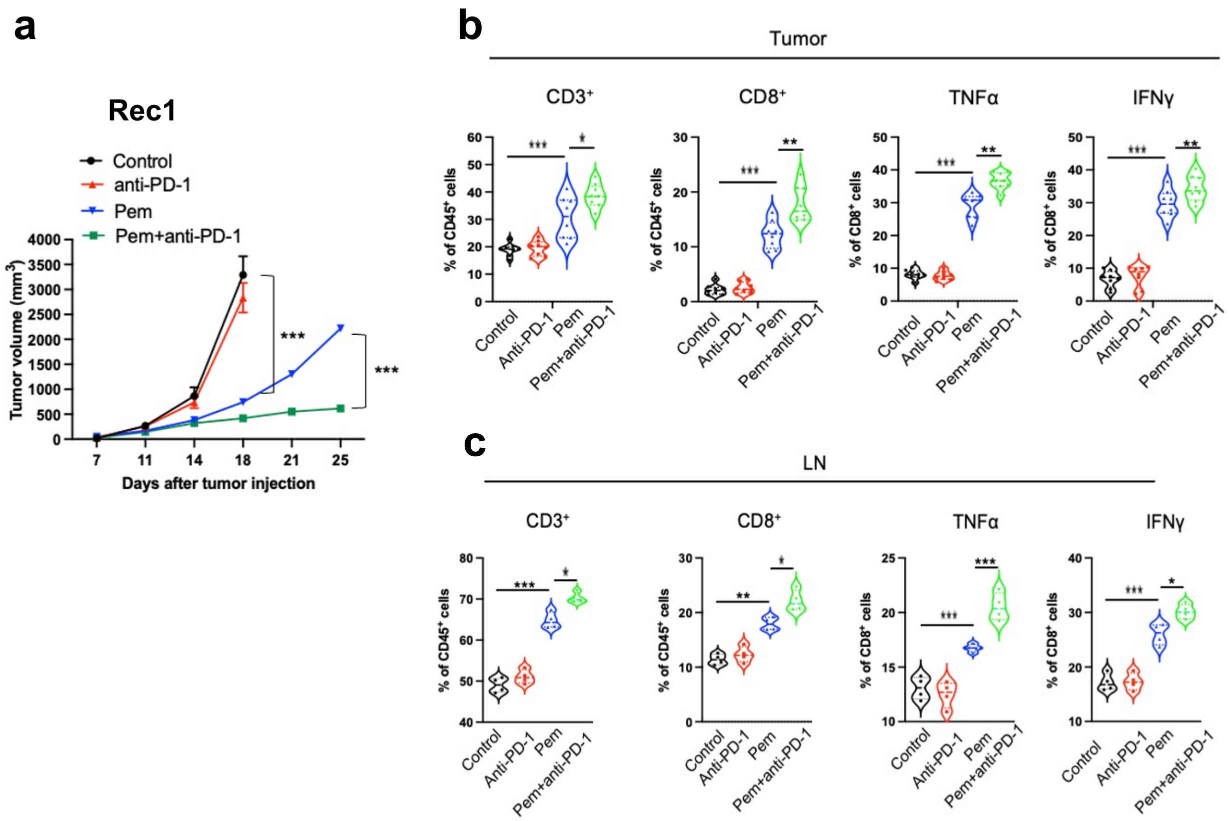

Figure R9. Pemigatinib enhances antitumor immunity and response to PD-1 blockade in Rec1 tumors.

(a) Tumor growth curves of Rec1-bearing mice treated with vehicle control, anti-PD-1, pemigatinib (Pem), or the combination pemigatinib and anti-PD-1 (Pem + anti-PD-1) (n = 8-10 per group). Combination therapy produces the most pronounced inhibition of tumor growth.

(b) Flow cytometric analysis of tumor-infiltrating lymphocytes from the indicated treatment groups. Shown are the frequencies of $CD3^+$ and $CD8^+$ T cells among $CD45^+$ cells, as well as TNFα- and IFNγ-producing $CD8^+$ T cells among total $CD8^+$ cells (n = 8-10 per group).

(c) Parallel flow cytometric analysis of draining lymph nodes (LN), showing $CD3^+$ and $CD8^+$ T cells among $CD45^+$ cells and TNFα- and IFNγ-producing $CD8^+$ T cells among total $CD8^+$ cells (n = 8-10 per group). Pemigatinib alone and in combination with anti-PD-1 enhances T-cell abundance and effector cytokine production in both tumors and lymph nodes.

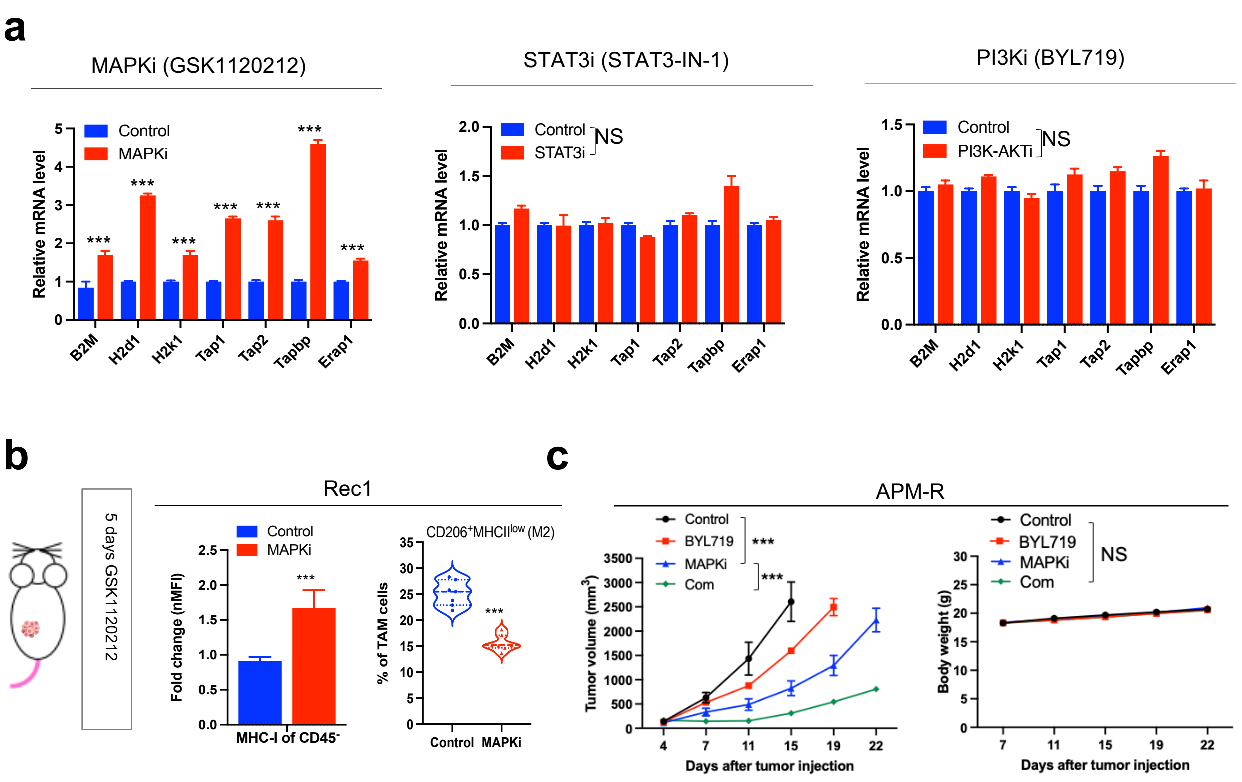

Figure R10. MAPK-ERK signaling is the dominant downstream effector of FGFR activation.

(a) RT-qPCR analysis of genes involved in antigen processing and presentation in Rec1 tumor cells treated in vitro with inhibitors targeting major FGFR downstream pathways: MAPK-ERK (MAPKi, GSK1120212), STAT3 (STAT3i, STAT3-IN-1), or PI3K-AKT (PI3Ki, BYL719). Inhibition of the MAPK pathway leads to robust upregulation of antigen presentation-related genes, whereas STAT3 or PI3K-AKT inhibition has minimal effect (NS, not significant).

(b) In vivo validation of MAPK-dependent immune modulation in Rec1 tumors. Flow cytometric analysis of tumors from mice treated with GSK1120212 for 5 days shows increased MHC-I expression (mean fluorescence intensity, MFI) on $CD45^-$ tumor cells (left) and a reduced percentage of $CD206^+MHCII^{low}$ M2-type TAMs (right), indicating enhanced antigen presentation and macrophage repolarization.

(c) Tumor growth curves and body weight trajectories of mice bearing FGFR-high, BYL719-resistant APM-R tumors treated with BYL719, GSK1120212, or the combination (n = 8 per group). Combination therapy produces the greatest inhibition of tumor growth without affecting body weight, supporting MAPK-ERK as a key functional downstream effector of FGFR activation in driving tumor progression and therapeutic resistance.

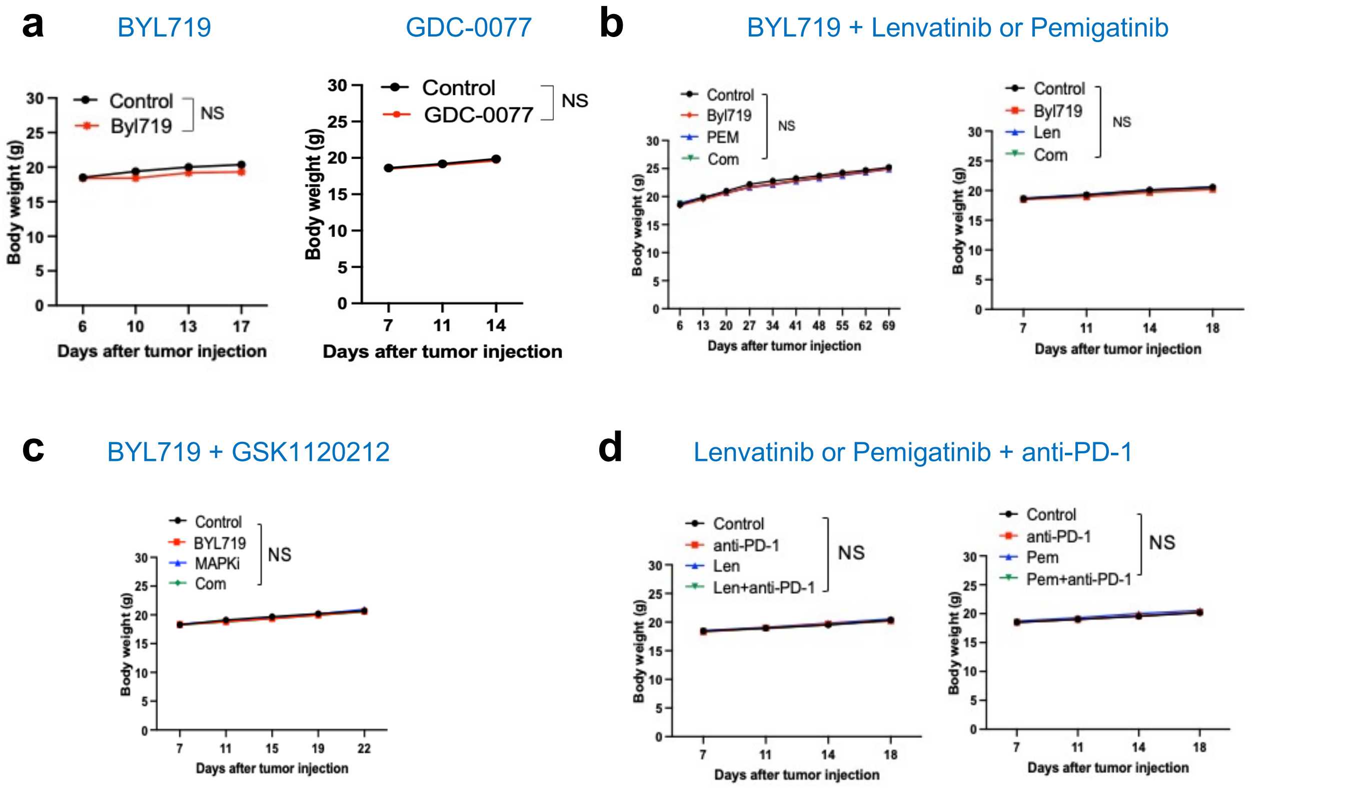

Figure R11. Longitudinal body weight trajectories of control and indicated treatment groups. No significant weight loss or evidence of treatment-associated toxicity was observed across any monotherapy or combination treatment regimen.

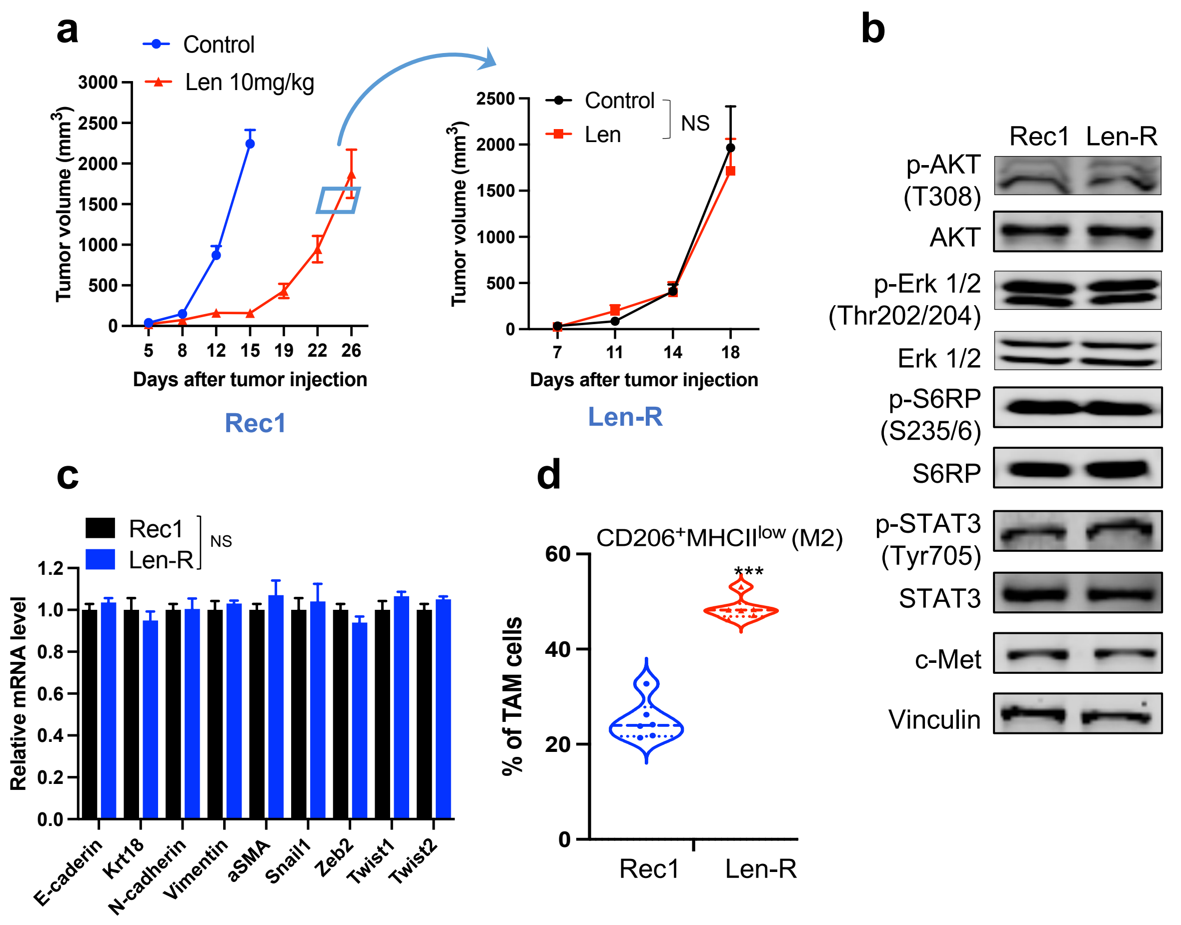

Figure R12. Adaptive resistance to lenvatinib in Rec1 tumors and associated microenvironmental remodeling.

(a) Generation of lenvatinib-resistant (Len-R) tumors. Rec1-bearing mice were treated continuously with lenvatinib until tumors became refractory (>20 days) , after which resistant tumors were harvested and transplanted into new recipients for two serial passages under continued lenvatinib treatment. Transplanted Len-R tumors exhibited minimal response to lenvatinib compared with parental Rec1 tumors, confirming the establishment of acquired resistance (n = 5 mice per group; mean ± SEM).

(b) Analysis of canonical signaling pathways in Len-R versus parental Rec1 tumors. Immunoblot analysis of STAT3, ERK, PI3K-AKT, and c-MET revealed no major differences in pathway activation between Len-R and parental Rec1 tumors, indicating that classical pathway reactivation is not the predominant mechanism of lenvatinib resistance.

(c) RT-PCR analysis of EMT-associated markers showed no significant differences between Rec1 and Len-R tumors.

(d) Flow cytometric quantification of TAM subsets revealed a significant increase in M2-type TAMs ($CD206^{+}MHCII^{low}$) in Len-R tumors compared with parental Rec1 tumors, consistent with microenvironmental remodeling toward an immunosuppressive state.

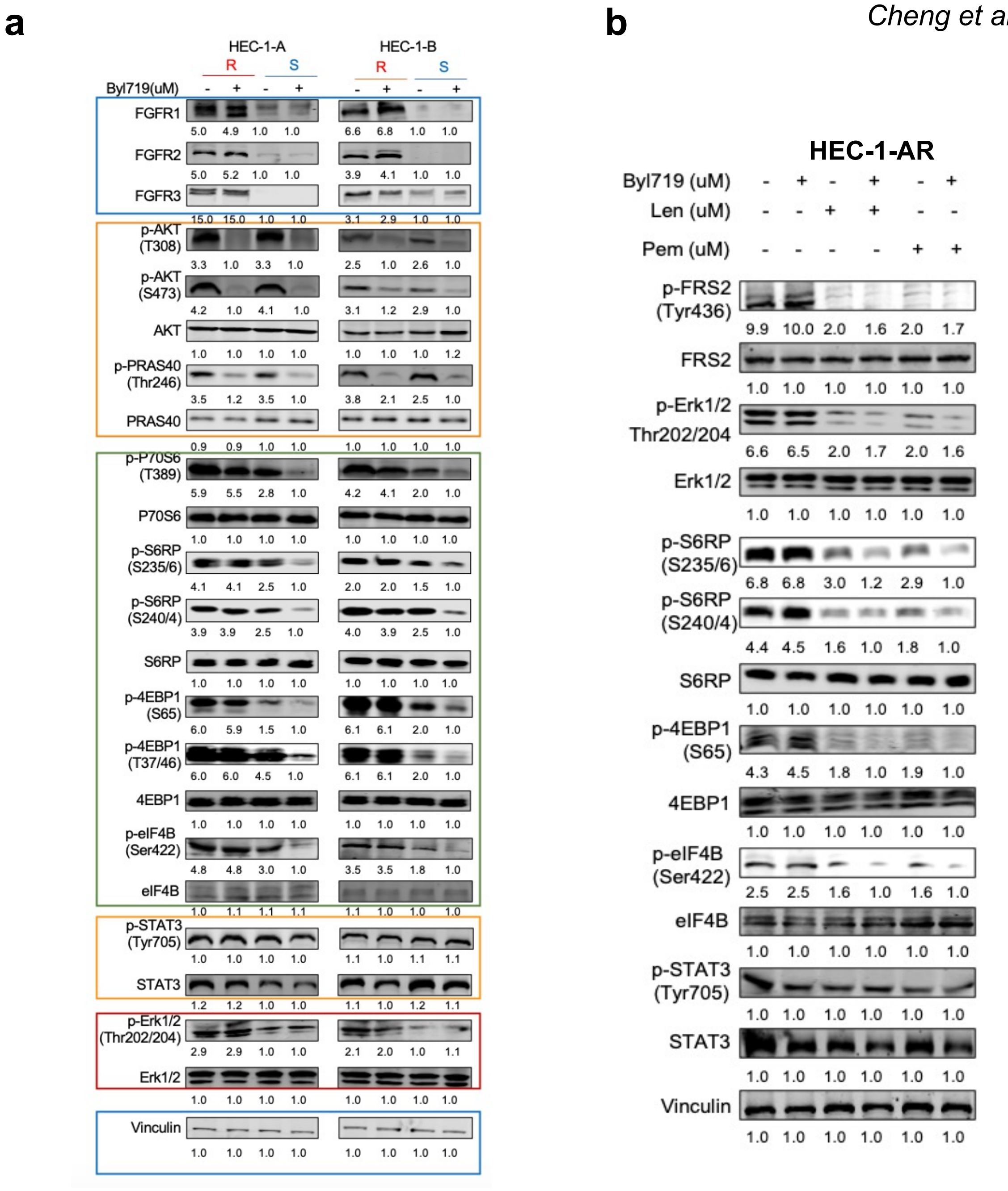

Figure R13. Quantification of FGFR-dependent signaling in BYL719-sensitive and -resistant cells.

(a) Western blot analysis of PI3K/AKT, MAPK, and STAT3 signaling in BYL719-sensitive (S) and -resistant (R) cells following 24-hour treatment with 1 μM BYL719. Representative blots from three independent biological replicates are shown under non-saturating exposure conditions, together with densitometric quantification of phospho-proteins normalized to their corresponding total proteins and loading controls.

(b) Western blot analysis of protein lysates from HEC-1-AR (BYL719-resistant) cells treated with 1 μM BYL719, 20 μM lenvatinib, 2 μM pemigatinib or the indicated combinations. Representative blots and corresponding densitometric quantification demonstrate that FGFR inhibition restores effective suppression of downstream MAPK signaling in resistant cells.

Quantification data represent the mean of three independent experiments. All uncropped blots and raw quantification data have been provided in the Source Data file.

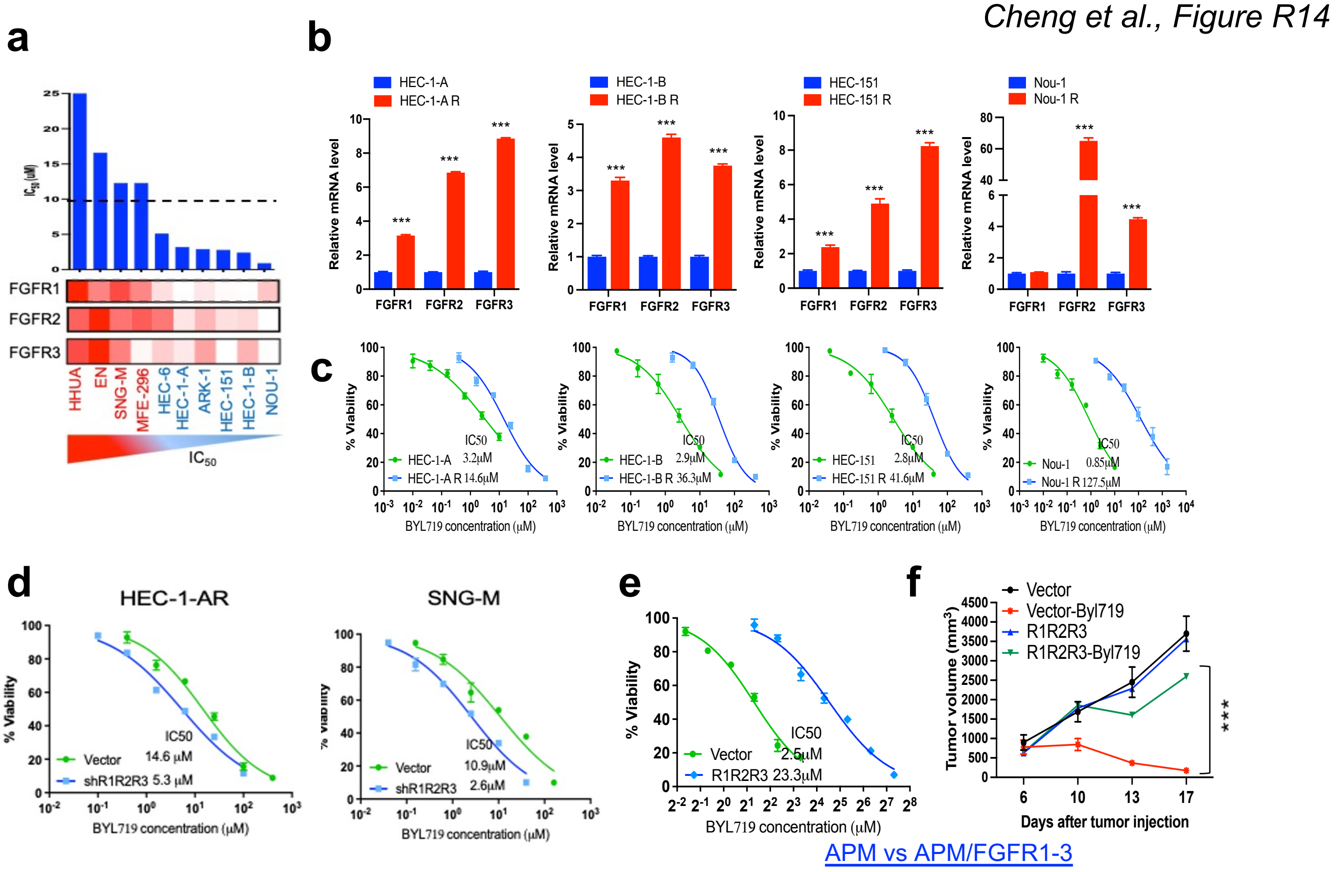

Figure R14. FGFR overexpression drives, and FGFR knockdown reverses, PI3Kα inhibitor resistance in endometrial cancer models.

(a) Relationship between FGFR expression and PI3Kα inhibitor sensitivity across a panel of human EC cell lines. Upper panel: BYL719 IC_{50} values for each line. Lower heatmap: relative mRNA expression of FGFR1-3 in the same lines. FGFR-high lines (e.g., EN, SNG-M) cluster among the least sensitive to BYL719.

(b) RT-qPCR analysis of FGFR1-3 expression in matched parental (blue) vs BYL719-resistant (red) EC cell lines (HEC-1-A vs HEC-1-AR, HEC-1-B vs HEC-1-BR, HEC-151 vs HEC-151R, Nou-1 vs Nou-1R). Acquired resistance is accompanied by marked upregulation of FGFR1-3 in all resistant derivatives.

(c) Dose-response curves to BYL719 in the matched parental and resistant cell line pairs shown in (b). Resistant lines exhibit right-shifted curves and increased IC_{50} values, confirming PI3Kα inhibitor resistance.

(d) shRNA-mediated knockdown of FGFR1-3 (shR1R2R3) sensitizes resistant models to PI3Kα inhibition. BYL719 dose-response curves are shown for HEC-1-AR and SNG-M cells. FGFR1-3 knockdown induced a leftward shift of the dose-response curves, reducing the IC_{50} from 14.6 μM (vector) to 5.3 μM (shR1R2R3) in HEC-1-AR cells and from 10.9 μM (vector) to 2.6 μM (shR1R2R3) in SNG-M cells.

(e) Overexpression of FGFR1-3 in APM cells (APM/FGFR1/2/3) confers resistance to BYL719 in vitro, as evidenced by increased IC_{50} values compared with vector-control APM tumor cells.

(f) In vivo validation using APM/FGFR1-3 allograft tumors. Tumor-bearing mice were treated with vehicle or BYL719 as indicated. FGFR1-3 overexpression confers resistance to BYL719, whereas tumors with control vector remain highly responsive. Data are mean ± SEM (n = 8-10 per group); statistical significance is indicated where applicable.

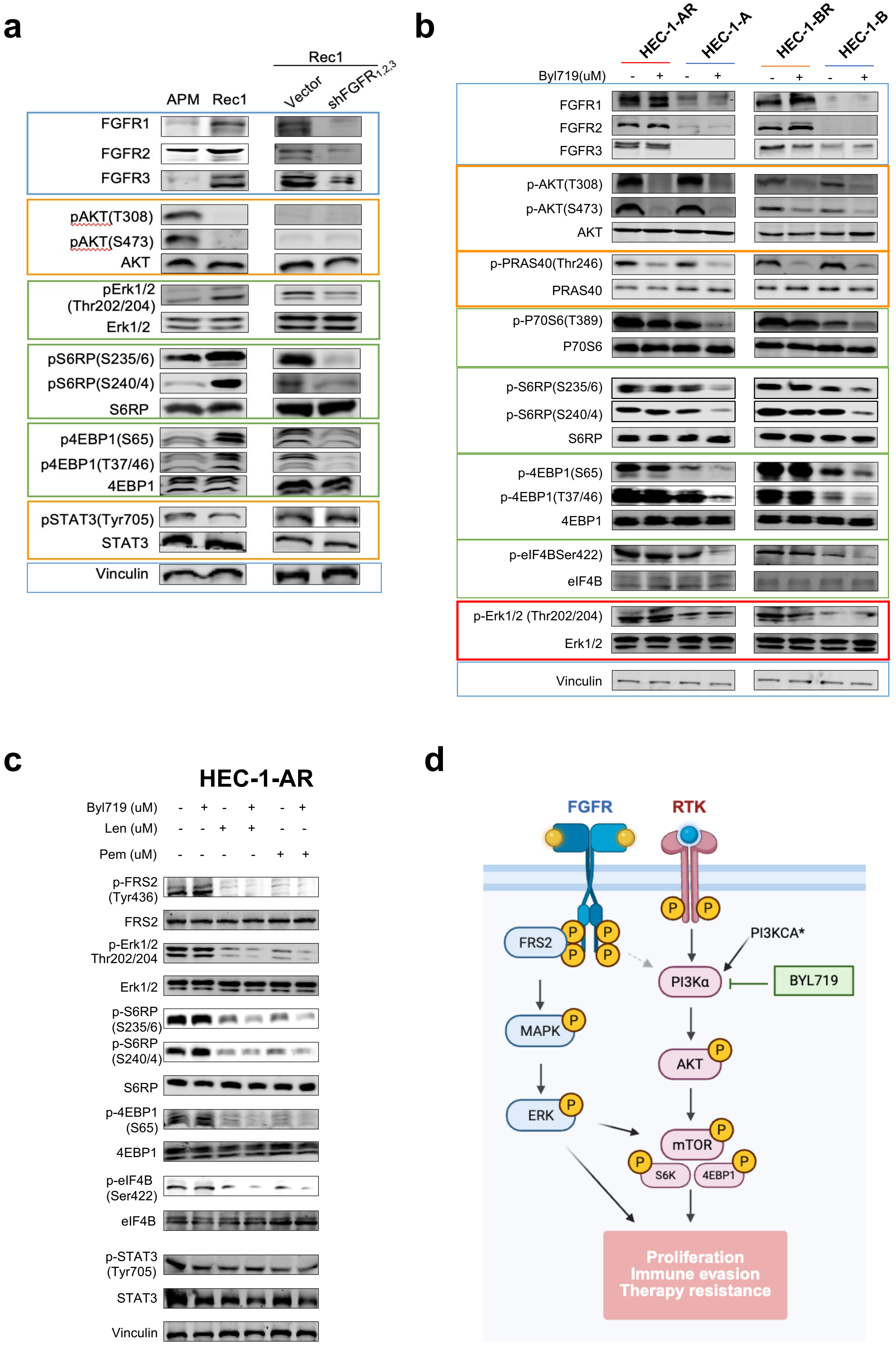
a
Rec1
APM
Rec1
Vector
shFGFR1,2,3
FGFR1
FGFR2
FGFR3
pAKT(T308)
pAKT(S473)
AKT
pErk1/2
(Thr202/204)
Erk1/2
pS6RP(S235/6)
pS6RP(S240/4)
S6RP
p4EBP1(S65)
p4EBP1(T37/46)
4EBP1
pSTAT3(Tyr705)
STAT3
Vinculin
b
HEC-1-AR
HEC-1-A
HEC-1-BR
HEC-1-B
Byl719(uM)
FGFR1
FGFR2
FGFR3
p-AKT(T308)
p-AKT(S473)
AKT
p-PRAS40(Thr246)
PRAS40
p-P70S6(T389)
P70S6
p-S6RP(S235/6)
p-S6RP(S240/4)
S6RP
p-4EBP1(S65)
p-4EBP1(T37/46)
4EBP1
p-eIF4BSer422)
eIF4B
p-Erk1/2 (Thr202/204)
Erk1/2
Vinculin
c
HEC-1-AR
Byl719 (uM)
Len (uM)
Pem (uM)
p-FRS2
(Tyr436)
FRS2
p-Erk1/2
Thr202/204
Erk1/2
p-S6RP
(S235/6)
p-S6RP
(S240/4)
S6RP
p-4EBP1
(S65)
4EBP1
p-eIF4B
(Ser422)
eIF4B
p-STAT3
(Tyr705)
STAT3
Vinculin
d
FGFR
RTK
FRS2
PI3KCA*
PI3Kα
BYL719
MAPK
AKT
ERK
mTOR
S6K
4EBP1
Proliferation
Immune evasion
Therapy resistance

Figure R15. Delineating downstream signaling pathways activated by FGFR in PI3Kα inhibitor-resistant endometrial cancer models.

(a) FGFR1-3 knockdown attenuates MAPK-mTOR signaling in FGFR-high recurrent Rec1 tumors. Western blot analysis of FGFR1-3 and downstream signaling pathways in lysates from primary APM tumors, FGFR-high recurrent Rec1 tumors, and Rec1 tumors expressing shRNA targeting FGFR1-3 (shFGFR1,2,3) or vector control. Compared with primary APM tumors, Rec1 tumors show increased FGFR1-3 expression accompanied by elevated phosphorylation of ERK1/2 (T202/Y204), S6RP (S235/236 and S240/244), and 4EBP1 (S65 and T37/46). Notably, p-AKT (T308/S473) is robust in APM tumors but is nearly undetectable in Rec1 tumors, consistent with inactivation of PIK3CA-H1047R despite FGFR upregulation. Silencing FGFR1-3 in Rec1 tumors markedly reduces p-ERK1/2 and phosphorylation of mTOR targets, whereas p-STAT3 levels remain largely unchanged. Vinculin serves as a loading control. These data indicate that FGFR upregulation sustains MAPK-mTOR signaling in recurrent tumors following PIK3CA-H1047 inactivation.

(b) Differential effects of PI3Kα inhibition on downstream signaling in BYL719-sensitive versus -resistant human EC cell lines. Western blot analysis of FGFR1-3, PI3K-AKT-mTOR, and ERK signaling in BYL719-sensitive HEC-1-A and HEC-1-B cells and their matched resistant derivatives HEC-1-AR and HEC-1-BR, treated with or without BYL719 (1 μM, 24 h). In both parental and resistant lines, BYL719 effectively suppresses p-AKT (T308/S473) and p-PRAS40 (T246). In contrast, phosphorylation of downstream mTOR effectors (p-P70S6K, p-S6RP S235/236 and S240/244, p-4EBP1 S65 and T37/46, and p-eIF4B S422) is markedly reduced only in sensitive cells and is largely maintained in resistant derivatives, indicating uncoupling of mTOR output from AKT in the resistant state. p-ERK1/2 (T202/Y204) is strongly elevated in resistant cells and shows minimal response to BYL719, consistent with MAPK pathway activation as a key adaptive resistance mechanism.

(c) FGFR inhibition suppresses MAPK-mTOR signaling, but not STAT3, in BYL719-resistant cells. Western blot analysis of HEC-1-AR cells treated with BYL719 (1 μM), lenvatinib (Len; 20 μM), pemigatinib (Pem; 2 μM), or the indicated combinations for 24 h. FGFR inhibition with lenvatinib or pemigatinib, particularly in combination with BYL719, markedly reduces phosphorylation of FRS2, ERK1/2, and downstream mTOR targets (S6RP, 4EBP1, eIF4B), whereas p-STAT3 levels remain largely unchanged, indicating that FGFR blockade primarily attenuates the FRS2-MAPK-mTOR axis in resistant cells.

(d) Proposed model of FGFR-driven resistance to PI3Kα-targeted therapy. Upregulation of FGFR signaling activates FRS adaptor proteins and the downstream MAPK-ERK pathway, which in turn sustains mTOR signaling (S6K and 4EBP1) independently of AKT. This FGFR-dependent MAPK-mTOR signaling axis supports tumor cell proliferation, immune evasion, resistance to PI3Kα inhibition, thereby driving persistent tumor growth and immune escape.

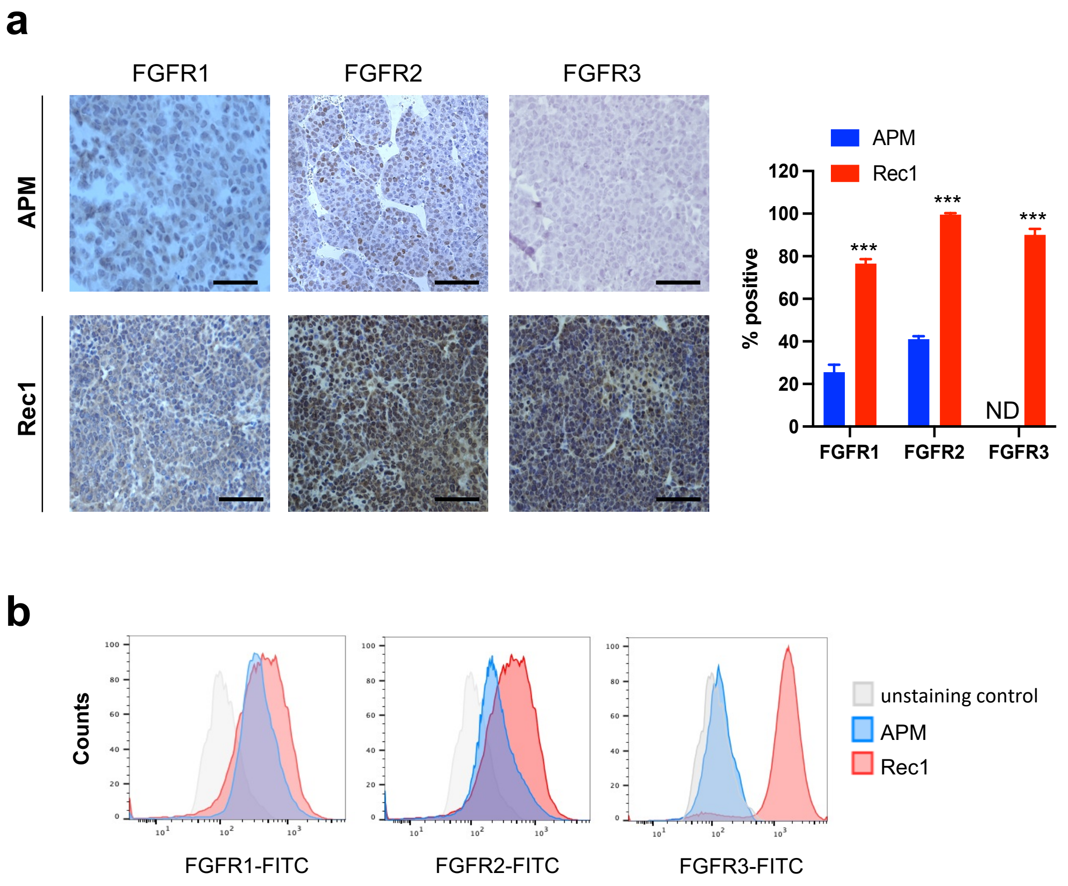

Figure R16. Orthogonal protein-level validation of FGFR1/2/3 expression in primary (APM) and recurrent (Rec1) tumors.

(a) Immunohistochemical staining of FGFR1, FGFR2, and FGFR3 in primary APM tumors (top) and recurrent Rec1 tumors (bottom). APM tumors show modest FGFR1 and FGFR2 staining and essentially no detectable FGFR3 expression, whereas Rec1 tumors display strong and diffuse positivity for all three FGFRs. Right, quantification of FGFR1/2/3-positive tumor cells (% positive) in APM and Rec1 sections (mean ± SEM). ND, not detectable for FGFR3 in APM. Scale bars, 50 μm.

(b) Flow cytometric analysis of surface FGFR1, FGFR2, and FGFR3 expression on dissociated tumor cells from APM (blue) and Rec1 (red) tumors, with unstained controls shown in gray. Histograms demonstrate a clear rightward shift in FGFR1, FGFR2, and particularly FGFR3 surface expression in Rec1 relative to APM, confirming at the protein level that primary APM tumors contain FGFR1/2^{low} and FGFR3^{negative} epithelial cell populations, whereas recurrent Rec1 tumors are enriched for FGFR1/2/3^{high} populations.

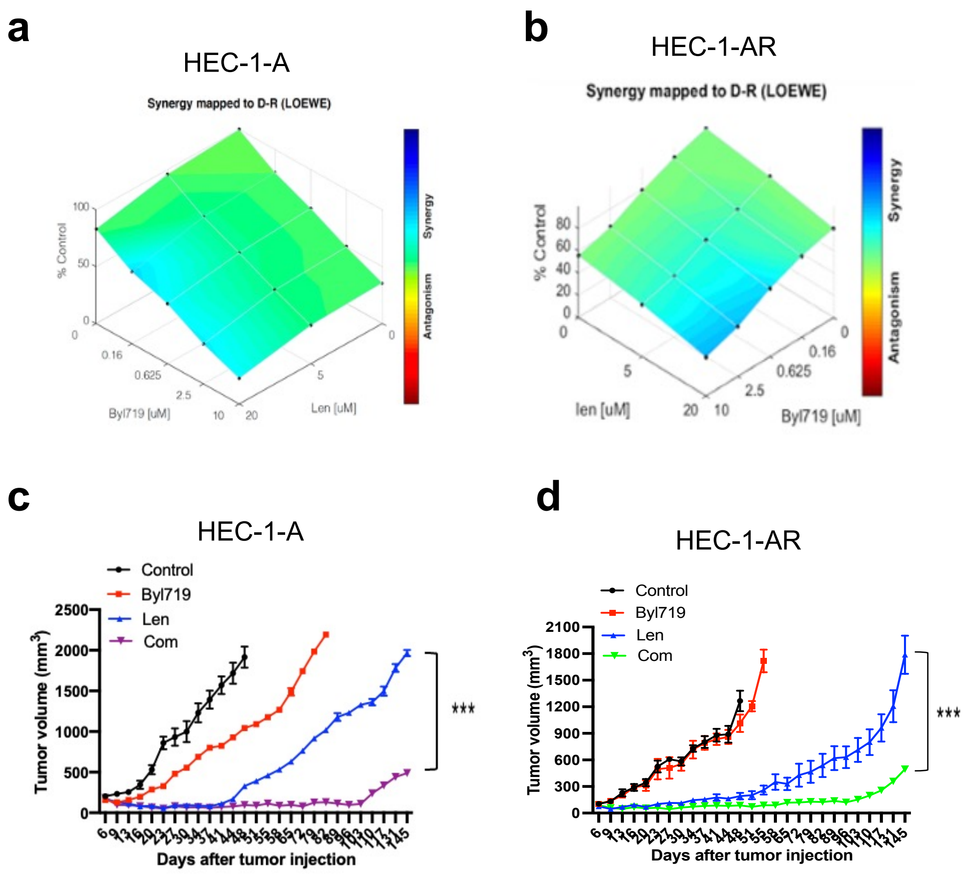

Figure R17. Effects of combined PI3Kα and FGFR inhibition in PI3Kα-sensitive and -resistant HEC-1-A models.

(a, b) Combenefit isobologram/synergy maps for BYL719-lenvatinib dose-matrix combinations in parental PI3Kα-sensitive HEC-1-A cells (a) and PI3Kα-resistant HEC-1-AR cells (b). Surfaces are mapped using the Loewe additivity model, in which blue indicates synergy and red indicates antagonism. An additive to mildly synergistic interaction is observed in parental cells, whereas a more pronounced synergistic region is evident in HEC-1-AR cells at clinically relevant concentrations.

(c, d) Tumor growth curves of xenografts derived from HEC-1-A (c) and HEC-1-AR (d) cells treated with vehicle control, BYL719 alone, lenvatinib alone, or the BYL719+lenvatinib combination (Com) (n = 8 per group; mean ± SEM). In both parental and resistant tumors, the combination produces greater tumor growth inhibition than either single agent, with a particularly marked effect in the resistant HEC-1-AR model, confirming that PI3Kα and FGFR co-inhibition is beneficial in both PI3Kα-sensitive and -resistant settings.

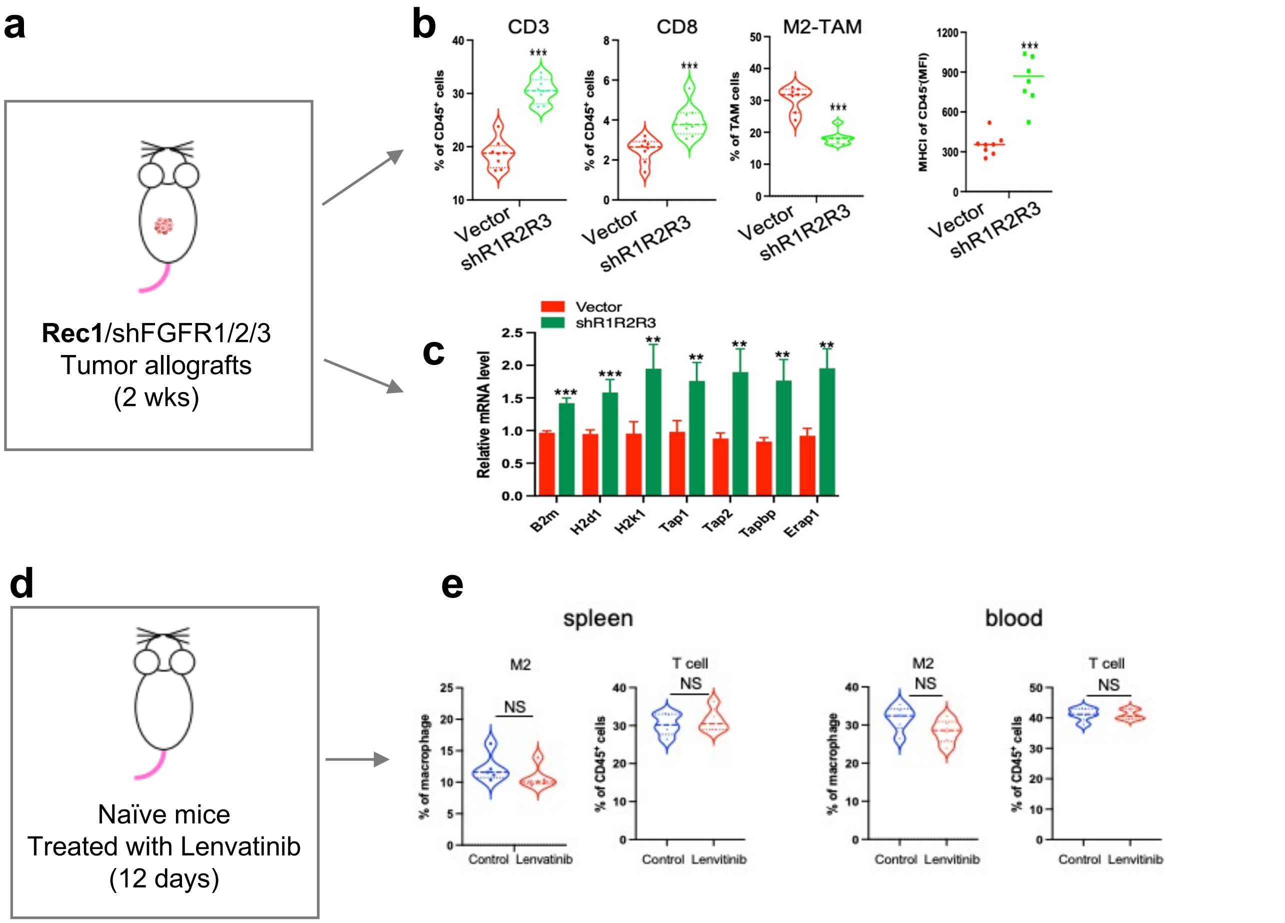

Figure R18. Tumor-intrinsic versus systemic effects of FGFR blockade on immune modulation in vivo.

(a) Schematic of the Rec1 tumor allograft model with stable, tumor-cell–intrinsic knockdown of FGFR1/2/3 (shFGFR1/2/3) or vector control.

(b) Flow cytometric analysis of Rec1 allografts showing frequencies of $CD3^+$ and $CD8^+$ T cells among $CD45^+$ cells, the proportion of M2-type TAMs, and MHC-I mean fluorescence intensity (MFI) on $CD45^-$ tumor cells in vector-control versus shFGFR1/2/3 tumors. Tumor-intrinsic FGFR depletion increases intratumoral $CD3^+$ and $CD8^+$ T cells, decreases M2-type TAMs, and enhances MHC-I expression, recapitulating the immune changes observed with systemic FGFR inhibition.

(c) RT-qPCR analysis of antigen processing and presentation genes (B2m, H2-d1, H2-k1, Tap1, Tap2, Tapbp, and Erap1) in Rec1 tumors expressing vector control or shFGFR1/2/3. Expression levels are normalized to vector controls and show coordinated upregulation of MHC-I machinery upon tumor-cell-intrinsic FGFR depletion.

(d) Schematic of naïve, tumor-free mice treated systemically with vehicle or lenvatinib for 12 days.

(e) Flow cytometric analysis of M2-like macrophages and T cells in the spleen and peripheral blood of naïve mice treated with vehicle or lenvatinib. No significant differences are observed between groups (n = 5 mice per group; NS, not significant), indicating that, in the absence of tumor, lenvatinib has minimal direct impact on systemic immune cell populations.

REDACTED: UNPUBLISHED DATA

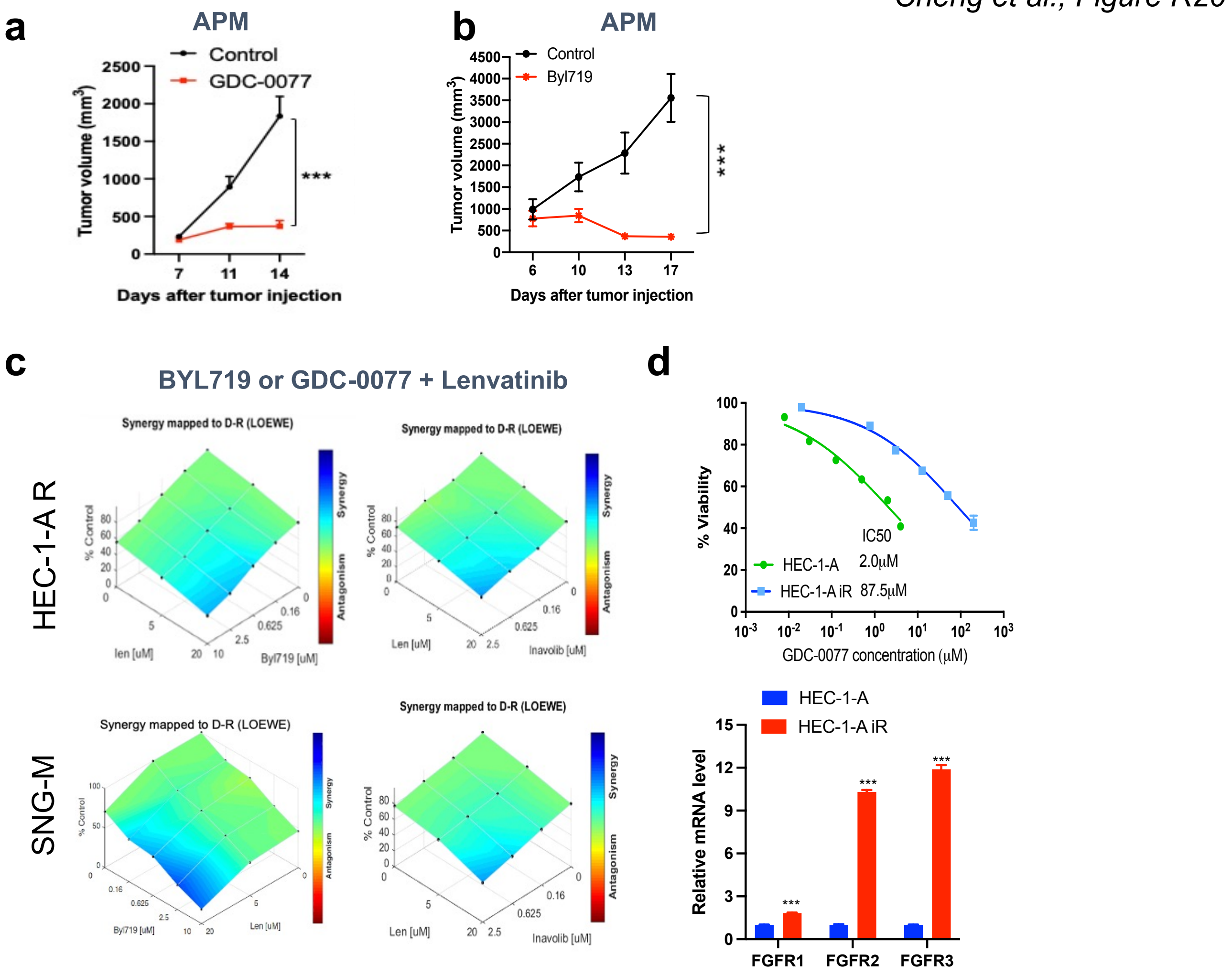

Figure R20. Extending PI3Kα targeting to the next-generation inhibitor inavolisib (GDC-0077).

(a, b) Tumor volume measurements of APM allografts treated with the first-generation PI3Kα inhibitor BYL719 (alpelisib) or the next-generation PI3Kα inhibitor GDC-0077 (inavolisib) compared with vehicle control (n = 8-10 per group). Both agents markedly suppress tumor growth relative to control.

(c) Combenefit isobologram plots showing interaction landscapes for BYL719+lenvatinib and inavolisib+lenvatinib dose-matrix combinations in FGFR-high HEC-1-AR and SNG-M EC cells. In both models, combining lenvatinib with either BYL719 or inavolisib yields additive to synergistic effects on cell viability, demonstrating that the benefit of PI3Kα/FGFR co-inhibition extends to the next-generation PI3Kα inhibitor.

(d) Generation and characterization of inavolisib-resistant HEC-1-A iR cells. Upper panel, dose-response curves to inavolisib in parental HEC-1-A and HEC-1-A iR cells, showing a pronounced rightward shift and increased IC_{50} in the resistant line. Lower panel, RT-qPCR analysis of FGFR1-3 expression demonstrating robust upregulation of FGFRs in HEC-1-A iR cells relative to parental HEC-1-A, analogous to the resistance mechanism observed with BYL719. Together, these data confirm that FGFR upregulation represents a shared resistance mechanism to both first- and next-generation PI3Kα inhibitors.
